# The Electronic Spin State of Diradicals Obtained from the Nuclear Perspective: The Strange Case of Chichibabin Radicals

**DOI:** 10.1002/cphc.202400707

**Published:** 2025-01-14

**Authors:** Gabriel Moise, Saleta Fernández, Kit Joll, Mikhail V. Vaganov, Fátima García, Christiane R. Timmel, Diego Peña, Arzhang Ardavan

**Affiliations:** ^1^ Centre for Advanced Electron Spin Resonance The Clarendon Laboratory Department of Physics University of Oxford Parks Road Oxford OX1 3PU United Kingdom; ^2^ Centro Singular de Investigación en Química Biolóxica e Materiais Moleculares Universidade de Santiago de Compostela C/Jenaro de la Fuente s/n (esquina Avda. Mestre Mateo), Campus Vida Santiago de Compostela 15705 Spain; ^3^ Centre for Advanced Electron Spin Resonance Inorganic Chemistry Laboratory University of Oxford South Parks Road Oxford OX1 3QR United Kingdom

**Keywords:** electron spin resonance, organic radicals, open-shell compounds, hyperfine couplings, electron nuclear double resonance

## Abstract

With a view towards the development of molecular spintronics, non‐linear optics, and qubits, a great amount of research effort aims to establish the factors which govern the spin classification of diradicals. Electron spin resonance (ESR) is an indispensable tool for such research. However, in some cases, the mere presence of an ESR spectrum is insufficient to ascertain that the presumed diradical is indeed a triplet state. In a comparative case study of a Chichibabin diradical and a monoradical analogue, we show how the signals from different spin states present in liquid solutions of these species may be disentangled. Ultimately, the correct spin classification depends on ESR techniques which probe the spin quantum number directly. In this work, electron nuclear double resonance experiments reveal that the nuclei provide a clear experimental probe of the electronic spin configuration.

## Graphical abstract

The electronic spin state of two Chichibabin radicals is investigated using 2‐dimensional hyperfine correlated Electron Nuclear Double Resonance. The protons (I=1/2) act as a reference for the electronic spin quantum number (S) via their hyperfine interactions. The monoradical system has an S=1/2 spin state whereas liquid solutions of the diradical system are shown to contain a mixture of species with S=1 and S=1/2.

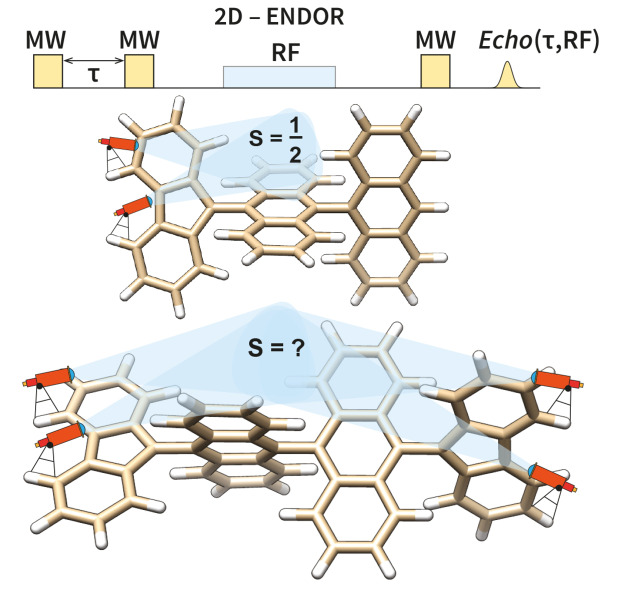

## Introduction

Polycyclic aromatic hydrocarbons (PAHs) represent a family of organic compounds formed by the fusion of aromatic rings.[^1^, ^2^, ^3^] In this work, we focus our attention on the open‐shell subclass of PAHs with one/two unpaired electrons. A variety of studies show that the presence of unpaired electrons in these species leads to a number of interesting properties such as: two photon absorption enhancement,^[4]^ singlet fission,^[5]^ interesting chiroptical,[^6^, ^7^] or amphoteric redox behaviour.^[8]^ These properties make open‐shell PAHs attractive for applications in the fields of materials science, spintronics, and quantum computing.[^9^, ^10^, ^11^, ^12^, ^13^] Accordingly, the design and synthesis of open‐shell PAHs is an active field of research.[^14^, ^15^, ^16^, ^17^, ^18^, ^19^] Among the many open‐shell PAHs synthesised to date, derivatives of Chichibabin's hydrocarbons stand out due to their stability under ambient conditions.^[20]^ Particular examples of such stable Chichibabin radicals are those bearing anthracene units as the central core.[^21^, ^22^, ^23^]

When it comes to characterising the properties of open‐shell PAHs, electron spin resonance (ESR) is a valuable technique because it directly probes the electron spin density of paramagnetic molecules. In its most popular implementation, continuous‐wave ESR (cwESR) is used as a simple analytical tool for ascertaining that a particular compound is paramagnetic. However, this question becomes more complicated when it comes to determining the spin states of compounds with more than one unpaired electron. For example, consider an organic diradical: the absence of cwESR signals is often attributed to the diamagnetic singlet state, while the presence of a cwESR signal is linked to the triplet state. However, the mere existence of a non‐zero cwESR spectrum is not sufficient to conclude that the observed paramagnetic species is the triplet state of the diradical. The correct classification of the spin state of diradicals requires a detailed characterisation of the various interactions present in the spin Hamiltonian. In particular, one requires pulse ESR experiments which can measure the total spin quantum number directly.

**Figure 1:**
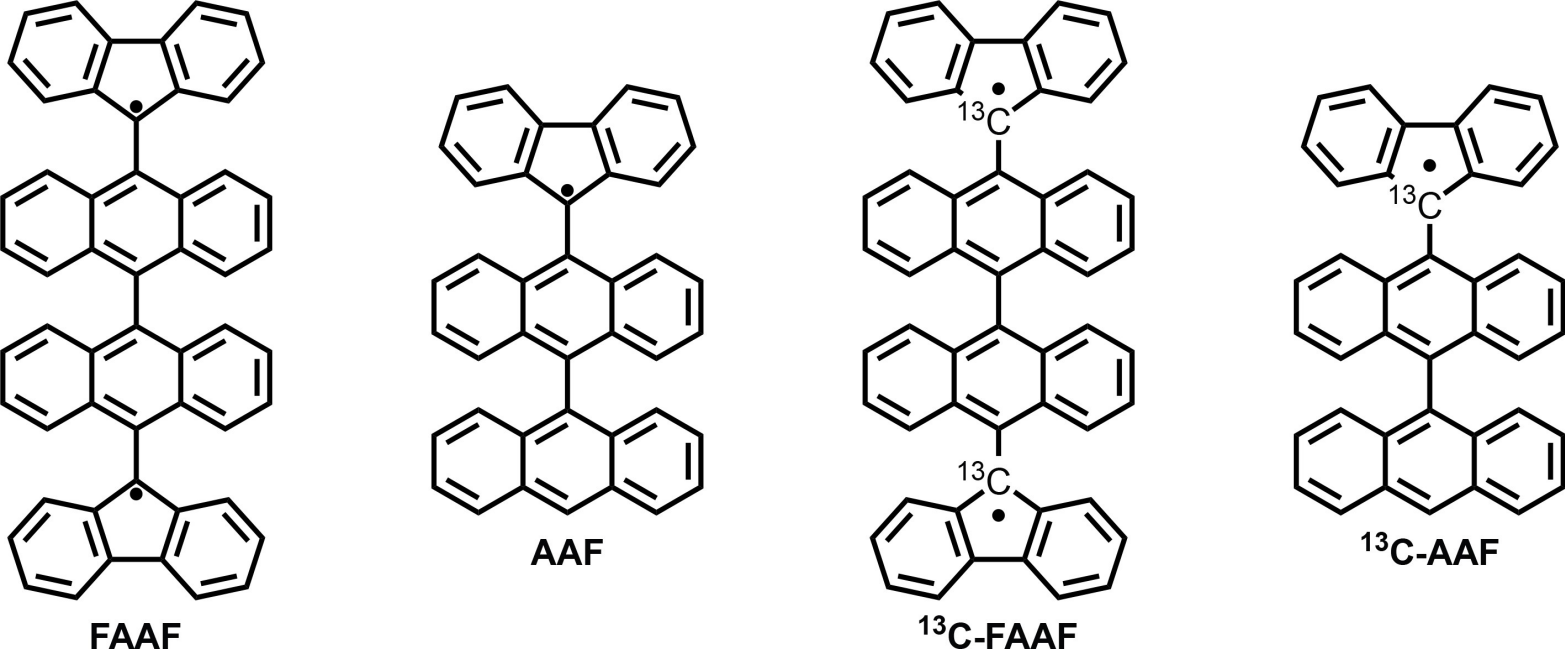
Structures of radical species studied in this work. The species are labelled as follows: **F** represents a fluorenyl fragment and **A** denotes an anthryl fragment.

In this work, we present a continuous‐wave, pulse ESR, and electron‐nuclear double resonance (ENDOR) case study of the Chichibabin radicals shown in Figure 1. These systems serve as model compounds for the wider class of open‐shell PAHs. Given the above mentioned intricacies of classifying the spin states of diradicals, the focus of the study is on understanding the behaviour of liquid solutions of the **FAAF** molecule, which was previously investigated by Zeng et al.^[21]^ The **AAF** monoradical, which benefits from a well‐defined electronic spin‐1/2, will serve as an essential reference point for the ESR data obtained for **FAAF**. Additionally, the 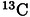 substituted derivatives benefit from larger hyperfine couplings which facilitate the deconvolution of the cwESR spectra.

**Figure 2:**
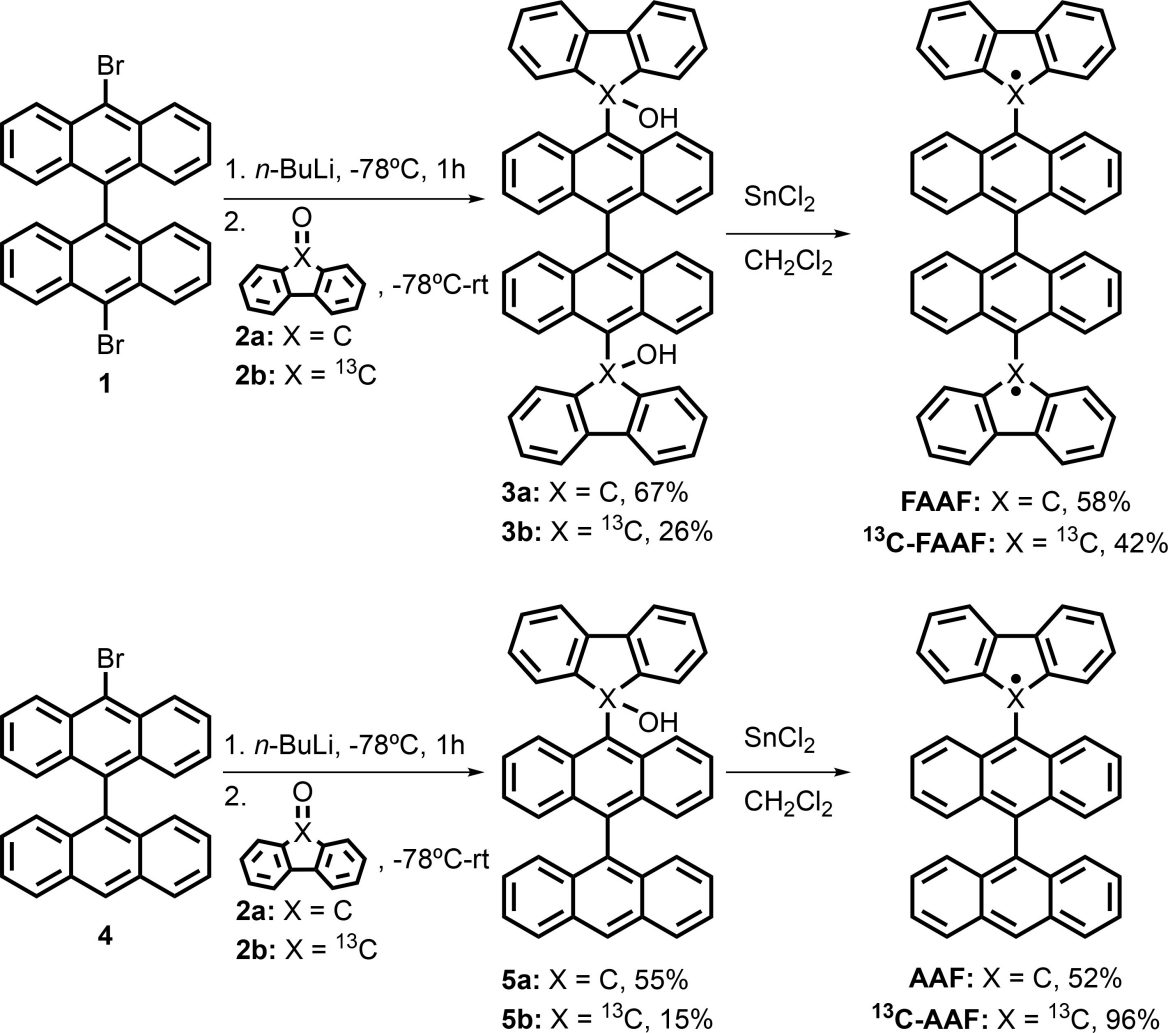
Synthesis of diradicals **FAAF** (top) and monoradicals **AAF** (bottom).

The diradical **FAAF** was synthesized following the procedure reported by Zeng et al. (Figure 2).^[21]^ Specifically, 10,10’‐dibromo‐9,9’‐bianthracene (**1**) was treated sequentially with *n*‐BuLi and fluorenone (**2** **a**) to yield diol (**3** **a**). Then, reaction of compound **3** **a** with ${\mathrm{SnCl}}_{2}$ afforded the diradical **FAAF**, which is stable enough to be purified by column chromatography and isolated under ambient conditions. A similar synthetic procedure was used for the preparation of monoradical **AAF**, from 10‐bromo‐9,9’‐bianthracene (**4**). In this case, **AAF** was isolated in a 52 % yield by reaction of alcohol **5** **a** with ${\mathrm{SnCl}}_{2}$ .^[24]^ For the preparation of isotopically labelled radicals 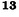 **C‐FAAF** and 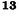 **C‐AAF**, it was first necessary to prepare the labelled fluorenone **2** **b** (see SI for details). Then, the corresponding isotopically labelled radicals were obtained following the same procedure as for the preparation of **FAAF**.

## Results and Discussion

### Continuous‐Wave ESR

**Figure 3:**
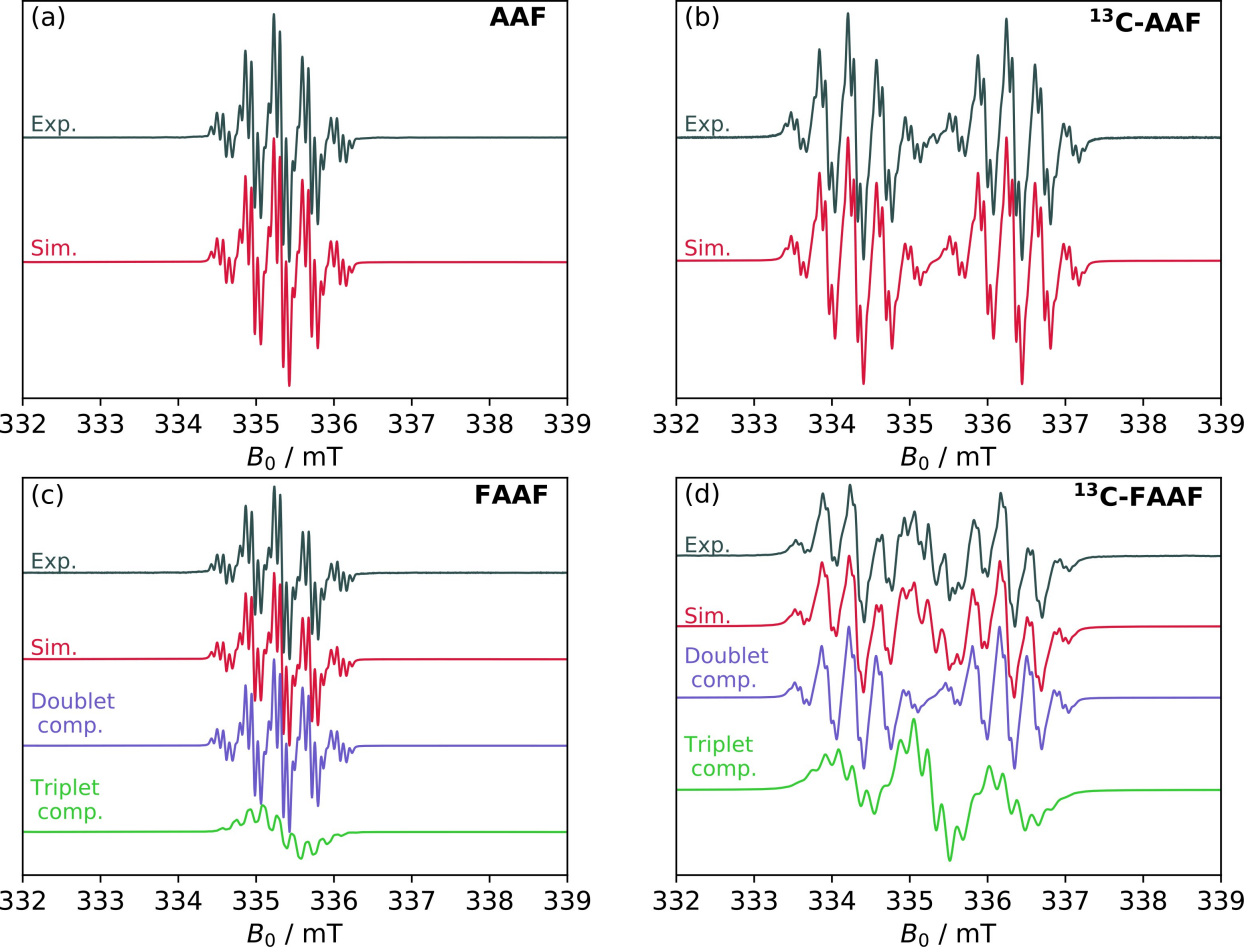
CwESR data and simulations obtained at room temperature in liquid solutions using dichloromethane as the solvent. (a, b) Correspond to the **AAF** system with and without 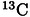 labelling, as indicated. (c, d) Correspond to the **FAAF** system with and without 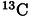 labelling. The simulations of the **FAAF** spectra are comprised of a linear combination of a doublet spectrum (blue lines) and a triplet spectrum (green lines). The corresponding simulation parameters are displayed in Table 1.

The initial discussion will focus on the room temperature cwESR results obtained for the monoradical reference system, **AAF**, in liquid solutions of dichloromethane. The experimental and simulated cwESR spectra are shown in Figure 3. A compilation of simulation parameters is shown in Table 1. Complete details regarding acquisition, sample preparation, and data analysis are given in the SI.

**Table 1:** CwESR simulation parameters: isotropic hyperfine couplings ($a_{\mathrm{iso}}$ in MHz), Lorentzian and Gaussian broadenings (FWHM: $\sigma_{L}$ and $\sigma_{G}$ in mT), and isotropic *g*‐factors. For the unlabelled **FAAF** spectrum, we find a relative doublet/triplet weighting of 77 %/23 %. For the labelled **FAAF** spectrum, the weighting was approximately 50 % for both components. Note: establishing the relative weighting of these components is not the aim of this work; we are primarily interested in methods that can ascertain the spin state of a molecule.

**FAAF** – doublet		**FAAF** – triplet		**AAF**	
Parameter	Value	Parameter	Value	Parameter	Value
$a_{\mathrm{iso}}$ (1×^13^C)	54.3	$a_{\mathrm{iso}}$ (2×^13^C)	27.1± 0.27	$a_{\mathrm{iso}}$ (1×^13^C)	57.10
$a_{\mathrm{iso}}$ (4×^1^H)	10.01± 0.40	$a_{\mathrm{iso}}$ (8×^1^H)	4.80± 0.45	$a_{\mathrm{iso}}$ (4×^1^H)	10.25± 0.26
$a_{\mathrm{iso}}$ (2×^1^H)	2.27± 0.23	$a_{\mathrm{iso}}$ (2×^1^H)	1.60± 0.65	$a_{\mathrm{iso}}$ (2×^1^H)	2.37± 0.15
$a_{\mathrm{iso}}$ (2×^1^H)	1.81± 0.12			$a_{\mathrm{iso}}$ (2×^1^H)	1.80± 0.06
$\sigma_{L}$	0.06	$\sigma_{L}$	0.06	$\sigma_{L}$	0.04
$\sigma_{G}$	0.03	$\sigma_{G}$	0.03	$\sigma_{G}$	0.03
g‐factor	2.0028	g‐factor	2.0029	g‐factor	2.0028

The room temperature spectra of the 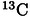 labelled and unlabelled **AAF** systems are typical of organic radicals with transitions centred around $g_{e}$ ≈2.0023. The largest hyperfine splitting in panel (a) is caused by couplings to four approximately equivalent protons with a Fermi contact interaction $a_{\mathrm{iso}}$ ≈10 MHz. For the labelled system in panel (b), a Fermi contact interaction of ~57 MHz to 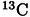 causes a “doubling” of the spectrum. These hyperfine parameters are broadly consistent with the Density Functional Theory (DFT) results shown in the SI. The DFT calculation predicts a spin density localised primarily on the fluorenyl (**F**) side of the molecule with minimal leakage onto the anthryl fragments. According to DFT, the four 10 MHz 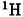 couplings belong to the *ortho* and *para* protons relative to the carbon atom with the largest spin density – *i. e*. marked with a dot in Figure 1 (see also ENDOR discussion below). The next largest DFT predicted couplings correspond to the *meta* protons, and are represented in the spectral simulations as the two additional sets of two protons with couplings of ca. 2 MHz.

It is important to highlight the remarkable quality of the simulations of the **AAF** spectra in panels (a) and (b) of Figure 3. The RMSD of the best fit is ~0.5 %. Such a good fit is not possible for the spectra of **FAAF** in a *single component* simulation. The reason why single‐component simulations fail for **FAAF** can be most easily understood by visual inspection of the data in panel (d). A hyperfine coupling to two equivalent 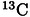 nuclei ought to produce a spectrum which is the convolution of the unlabelled spectrum – panel (c) – with a 1 : 2 : 1 splitting pattern caused by the larger 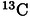 couplings. Instead, the spectrum in panel (d) appears to be an overlap of the spectrum in panel (b) with another spectrum which has the expected 1 : 2 : 1 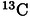 splitting pattern.

The resolution of this conundrum was only reached after analysing the pulse ESR results (see below). The conclusion is that the **FAAF** spectra in Figure 3 do indeed arise from a *mixture* of a doublet component, akin to **AAF**, and a triplet component (see also Table 1). The doublet component tends to dominate the spectra with a weighting of 50–90 %. Importantly, its exact weighting is not reproducible between different samples; it is non‐trivially dependant on preparation conditions. In the SI, we explore further the effects of different parameters (oxygen, solvent, light, temperature, storage times) on the appearance of the spectra and on the relative weighting of the doublet and triplet components. Although the results are not conclusive, the doublet component observed in liquid solutions of **FAAF** is likely caused by a combination of the following: (1) interactions with molecular oxygen, (2) interactions with light, (3) interactions with the solvent, and (4) intermolecular interactions (*e. g*. π‐stacking). All these factors are also consitent with a degradation of the triplet state to another fluorenyl‐based doublet species.

Although the doublet/triplet ratio is not reproducible, the hyperfine couplings of the two components are persistently the same between different samples, preparation methods, and storage times. Importantly, all the hyperfine couplings of the triplet component are approximately half of those in the doublet component. As discussed in the SI, this halving of the hyperfine interaction in the triplet is reproduced by the DFT results and it can also be explained intuitively by invoking the Pauli exclusion principle.

### Pulse ESR

**Figure 4:**
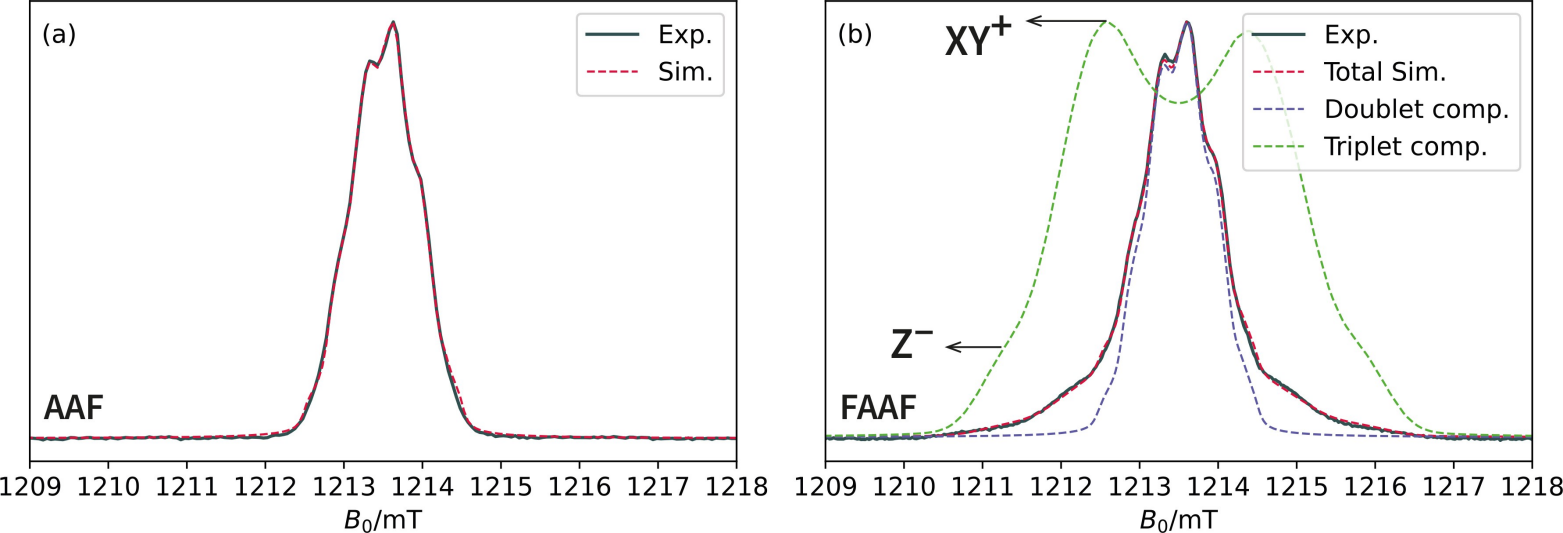
(a) FID field swept experimental and simulated spectrum of **AAF** recorded at 80 K and Q‐band using deuterated toluene as the solvent. (b) Experimental and simulated FID spectrum of the **FAAF** system. The simulation was achieved with a doublet/triplet weighting of 85 %/15 %. A summary of the simulation parameters is shown in Table 2; full simulation scripts are in the SI. For the triplet component of **FAAF** the anisotropy of the dipolar interaction, *i. e*. the D ‐tensor, is responsible for the Pake pattern appearance of the dashed green spectrum in panel (b). The turning points on the left of the spectrum are labelled as $Z^{-}$ and $XY^{+}$ (the corresponding labels on the right of the spectrum would be $Z^{+}$ and $XY^{-}$ ). The Z label represents resonance fields corresponding to molecules for which the $D_{z}$ ‐axis of the D ‐tensor is parallel to the external magnetic field, whereas the XY label refers to molecules with $D_{z}$ perpendicular to the magnetic field. The superscripts, ± , correspond to the $T_{-}\to T_{0}$ and $T_{0}\to T_{+}$ electron spin transitions, respectively.

Whilst compelling, the arguments of the previous section rely on two leaps‐of‐faith. Firstly, one has to accept that the spectrum of **FAAF** is a superposition of a doublet and triplet component and secondly, one has to trust that the 30 parameter cwESR fitting results are unique. The first independent piece of evidence in support of the doublet‐triplet admixture comes from the free induction decay (FID) spectra in Figure 4 and the associated simulation parameters in Table 2. Whilst for cwESR, the difference between **AAF** and **FAAF** was clearest when examining the 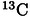 ‐labelled spectra, the unlabelled species offer the best comparison of the FID spectra of frozen solutions. In frozen solutions, the significant anisotropy of the 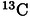 hyperfine tensor masks the fine‐structure of the triplet component of **FAAF**. The FID spectra of the 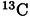 labelled species are shown in the SI, together with the results of hyperfine sub‐level correlation spectroscopy which validates our claim about the significant anisotropy of the 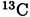 coupling. Therefore, the remaining analysis will focus only on the unlabelled species.

**Table 2:** Simulation parameters used to obtain the results in Figure 4. For **FAAF**, the spectrum was simulated using an 85 % triplet component. The spectra were simulated by employing isotropic proton hyperfine couplings and an anisotropic g‐tensor with eigenvalues $g_{1}$ , $g_{2}$ , and $g_{3}$ . The dipolar coupling of the triplet component of **FAAF** was taken into account via the *D*‐value. Simulations with anisotropic hyperfine couplings were also attempted, however, the number of parameters is very large and renders the interpretation meaningless. It is clear that the asymmetry of the experimental FID spectra is modelled accurately only by the anisotropy of the g‐tensor.

**FAAF** – doublet		**FAAF** – triplet		**AAF**	
Parameter	Value	Parameter	Value	Parameter	Value
$a_{\mathrm{iso}}$ (4×^1^H)/MHz	10.2	$a_{\mathrm{iso}}$ (8×^1^H)/MHz	4.9	$a_{\mathrm{iso}}$ (4×^1^H)/MHz	10.2
$\sigma_{L}$ /mT	0.10	$\sigma_{L}$ /mT	0.10	$\sigma_{L}$ /mT	0.10
$\sigma_{G}$ /mT	0.08	$\sigma_{G}$ /mT	0.08	$\sigma_{G}$ /mT	0.08
$g_{1}$	2.00231	$g_{1}$	2.00240	$g_{1}$	2.00226
$g_{2}$	2.00172	$g_{2}$	2.00119	$g_{2}$	2.00170
$g_{3}$	2.00136	$g_{3}$	2.00170	$g_{3}$	2.00131
–	–	D /MHz	−72 MHz	–	–

The interpretation of the **AAF** FID data is simple: the spectrum is modelled excellently by four almost equivalent proton hyperfines of ca. 10 MHz. The slight asymmetry of the spectrum relative to its barycentre is most easily quantified by a slightly anisotropic g ‐tensor (Table 2). However, the FID spectrum of **FAAF** is now clearly distinct from **AAF**. The triplet component appears as the broad shoulders to either side of the central doublet component which is otherwise akin to **AAF**. Similarly to cwESR, the weighting of the doublet is dominant (85 % in this particular case). The relative weighting of the two components obtained from this data may be slightly erroneous due to the fact that doublets and triplets have different nutation frequencies and different relaxation times. Yet, as for the cwESR data, the doublet weighting is not consistently the same between different samples. Nonetheless, all other simulation parameters remain reproducible. Particularly comforting is the fact that the halving of the triplet hyperfine couplings observed in the cwESR data is also reproduced in the FID data.

The biggest difference between the liquid solution cwESR and frozen solution FID spectra comes from the fact that the anisotropy of the dipolar coupling of the triplet component is resolved in the latter. This interaction is responsible for the $Z^{\pm }$ and $XY^{\pm }$ turning points depicted in panel (b) of Figure  4. As shown in the SI, the spacing between the $Z^{-}$ and $Z^{+}$ turning points of the triplet component is equivalent to 2|D| , where D is the dipolar coupling (related to the maximum, $D_{z}$ , eigenvalue of the dipolar coupling tensor by $D=\left(3/2\right)D_{z}$ ). The sign of D was chosen to be negative in these simulations, in keeping with the prolate spin density of **FAAF**. The magnitude of D is related to the average interspin distance between the two unpaired electrons of **FAAF**. A “back‐of‐the‐envelope” calculation, based on the simplified formula: |D| ≈490 nm^3^ MHz/${\left\langle r\right\rangle }^{3}$ , predicts an average interspin distance ⟨r⟩ ≈1.89 nm. This estimated distance is consistent with the DFT optimised geometry for the gas phase structure of **FAAF** which has a distance of 1.8964 nm between the centres of the fluorenyl (**F**) fragments. Extracting ⟨r⟩ in this simplified manner may lead to discrepancies caused by the exchange‐type integrals contributing to the *D*‐value (this is *not* the exchange interaction, *J*).^[25]^ A better estimate of the dipolar interaction is provided by calculating the *D*‐value using the DFT spin density. Such a calculation predicts that *D*=−67.5 MHz which is comparable to the experimental value of −72 MHz with a strain of ±5 MHz.

While the analysis of the FID spectra is more convincing than cwESR regarding the presence of a doublet/triplet admixture in solutions of **FAAF**, there is still a need for an experiment which proves conclusively and without relying on multi‐parameter simulations that this admixture is truly present. Given that the admixture is already ‘suspected’, the experimental test is trivial: the nutation frequencies of the two components should be related by a factor of $\sqrt{2}$ (see SI). This effect is clearly observed in the nutation data presented in the SI, thus validating the conclusions from both cwESR and FID experiments.

One of the reasons why the nutation experiment mentioned above was successful is that solutions of **FAAF** contain a doublet‐triplet admixture which allows the nutation frequencies to be compared. However, in general terms, this method would not work if the sample contained species with the same total spin quantum number. The nutation experiment requires a reference frequency. Such a reference may be obtained either by a precise calibration of the microwave power or the deliberate mixing of a species with known spin quantum number into the unknown sample. These methods are useful and should be employed whenever it is convenient/necessary to do so. However, below we propose and deploy a method based on ENDOR which can also be used to disentangle the spin states present in liquid solutions of multi‐radical systems.

### Electron Nuclear Double Resonance

**Figure 5:**
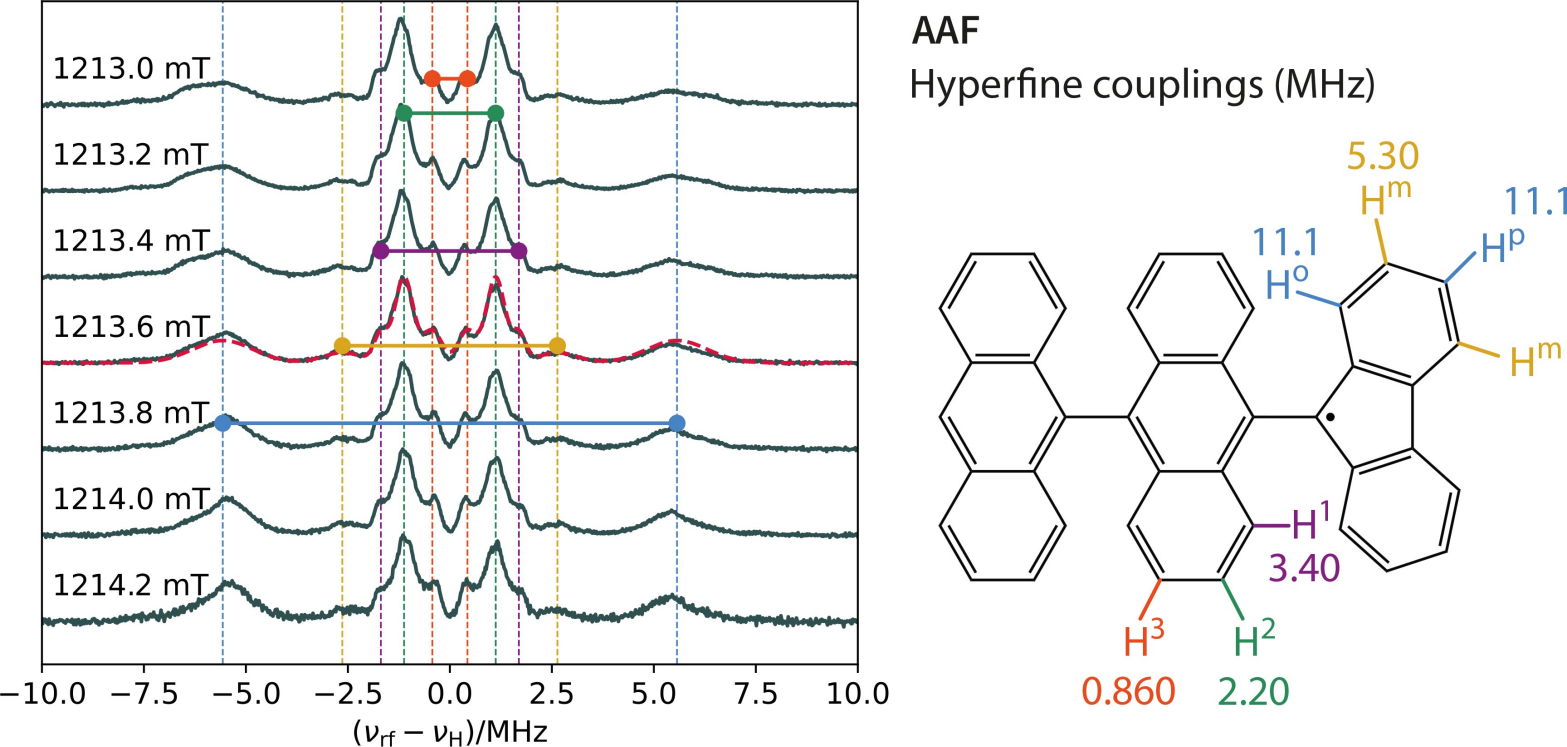
One‐dimensional Mims ENDOR spectra of **AAF** recorded across the ESR spectrum starting from 1213.0 mT in steps of 0.2 mT. The maximum in the ESR spectrum (see Figure 4) corresponds to the ENDOR spectrum at 1213.6 mT. The dashed red line superimposed on the 1213.6 mT data represents the best‐fitting of a sum of 5 symmetric bimodal Gaussian functions. The centres of the bimodal Gaussians are denoted by the coloured circles; the gaps between these circles correspond to the magnitudes of the hyperfine couplings. These couplings are assigned to the protons of the **AAF** molecules as depicted by the colours in the skeletal structure on the right. The assignment was done by comparison of the experimental values with the DFT computed hyperfine tensors.

**Figure 6:**
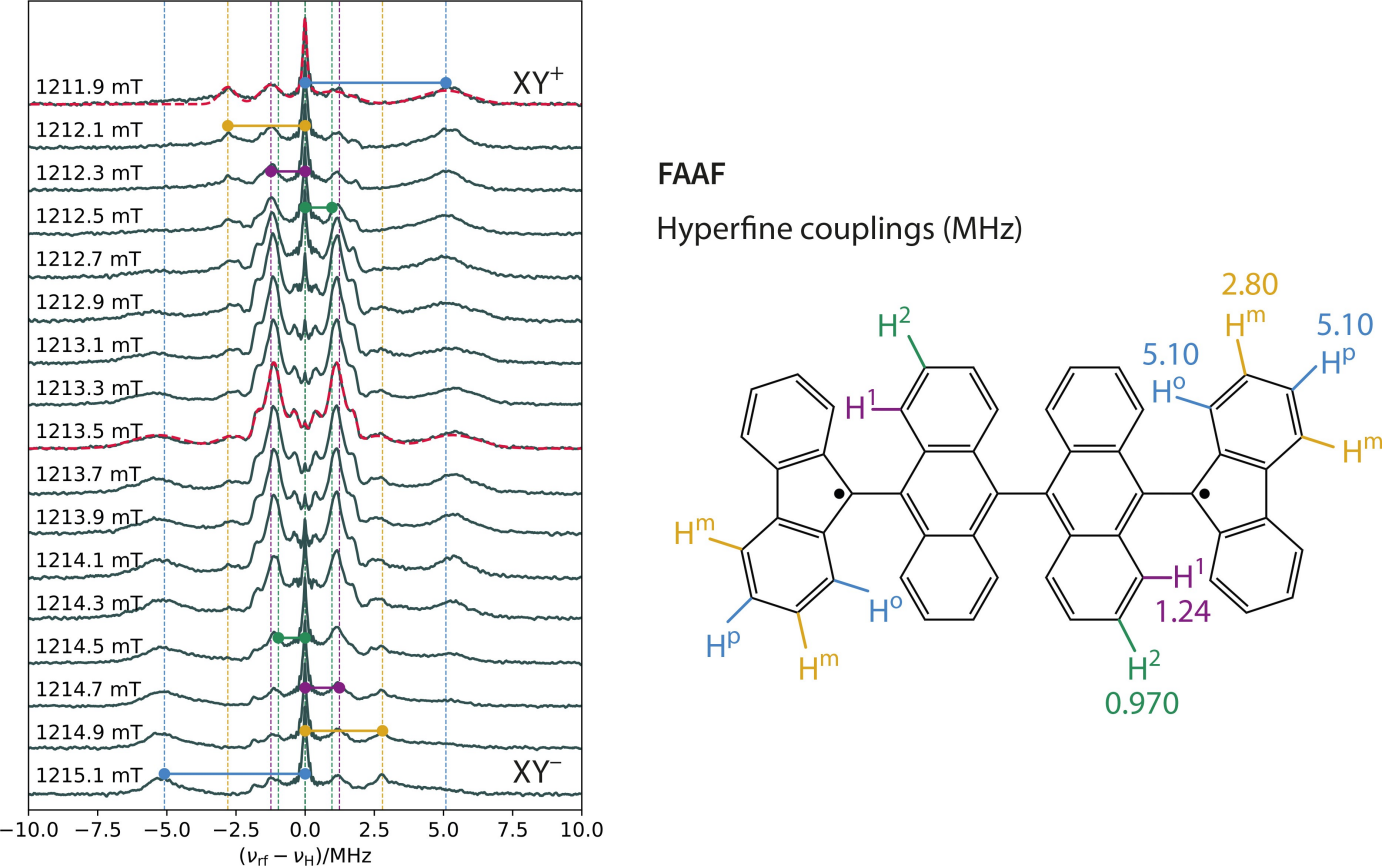
One‐dimensional Mims ENDOR spectra of **FAAF** recorded across the ESR spectrum. The maximum in the ESR spectrum (see Figure 4) corresponds to the ENDOR spectrum at 1213.5 mT. The dashed red line superimposed on the 1213.5 mT data represents the best‐fitting of a sum of 5 symmetric bimodal Gaussian functions. By contrast, the dashed red line superimposed on the 1211.9 mT data represents the best‐fitting of a sum of 5 (single mode) Gaussian functions. Similarly to Figure 5, the hyperfine couplings were assigned to the protons of **FAAF** by comparison with the DFT results. At the central field positions (1212.7–1214.3 mT), the ENDOR spectra of **FAAF** are very similar to the **AAF** (except for the appearance of the Larmor peak). However, the peripheral spectra, corresponding to the shoulders observed in the **FAAF** spectrum in Figure 4, are strongly indicative of a triplet state (strong Larmor peak and pronounced asymmetry). As mentioned in the SI, the sign of the hyperfine coupling can be determined for triplet states if the sign of the *D*‐value is known. The data shows that the *ortho*/*para* protons have a negative hyperfine coupling (*i. e*. an excess of β spin density) and the *meta* protons have a positive hyperfine coupling (*i. e*. an excess of α spin density). These relative signs are entirely reproduced by the phase of the DFT computed spin density.

Figures 5 and 6 summarise the 1D‐ENDOR results for **AAF** and **FAAF**. These ENDOR spectra were acquired across the ESR spectrum using microwave pulse lengths of 54 ns with a bandwidth of ca. 22 MHz (0.8 mT in field domain). For **AAF**, this pulse bandwidth is responsible for the small orientation selection effects observed in the ENDOR spectra acquired at different field positions. Apart from these small differences, the **AAF** ENDOR spectra are symmetric relative to the Larmor frequency ($\nu_{\mathrm{rf}}-\nu_{H}=0$ ), as expected for a doublet. The observed peaks are assigned to particular protons in **AAF** by performing a Gaussian deconvolution and comparing the results with the DFT predicted hyperfine tensors. As was the case for all data up to this point, the largest hyperfine coupling (blue line in Figure 5) corresponds to the *ortho*/*para* protons marked in the skeletal structures. The smaller hyperfine couplings to the *meta* protons and the anthryl protons are also clearly resolved in the data.

By contrast with **AAF**, the ENDOR spectra of **FAAF** (Figure 6) depend strongly on field position. At the low‐field side, the *ortho*/*para* protons give rise to a single peak centred at +5 MHz. The *meta* protons produce the peak at −2.5 MHz. As the field position approaches the centre of the ESR spectrum, the ENDOR data becomes more symmetric relative to the Larmor frequency. At the centre fields (1212.9–1214.1 mT), the **FAAF** spectra are effectively identical to **AAF** (apart from the Larmor peak). As the high‐field side of the ESR spectrum is approached, the ENDOR spectra become almost perfect mirror images of the corresponding low‐field spectra. As explained in the SI, the presence of the Larmor peak and the mirror image symmetry of the low/high‐field ENDOR spectra are signatures of a triplet state. The only problem is the similarity of the **FAAF** and **AAF** data at the centre fields. In the absence of other evidence, the data at 1213.5 mT would be consistent *both* with a doublet/triplet admixture and with a *pure* triplet state. This is because, when the anisotropy of the dipolar coupling is taken into account, both triplet transitions $T_{0}\to T_{+}$ and $T_{0}\to T_{-}$ are excited at the centre field position (*i. e*. at the mid‐point between $XY^{+}$ and $XY^{-}$ – Figure 4 – corresponding to an angle of 54.7° between $D_{z}$ and the magnetic field).

**Figure 7:**
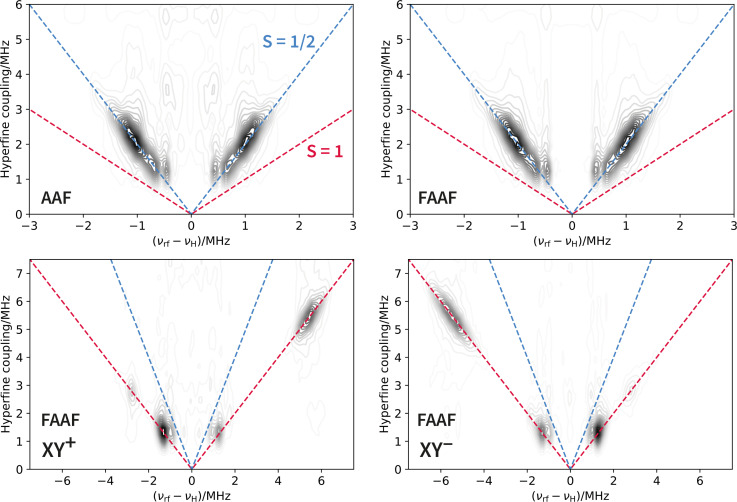
Hyperfine‐correlated ENDOR results for **AAF** and **FAAF**. (Top left) Data obtained at the central field position (1213.6 mT) of the **AAF** FID spectrum. The correlation ridges are observed along the dashed blue S=1/2 line corresponding to a hyperfine coupling equal to $2|\nu_{\mathrm{rf}}-\nu_{H}|$ . (Top right) Data collected at the central field position of the **FAAF** FID spectrum (1213.5 mT). (Bottom left/right) Data collected on the two left/right shoulders of the **FAAF** FID spectrum at 1211.9 and 1215.1 mT, respectively. The correlation ridges are along the dashed red S=1 line corresponding to hyperfine coupling magnitudes equal to $|\nu_{\mathrm{rf}}-\nu_{H}|$ . The mirror image symmetry between the two panels originates in the same effect as discussed for the 1D ENDOR spectra.

Hence, a pure triplet ENDOR spectrum recorded at the centre field would contain a Larmor peak and hyperfine peaks on either side of it, as would the ENDOR spectrum of a doublet/triplet admixture. Nonetheless, we already know from all the previously presented results that the admixture is truly the origin of the similarity between the ENDOR spectra of **AAF** and **FAAF** at the centre field position. However, in more complicated systems, such clear spectral simulations and nutation experiments are the exception, not the rule. In those circumstances, how could we test, experimentally, whether the 1213.5 mT ENDOR spectrum in Figure 6 is caused by a doublet/triplet admixture or a pure triplet? The answer lies in the hyperfine‐correlated ENDOR experiment shown in Figure 7.

As explained in the SI, hyperfine‐correlated ENDOR produces 2D spectra where the peaks in the usual 1D‐ENDOR spectra are correlated with the hyperfine couplings from which they originate. A doublet electronic spin coupled to multiple protons will give rise to hyperfine correlation ridges along the $2|\nu_{\mathrm{rf}}-\nu_{H}|$ line in the hyperfine dimension. This line is shown in dashed blue in Figure 7. For **AAF**, the data in the top left panel is fully consistent with an electronic spin quantum number of 1/2. When the measurement is performed on the central field position of the **FAAF** spectrum, the result is effectively identical to **AAF**. A triplet electronic spin produces hyperfine correlation ridges along the $|\nu_{\mathrm{rf}}-\nu_{H}|$ line (dashed red in Figure 7). The bottom panels in Figure 7 show that the assignment of a triplet state to the shoulders in the pulse ESR spectra of **FAAF** is correct. Overall, the hyperfine‐correlated ENDOR experiment demonstrates uniquely, without relying on subjective interpretation, overly parametrised simulations, and external references with known S , that solutions of the **FAAF** diradical contain a mixture of doublet and triplet species.

It is worth mentioning other advantages of using hyper‐fine‐correlated ENDOR to assign the electronic spin quantum number. The technique achieves a frequency discrimination between two multiplets which is proportional to the ratio of the magnetic quantum numbers $M_{S_{1}}$ and $M_{S_{2}}$ involved in an ENDOR transition. This is because the hyperfine field experienced by a nucleus is proportional to the magnetic quantum numbers. For doublets *vs*. triplets, this simply implies a frequency discrimination factor of 2 (illustrated by the dashed red/blue lines in Figure 7). By contrast, the nutation experiment (SI) achieves, at best, a frequency discrimination factor of $\sqrt{2}$ between doublets and triplets. Moreover, unlike nutation experiments, hyperfine‐correlated ENDOR is not subject to distortions caused by microwave field inhomogeneities or complications due to the limited excitation bandwidth of the microwave pulses. Whilst there are clear advantages, the hyperfine correlated Mims ENDOR experiment does require the presence of small hyperfine couplings and the spin‐spin relaxation time needs to be large enough relative to the hyperfine frequency. Additionally, even for systems which have good signal‐to‐noise in pulse ESR (such as these Chichibabin radicals), the hyperfine‐correlated ENDOR experiment can take a long time to run (for example, the data in each panel presented in Figure 7 were acquired over a period of 16 hours).

## Conclusions

The most important result of this work is a quantitative experimental solution to the problem of classifying the electronic spin state of organic diradicals by ESR spectroscopy. The rich ESR behaviour observed for these particular Chichi‐babin radicals, **AAF** and **FAAF**, has allowed such a solution to be developed systematically. To start with, a quantitative analysis of the liquid state cwESR spectra provided the first hint that liquid solutions of **FAAF** contain a mixture of species with different spin quantum numbers (doublets and triplets). In fact, the doublet component only appears distinctly in the ESR spectra and cannot be conclusively gleaned from the other characterisation techniques used in the synthesis (see SI). Further and clearer evidence supporting the presence of this admixture was obtained from analysing the FID spectra, the nutation spectra, and one‐dimensional ENDOR data. However, the technique which, in this case, offered the most direct discrimination between the two components proved to be hyperfine‐correlated ENDOR. This latter experiment relies on the nuclear spins acting as reporters of the electronic spin quantum numbers.

The doublet component present in solutions of **FAAF** is a fluorenyl‐based monoradical, akin to **AAF**, arising from a non‐trivial interplay of many variables, such as: interactions with molecular oxygen, with other molecules of the same type, with the solvent, and with light. However, it is now clear that the ‘doublet impurity’ noted by researchers working on these and similar systems for the past 53 years is, perhaps, not an impurity at all.[^26^, ^27^, ^28^, ^29^]

## Conflict of Interests

The authors have no conflicts of interest to declare.

1

## Supporting information

As a service to our authors and readers, this journal provides supporting information supplied by the authors. Such materials are peer reviewed and may be re‐organized for online delivery, but are not copy‐edited or typeset. Technical support issues arising from supporting information (other than missing files) should be addressed to the authors.

Supporting Information

## Data Availability

The data that support the findings of this study are available in the supplementary material of this article.
