# Supplementary material for: The Electronic Spin State of Diradicals Obtained from the Nuclear Perspective: The Strange Case of Chichibabin Radicals
Source: Chemphyschem. 2025 Jan 14;26(6):e202400707. doi: 10.1002/cphc.202400707 (PMC11913470; doi:10.1002/cphc.202400707)
Supplement: Supplementary file 1 — Supporting Information [file CPHC-26-e202400707-s001.pdf]

ChemPhysChem

Supporting Information

The Electronic Spin State of Diradicals Obtained from the Nuclear Perspective: The Strange Case of Chichibabin Radicals

Gabriel Moise,* Saleta Fernández, Kit Joll, Mikhail V. Vaganov, Fátima García, Christiane R. Timmel, Diego Peña,* and Arzhang Ardavan*

Supporting Information

The electronic spin state of diradicals obtained from the nuclear perspective: the strange case of Chichibabin radicals

Gabriel Moise,[1] Saleta Fernández,[2] Kit Joll,[1,3] Mikhail Vaganov,[1] Fátima García,[2] Christiane R. Timmel,[3] Diego Peña,[2] and Arzhang Ardavan[1]

Contents

1 Compound synthesis and characterisation 2

1.1 General methods . . . 2

1.2 Synthesis of diradicals **FAAF** and ^{13}C–**FAAF** . . . 2

1.3 Synthesis of monoradicals **AAF** and ^{13}C-**AAF** . . . 6

1.4 NMR Spectra . . . 8

2 Regarding the halving of the hyperfine couplings in FAAF *vs.* AAF 14

3 Distinguishing doublets and triplets by ESR 15

3.1 Continuous-wave ESR . . . 15

3.2 Pulse ESR and electron-nuclear double resonance . . . 16

4 Experimental ESR details 18

4.1 Sample preparation . . . 18

4.2 Countinous-wave ESR . . . 18

4.3 FID detected field swept spectra . . . 18

4.4 Electron-nuclear double resonance . . . 19

4.5 Data processing and simulations . . . 19

5 Further ESR results 20

5.1 The effects of thermal cycling . . . 20

5.2 Interactions with molecular oxygen . . . 21

5.3 The effects of light . . . 22

5.4 *In-situ* radical formation . . . 22

5.5 Spin relaxation in **FAAF** . . . 24

5.6 Solvent dependence of FID spectra . . . 27

5.7 Solvent dependence of Mims ENDOR spectra . . . 28

5.8 Characterisation of the ^{13}C hyperfine coupling in **AAF** . . . 29

5.9 Nutation experiments . . . 31

5.10 Frozen solution cwESR . . . 31

6 Density Functional Theory calculations 32

6.1 Key results . . . 32

6.2 ORCA input files and all hyperfine tensors . . . 33

References 87

You start a question, and it's like starting a stone. You sit quietly on the top of a hill; and away the stone goes, starting others...

The strange case of Dr. Jekyll and Mr. Hyde, **Robert Louis Stevenson**

[1]Centre for Advanced Electron Spin Resonance, The Clarendon Laboratory, Department of Physics, University of Oxford, Parks Road, Oxford, United Kingdom, OX1 3PU

[2]Centro Singular de Investigación en Química Biolóxica y Materiais Moleculares, C/ Jenaro de la Fuente s/n (esquina Avda. Mestre Mateo), Campus Vida, Universidade de Santiago de Compostela, Santiago de Compostela, Spain, 15782

[3]Centre for Advanced Electron Spin Resonance, Inorganic Chemistry Laboratory, University of Oxford, South Parks Road, Oxford, United Kingdom, OX1 3QR

1 Compound synthesis and characterisation

1.1 General methods

All the reactions were carried out under argon using oven-dried glassware. CH_2Cl_2 and tetrahydrofuran (THF) were dried using a MBraun SPS-800 Solvent Purification System. Finely powdered $SnCl_2$ was purchased from Sigma-Aldrich, opened and stored in a glove-box. N,N-Dimethylformamide-(carbonyl-^{13}C) 99% atom ^{13}C was purchased from Sigma-Aldrich and stored under activated 3Å molecular sieves. Other commercial reagents were purchased from ABCR GmbH, Sigma-Aldrich or Acros Organics, and were used without further purification. Deuterated solvents were purchased from Acros Organics. TLC was performed on Merck silica gel 60 F254 and chromatograms were visualized with UV light (254 and 365 nm) and/or stained with Hanessian's stain. Column chromatography was performed on Merck silica gel 60 (ASTM 230-400 mesh). ^{1}H and ^{13}C NMR spectra were recorded at 300 and 75 MHz (Varian Mercury-300 instrument) or 500 and 125 MHz (Varian Inova 500 or Bruker 500) respectively. APCI high resolution mass spectra were obtained on a Bruker Microtof.

1.2 Synthesis of diradicals FAAF and ^{13}C–FAAF

The preparation of diradicals **FAAF** and ^{13}C–**FAAF** was performed using the procedure described by Zeng et al., which is shown in Scheme S1.[1]

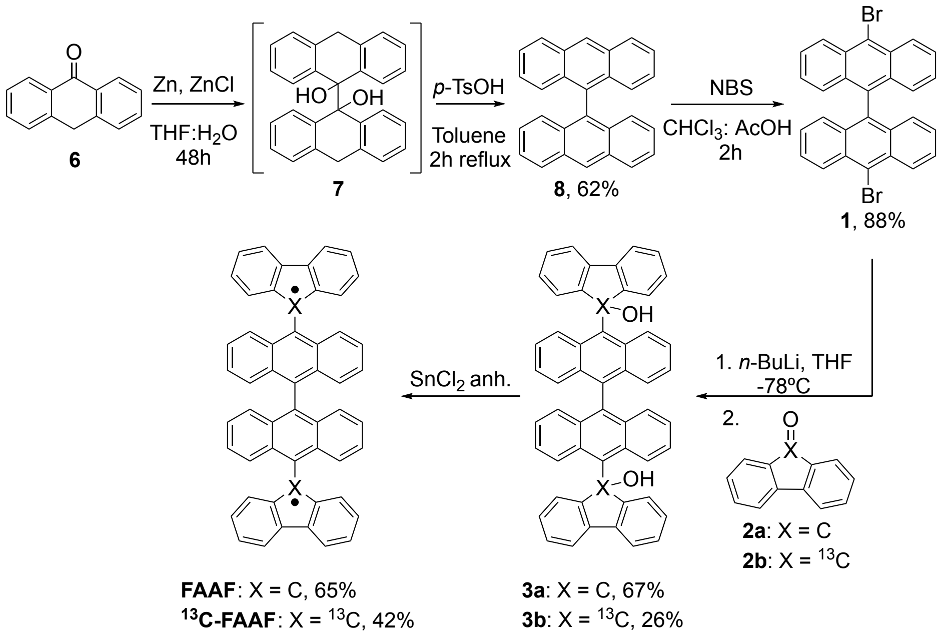

Scheme S1: Synthesis of diradicals **FAAF** and ^{13}C–**FAAF**.

The synthesis of 10-bromo-9,9'-bianthracene (**1**) was carried out using a modified procedure from the literature.[2,3]

1.2.1 Reaction of 10,10'-dibromo-9,9'-bianthracene (1) with fluorenone 2a

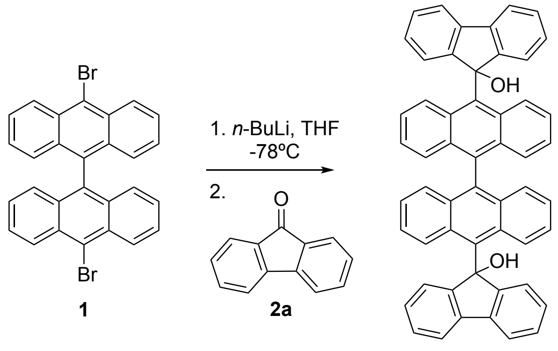

In a round bottom flask was added compound **1** (500 mg, 0.98 mmol) and dry THF (25 mL) under argon atmosphere. The solution was cooled to -78°C and *n*-BuLi solution (2.0 M in hexane, 1.1 mL, 2.15 mmol) was slowly

added. The mixture was stirred for 1 h at -78°C and then 9*H*-fluoren-9-one (**2a**, 350 mg, 1.90 mmol) solution in THF (5 mL) was added. The solution was slowly warmed to room temperature and stirred for 16h. Then water (20 mL) was added and extracted by $CHCl_3$ (3×50 mL). The organic layer was dried over anhydrous $MgSO_4$. The solvent was removed under reduced pressure and the residue was purified by column chromatography (SiO_2; hexane:CH_2Cl_2 2:1). The product obtained was washed with MeOH yielding the compound **3a** (467 mg, 67%) as a yellow solid.[1]

^{1}H–NMR (300 MHz, $CDCl_3$) δ = 9.89 (d, J = 9.3 Hz, 2H), 7.90 (d, J = 7.6 Hz, 4H), 7.49 (tdd, J = 8.7, 7.1, 4.8 Hz, 11H), 7.32-7.26 (m, 2H), 7.18 (dd, J = 3.5, 0.9 Hz, 4H), 7.12 (d, J = 9.1 Hz, 3H), 7.02-6.97 (m, 2H), 6.85 (d, J = 1.4 Hz, 2H), 6.76-6.69 (m, 2H), 2.79 (s, 2H) ppm.

1.2.2 Generation of FAAF

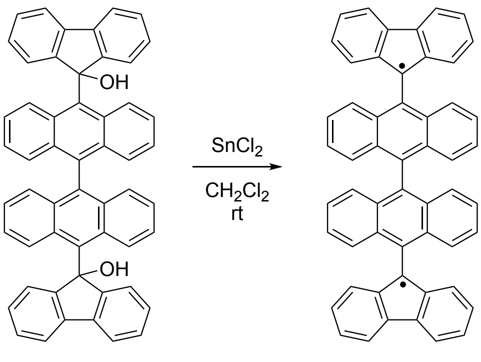

3a **FAAF**, 65%

Under argon atmosphere, a solution of **3a** (105 mg, 0.147 mmol) in dry DCM (30 mL) was added to $SnCl_2$ (139 mg, 0.735 mmol). The mixture was stirred 16h at room temperature. The solvent was removed under reduced pressure. The residue was then purified by column chromatography (SiO_2; hexane:$CHCl_3$ 20:1) yielding compound **FAAF** (65 mg, 65%) as a red solid.

HRMS (APCI) calculated for $[M+2H]^+$ $C_{54}H_{32}$: 682.2655, found 682.2651 (error: 0.6 ppm).

1.2.3 Synthesis of 9*H*-fluoren-9-one-9-^{13}C (2b)

The introduction of a labelled carbon in the structure of **FAAF** was possible by using fluorenone **2b** which was prepared following the synthetic route shown in Scheme 2.

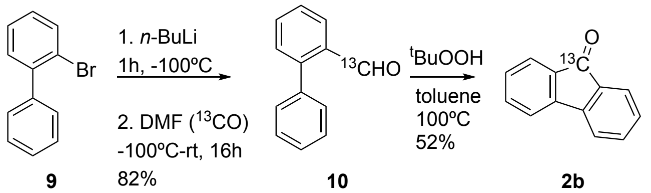

9 **10** **2b**

Scheme S2: Synthesis of 9*H*-fluoren-9-one-9-^{13}C (**2b**)

1.2.4 Synthesis of [1,1'-biphenyl]-2-carbaldehyde-^{13}C (10)

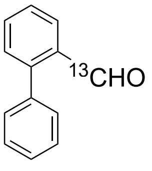

10

Figure S1: Structure of [1,1'-biphenyl]-2-carbaldehyde-^{13}C (**10**)

In a 25 mL dry round bottom flask under Ar atmosphere, 2-bromobiphenyl (**9**) (0.371 mL, 2.15 mmol) was placed and dissolved in dry THF (5 mL). The solution was then cooled to -100°C and a *n*-BuLi (2.2 M, 0.640 mL, 1.41

mmol) was added dropwise. The solution was stirred at -100°C for 1 hour and then, *N,N*-dimethylformamide-(carbonyl-^{13}C) (0.500 mL, 6.46 mmol) was added dropwise. The reaction mixture was allowed to reach room temperature. Then, the reaction was diluted with EtOAc (5 mL) and quenched with cold water (10 mL). After that, the organic layer was separated in a separation funnel and the organic layer was washed with NH_4Cl 5% solution (3×10 mL) and NaCl sat. solution (1×10 mL). The organic layer was dried with $MgSO_4$, filtered and the solvent removed under reduced pressure. The residue was purified by column chromatography (silica gel, gradient hexane-hexane/EtOAc 20/1) yielding **10** as a colourless liquid (323 mg). Yield: 82%.
^{1}H-NMR (300 MHz, $CDCl_3$) δ = 10.38-9.61 (d, 179 Hz, 1H, ^{13}CHO), 8.05 (ddd, J = 7.8, 4.0, 1.5 Hz, 1H), 7.66 (td, J = 7.5, 1.5 Hz, 1H), 7.57-7.37 (m, 6H) ppm.
^{13}C-NMR (126 MHz, $CDCl_3$) δ = 192.42 (^{13}CHO), 150.08 (CH Ar), 146.33 (C Ar, d, J = 7.8 Hz), 138.12 (C Ar), 134.28 (C Ar), 133.87 (CH Ar), 131.10 (CH Ar, d, J = 3.5 Hz), 130.43 (CH Ar), 128.44 (CH Ar), 128.12 (CH Ar), 128.09 (CH Ar), 127.90 (CH Ar) ppm.
HRMS (APCI) found for $[M+1]^+$, $C_{12}H_{11}O^{13}C$: 184.0838, calculated: 184.0837 (error: 0.6 ppm).

1.2.5 Synthesis of 9*H*-fluoren-9-one-9-^{13}C (2b)

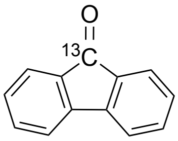

2b

Figure S2: Structure of 9*H*-fluoren-9-one-9-^{13}C (**2b**)

[1,1'-Biphenyl]-2-carbaldehyde-^{13}C (**10**) (191 mg, 1.044 mmol) was dissolved in dry toluene (5 mL) and then, *tert*-butyl hydroperoxide was added (nonane 5 M solution, 0.63 mL, 3.131 mmol). The solution was heated to reflux for 24 hours, when the reaction was monitored by TLC, revealing unreacted biphenyl carbaldehyde. Therefore, extra *tert*-butyl hydroperoxide was added (nonane 5 M solution, 0.63 mL, 3.131 mmol) and the reaction heated again for another 24 hours. Then, the reaction was diluted with diethyl ether (5 mL), cooled to 0°C, quenched with 5 % $Na_2S_2O_5$ solution (10 mL) and stirred for 15 minutes. The organic layer was separated and washed with 5 % $Na_2S_2O_5$ solution (2×10 mL), saturated Na_2CO_3 (3×10 mL) and brine (1×10 mL). The organic layer was dried with $MgSO_4$, filtered and the solvent removed under reduced pressure. The residue was purified by column chromatography (silica gel, hexane/diethyl ether 10/1) yielding **2b** as a yellow solid (98 mg). Yield: 52%.
^{1}H-NMR (300 MHz, $CDCl_3$) δ = 7.61 (dd, J = 7.3, 3.1 Hz, 2H), 7.48-7.37 (m, 4H), 7.24 (m, 2H) ppm.
^{13}C-NMR (75 MHz, $CDCl_3$) δ = 194.16 (C=O), 144.68 (C Ar, d, J = 8.1 Hz), 134.90 (CH Ar), 134.33 (C Ar, d, J = 55.3 Hz), 129.28 (d, J = 3.7 Hz), 124.47 (d, J = 2.6 Hz), 120.54 (d, J = 3.7 Hz) ppm.
HRMS (APCI) found for $[M+1]^+$, $C_{12}{}^{13}CH_8O$: 182.0681, calculated: 182.0681 (error: 0.5 ppm).

1.2.6 Synthesis of the diol 3b

The synthesis of the diol **3b** was carried out using a modified procedure from literature.[1]

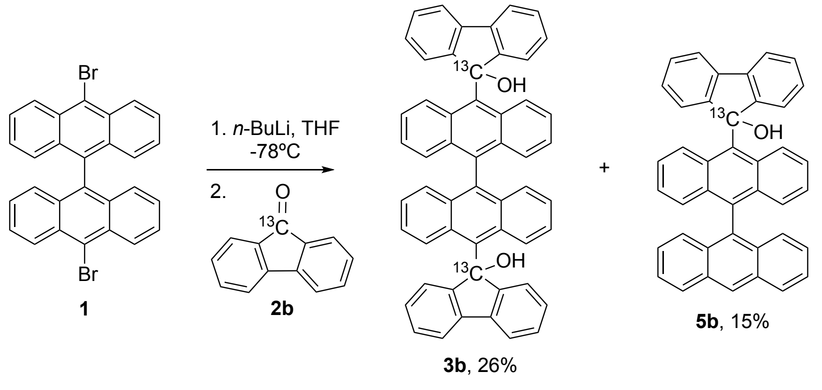

10,10'-Dibromo-9,9'-bianthracene (**1**) (132 mg, 0.258 mmol) was dissolved in dry THF (5 mL) under argon atmosphere. Then, the solution was placed at -100°C and a solution of *n*-butyllithium in hexanes (2.27 M, 0.25 mL, 0.567 mmol) was added dropwise and stirred at -100°C for 1 hour. Then, a solution of 9*H*-fluorenone-^{13}C (**2b**) (98 mg, 0.54 mmol) in dry THF solution (2 mL) was added dropwise. The reaction mixture was allowed to reach room

temperature in the cooling bath and stirred for 24 hours. Then, the reaction was quenched with water (20 mL) and extracted with $CHCl_3$ (3×20 mL) and the combined organic layers washed with brine (1×10 mL). The organic layer was dried with $MgSO_4$, filtered and the solvent removed under reduced pressure. The residue was purified by column chromatography (SiO_2; gradient hexane:CH_2Cl_2 8:2 - 0:10) yielding 48 mg of diol **3b** (26% yield) and 21 mg of alcohol **5b** (15% yield) and as dark yellow solids.

Diol 3b:
^{1}H-NMR (300 MHz, $CDCl_3$) δ = 9.91 (d, J = 9.4 Hz, 2H), 7.91 (d, J = 7.5 Hz, 4H), 7.50 (ddt, J = 15.1, 12.2, 5.0 Hz, 10H), 7.36-7.09 (m, 10H), 7.02 (d, J = 8.4 Hz, 2H), 6.86 (dd, J = 9.0, 5.9 Hz, 2H), 6.80-6.68 (m, 2H), 2.82 (s, 2H) ppm.
^{13}C-NMR (75 MHz, $CDCl_3$) δ = 152.83 (C Ar, d, J = 47.3 Hz), 139.19 (C Ar, d, J = 5.6 Hz), 135.92 (C Ar), 132.15 (C Ar), 131.73 (C Ar, d), 129.62 (CH Ar), 129.29 (CH Ar), 128.08 (CH Ar), 127.56 (CH Ar), 126.50 (CH Ar), 125.35 (CH Ar), 125.00 (CH Ar), 124.76 (CH Ar), 124.20 (CH Ar), 121.39 (CH Ar, d), 87.58 (C-OH) ppm.
HRMS (APCI) found for $[M-17]^+$, $C_{52}{}^{13}C_2H_{33}O$: 699.2599, calculated: 699.2593 (error 0.9 ppm).

Alcohol 5b:
^{1}H-NMR (500 MHz, $CDCl_3$) δ = 9.90 (d, J = 9.3 Hz, 1H), 8.68 (s, 1H), 8.15 (d, J = 8.5 Hz, 2H), 7.91 (d, J = 7.5 Hz, 2H), 7.55-7.42 (m, 7H), 7.28 (d, J = 7.5 Hz, 2H), 7.25-7.08 (m, 7H), 6.99 (d, J = 8.8 Hz, 1H), 6.85-6.78 (m, 1H), 6.76-6.69 (m, 1H), 2.81 (OH, 1H) ppm.
^{13}C-NMR (126 MHz, $CDCl_3$) δ = 152.65 (d, J = 47.2 Hz), 139.00 (d, J = 5.9 Hz), 135.17 (C Ar), 134.23 (C Ar), 133.82 (C Ar), 133.79 (C Ar), 132.72 (C Ar), 132.69 (C Ar), 131.95 (C Ar), 131.74 (d, J = 15.1 Hz), 131.50 (C Ar), 131.47 (C Ar), 129.42 (CH Ar), 129.07 (CH Ar), 129.05 (CH Ar), 128.67 (CH Ar), 128.52 (C Ar), 128.49 (C Ar), 127.75 (CH Ar), 127.35 (CH Ar), 127.23 (CH Ar), 127.10 (CH Ar), 126.31 (CH Ar), 126.29 (CH Ar), 126.01 (CH Ar), 125.49 (CH Ar), 125.15 (CH Ar), 125.13 (CH Ar), 125.12 (CH Ar), 124.74 (CH Ar), 124.53 (CH Ar), 123.97 (CH Ar), 121.22 (CH Ar), 121.20 (CH Ar), 87.40 (C-OH) ppm.
HRMS (APCI) found for $[M]^+$, $C_{40}{}^{13}CH_{26}O$: 535.2023, calculated: 535.2012 (error: 2.1 ppm).

1.2.7 Generation of ^{13}C–FAAF

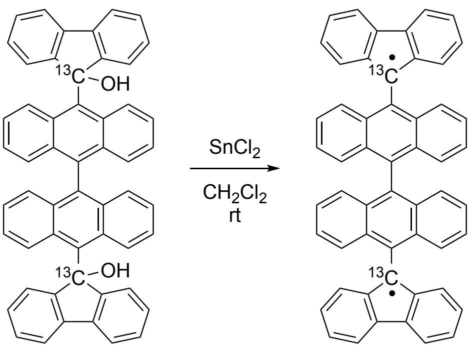

3b **^{13}C-FAAF**, 42%

Diol **3b** (20 mg, 0.028 mmol) and anhydrous $SnCl_2$ (29 mg, 0.140 mmol) were placed in a Schlenk tube and dissolved in dry and degassed DCM (6 mL) under argon atmosphere. The reaction mixture turned immediately dark purple and it was stirred 16h at room temperature. The crude was purified by column chromatography under Ar atmosphere (silica gel, hexane:CH_2Cl_2 6:4). Then, the solvent was evaporated under reduced pressure (Schlenk line) yielding 8 mg of the diradical as a dark purple solid. Yield: 42%.
HRMS (APCI) found for $[M+2]^+$, $C_{52}{}^{13}C_2H_{32}$: 684.2702, calculated: 684.2722 (error 2.4 ppm).

1.3 Synthesis of monoradicals AAF and ^{13}C-AAF

For the generation of monoradical **AAF** a similar approach was developed (Scheme 3). The synthesis of 10-bromo-9,9'-bianthracene (**4**) was carried out following a reported procedure.[2,3]

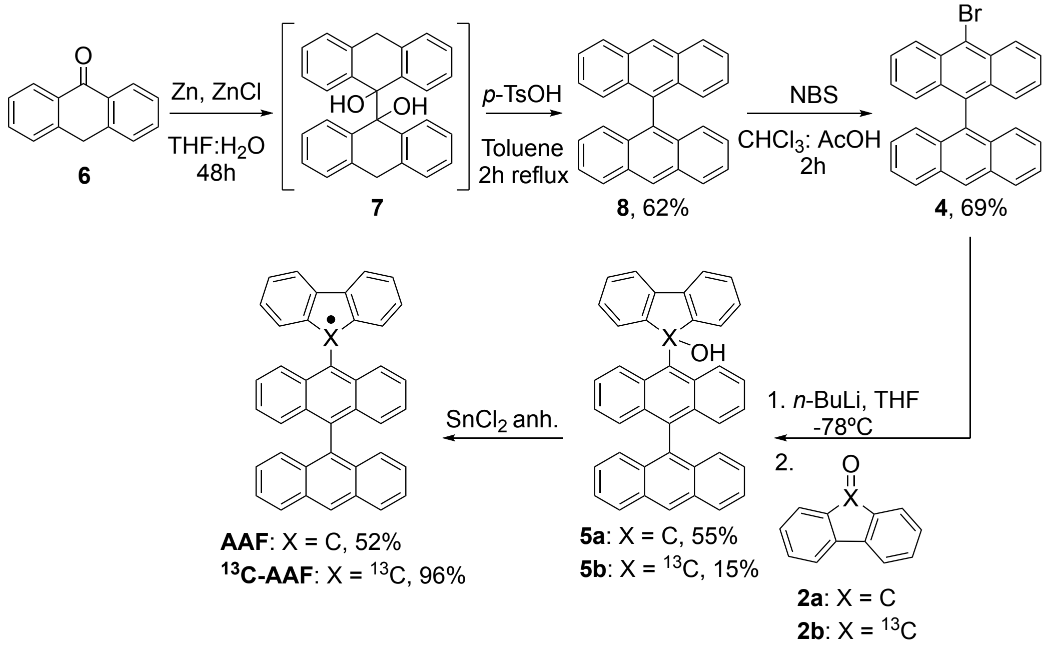

Scheme S3: Synthesis of monoradicals **AAF** and ^{13}C-**AAF**

1.3.1 Reaction of 10-bromo-9,9'-bianthracene (4) with fluorenone 2a

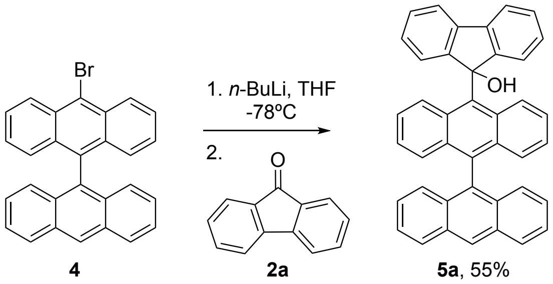

In a round bottom flask was added compound **4** (500 mg, 0.98 mmol) and dry THF (5 mL) under argon atmosphere. The solution was cooled to -78°C and *n*-BuLi solution (2.0 M in hexane, 1.1 mL, 2.15 mmol) was slowly added. The mixture was stirred for 1 h at -78°C and then 9*H*-fluoren-9-one (**2a**, 350 mg, 1.90 mmol) in anhydrous THF solution (5 mL) was added. The solution was slowly warmed to room temperature and stirred for 16h. Then water (20 mL) was added and extracted by $CHCl_3$ (3×50 mL). The organic layer was dried over anhydrous $MgSO_4$. The solvent was removed under vacuum and the residue was purified by column chromatography (SiO_2; hexane: CH_2Cl_2 2:1) yielding alcohol **5a** (343 mg, 55%) as a yellow solid.[4]

^{1}H-NMR (300 MHz, $CDCl_3$) δ = 9.90 (d, J = 9.3 Hz, 1H), 8.68 (s, 1H), 8.15 (d, J = 8.5 Hz, 2H), 7.91 (d, J = 7.5 Hz, 2H), 7.55-7.40 (m, 8H), 7.32-7.26 (m, 1H), 7.23-7.07 (m, 7H), 6.98 (d, J = 8.8 Hz, 1H), 6.84-6.79 (m, 1H), 6.75-6.70 (m, 1H), 2.79 (s, 1H) ppm.

^{13}C-NMR (126 MHz, $CDCl_3$) δ = 151.49 (C), 137.84 (C), 134.01 (C), 132.87 (C), 132.63 (C), 131.54(C), 130.78 (C), 130.64 (C), 130.52 (C), 130.32 (C), 128.26 (CH) 127.90 (CH), 127.51 (C), 127.34 (CH), 126.58 (CH), 126.18 (CH), 126.07 (CH), 125.94 (CH), 125.14 (CH), 124.84 (CH), 124.32 (CH), 123.98 (CH), 123.58 (CH), 123.37 (CH), 122.81 (CH), 120.05 (CH), 86.24 (C-OH) ppm.

HRMS (APCI) found for [M+], $C_{41}H_{26}O$: 534.1975, calculated 534.1978 (error: 0.3 ppm)

1.3.2 Generation of AAF

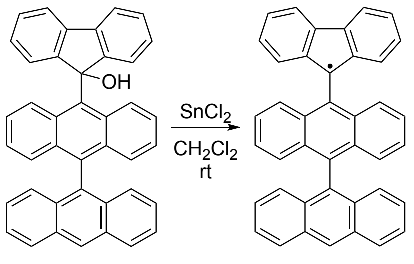

Under an argon atmosphere, a solution of alcohol **5a** (150 mg, 0.28 mmol) in dry DCM (6 mL) was added to $SnCl_2$ (531 mg, 2.8 mmol). The mixture was stirred 16h at room temperature. The solvent was removed under reduced pressure. The residue was then purified by flash chromatography at 0°C (SiO_2; hexane:CH_2Cl_2 3:1) yielding **AAF** (75 mg, 52%) as a red solid.[4]

HRMS (APCI) found for $[M+1]^+$, $C_{41}H_{26}$: 518.2028, calculated: 518.2029 (error: 0.1 ppm)

1.3.3 Generation of ^{13}C-AAF

Alcohol **5b** was obtained as a subproduct of the reaction of 10,10'-dibromo-9,9'-bianthracene (**4**) with fluorenone **2b**.

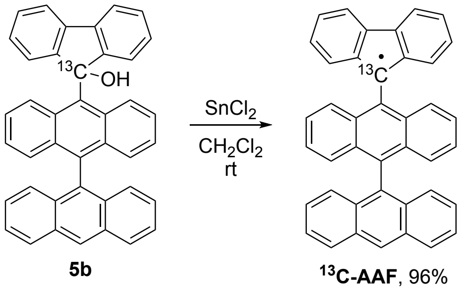

Alcohol **5b** (18 mg, 0.034 mmol) and anhydrous $SnCl_2$ (17 mg, 0.084 mmol) were placed in a Schlenk tube and dissolved in dry and degassed DCM (4 mL) under argon atmosphere and kept covered from light. The reaction mixture turned immediately dark purple, and it was stirred 1h at room temperature. The crude was filtered through Celite under Ar atmosphere. Then, the solvent was evaporated under reduced pressure (Schlenk line) yielding 17 mg of the radical as a dark purple solid. Yield: 96%.

HRMS (APCI) found for $[M+1]^+$, $C_{40}{}^{13}C_1H_{26}$: 519.2061, calculated: 519.2063 (error: 0.4 ppm).

1.4 NMR Spectra

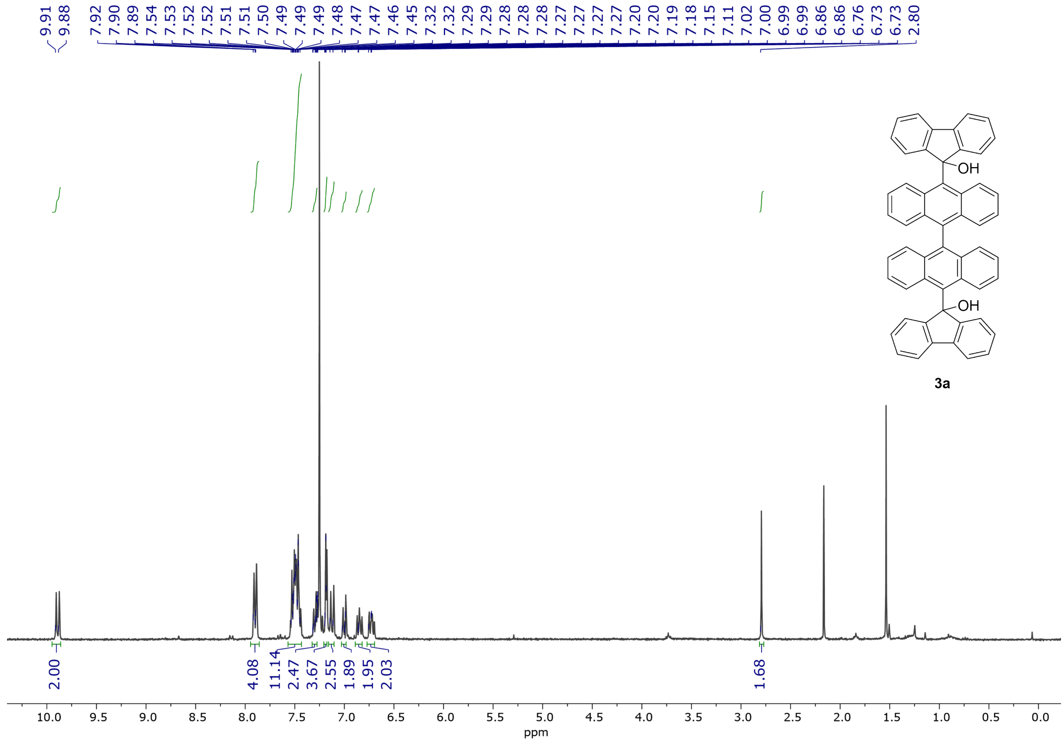

Figure S3: ^{1}H-NMR (300 MHz, $CDCl_3$) of diol **3a**.

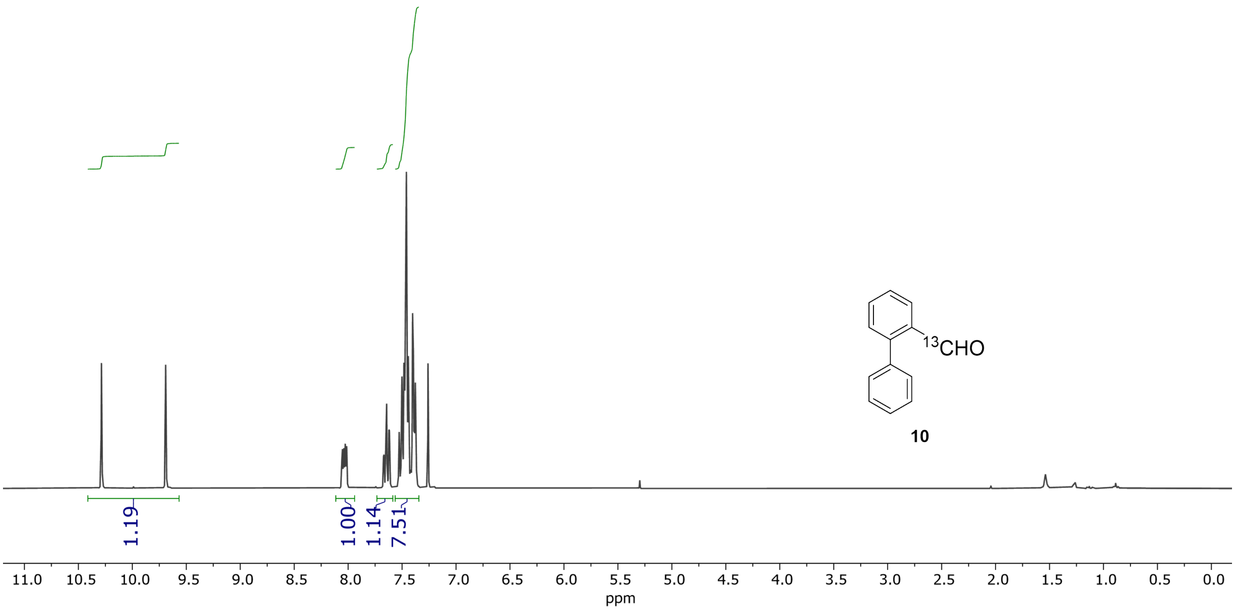

Figure S4: ^{1}H-NMR (300 MHz, $CDCl_3$) of [1,1'-biphenyl]-2-carbaldehyde-^{13}C (**10**).

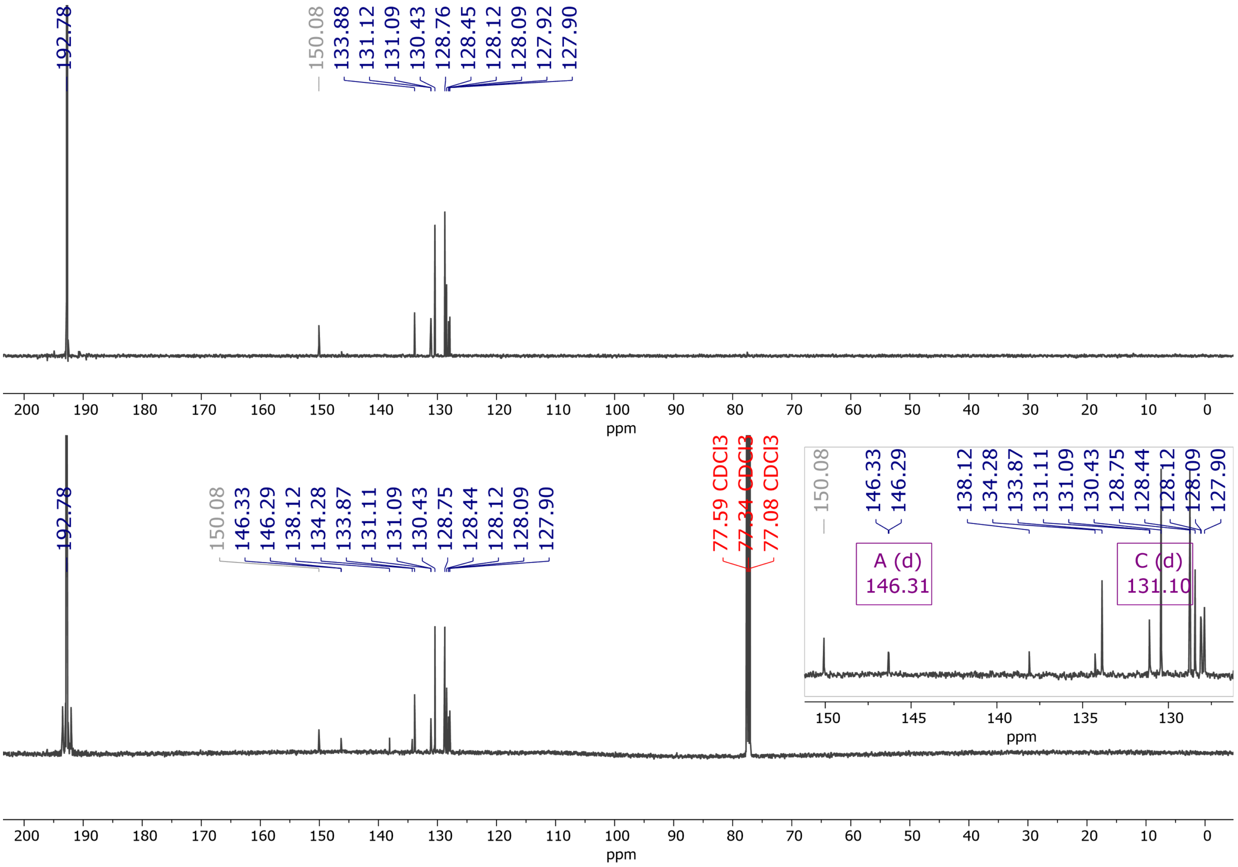

Figure S5: DEPT135 (top) and ^{13}C-NMR (bottom) (126 MHz, $CDCl_3$) of [1,1'-biphenyl]-2-carbaldehyde-^{13}C (**10**)

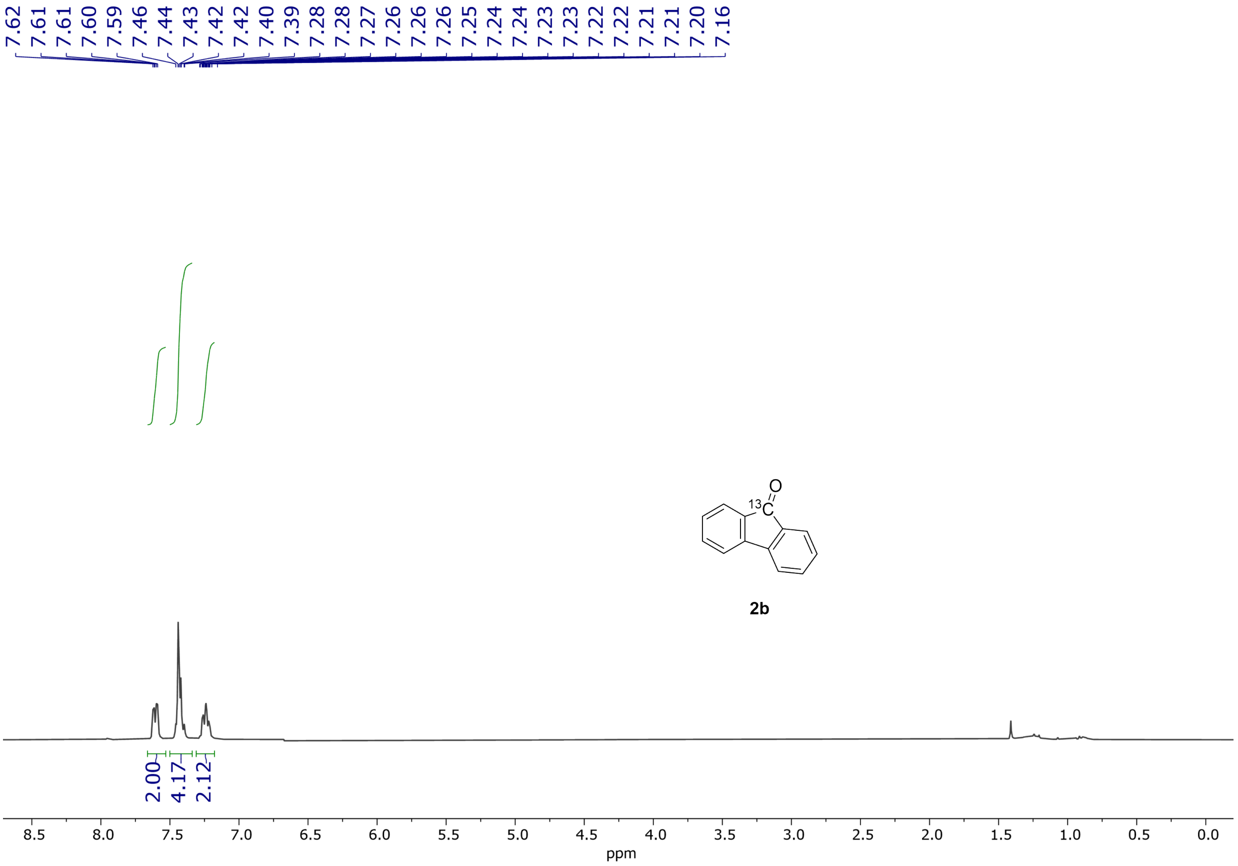

Figure S6: ^{1}H-NMR (300 MHz, $CDCl_3$) of 9*H*-fluoren-9-one-9-^{13}C (**2b**).

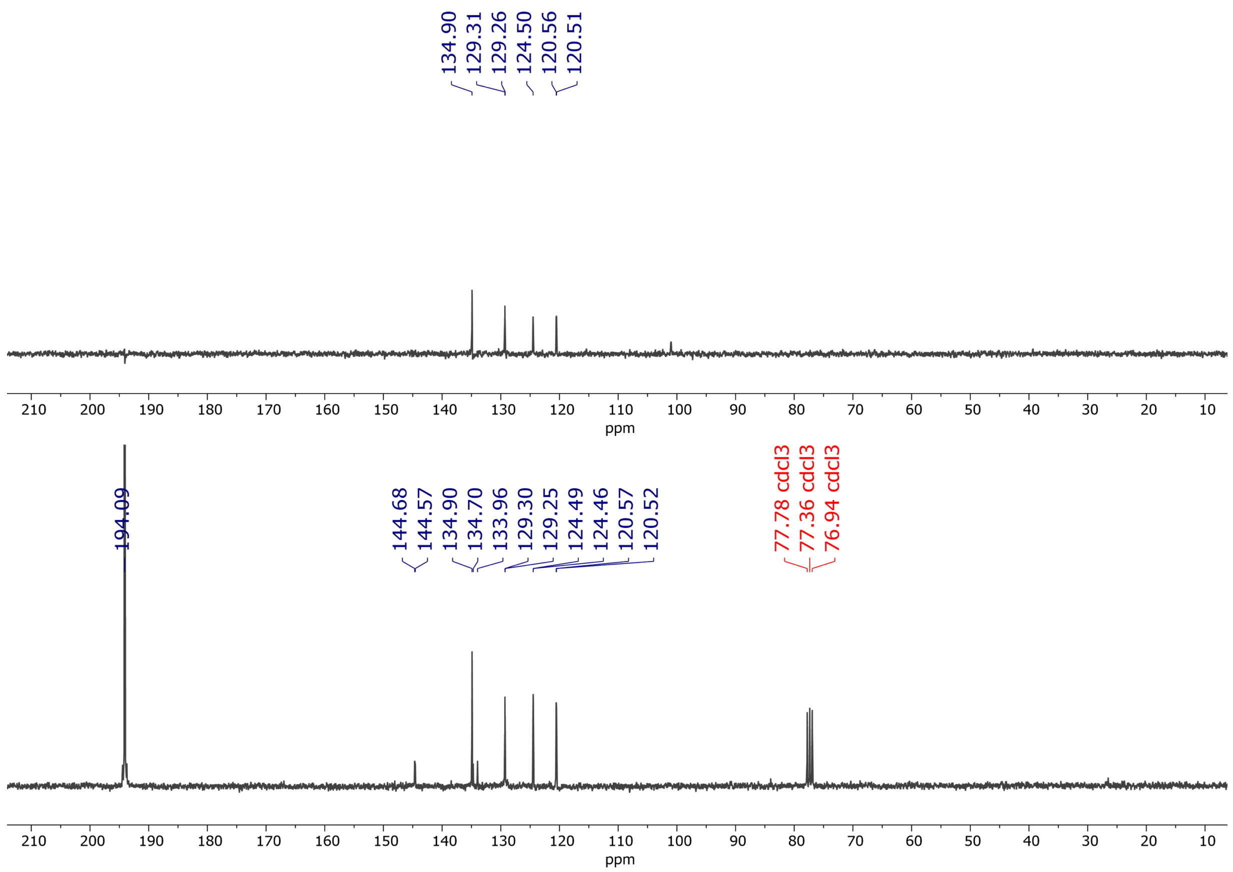

Figure S7: DEPT135 (top) and ^{13}C-NMR (bottom) (126 MHz, $CDCl_3$) of 9*H*-fluoren-9-one-9-^{13}C (**2b**)

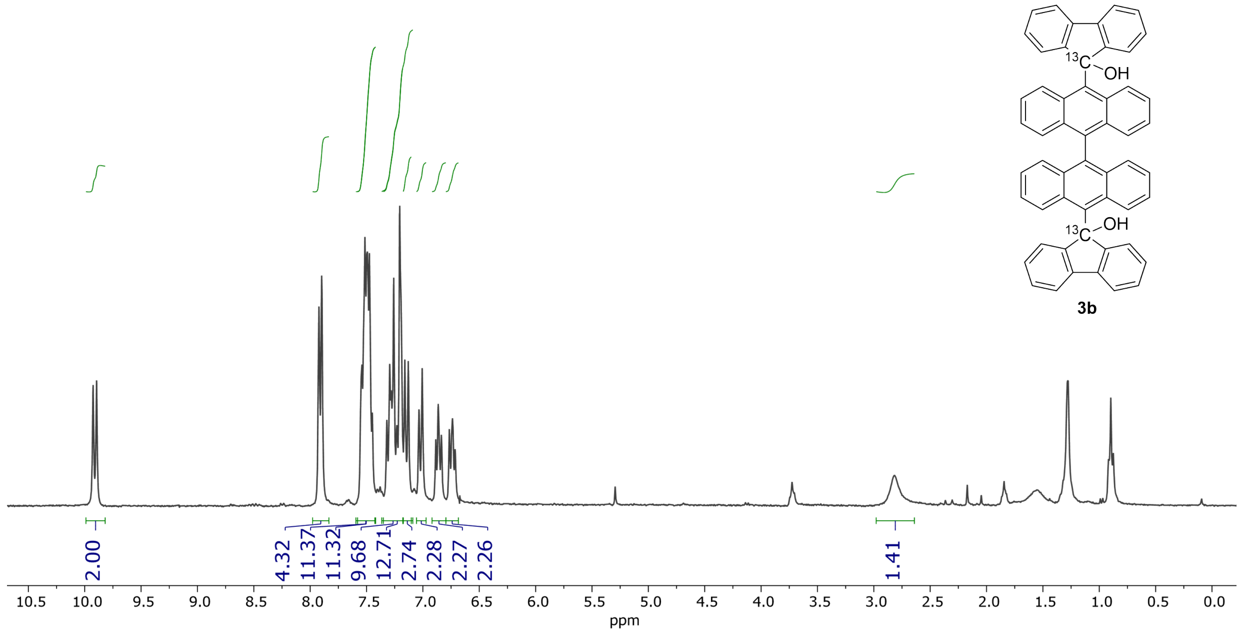

Figure S8: ^{1}H-NMR (300 MHz, $CDCl_3$) of diol **3b**.

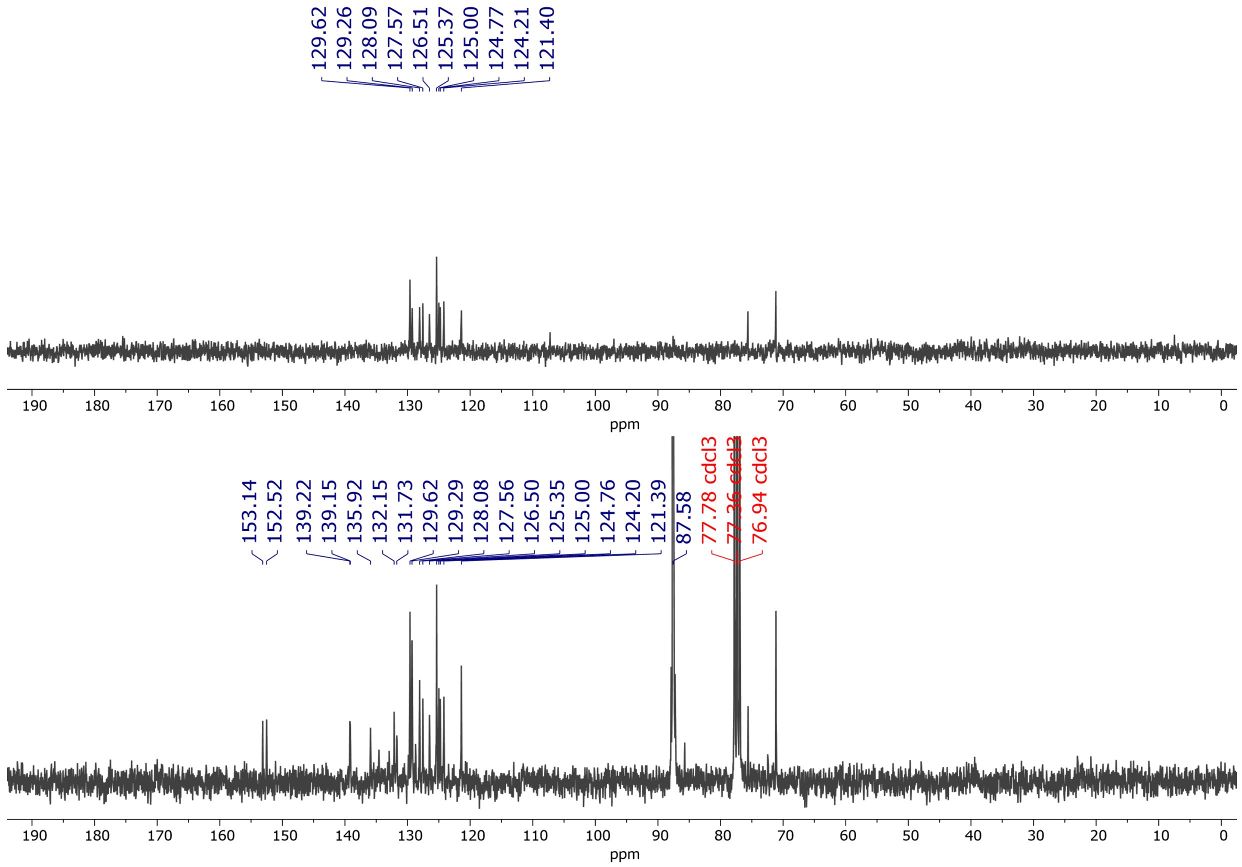

Figure S9: DEPT135 (top) and ^{13}C-NMR (bottom) (75 MHz, $CDCl_3$) of diol **3b**.

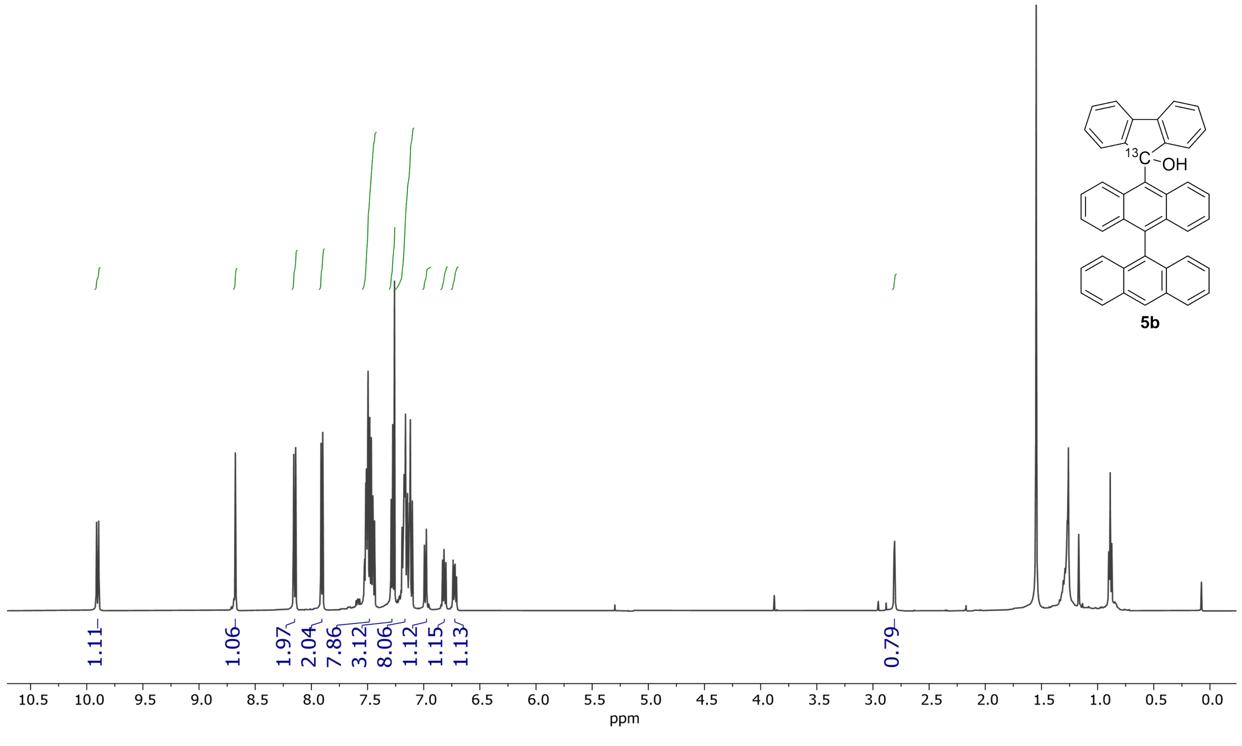

Figure S10: ^{1}H-NMR (500 MHz, $CDCl_3$) of alcohol **5b**.

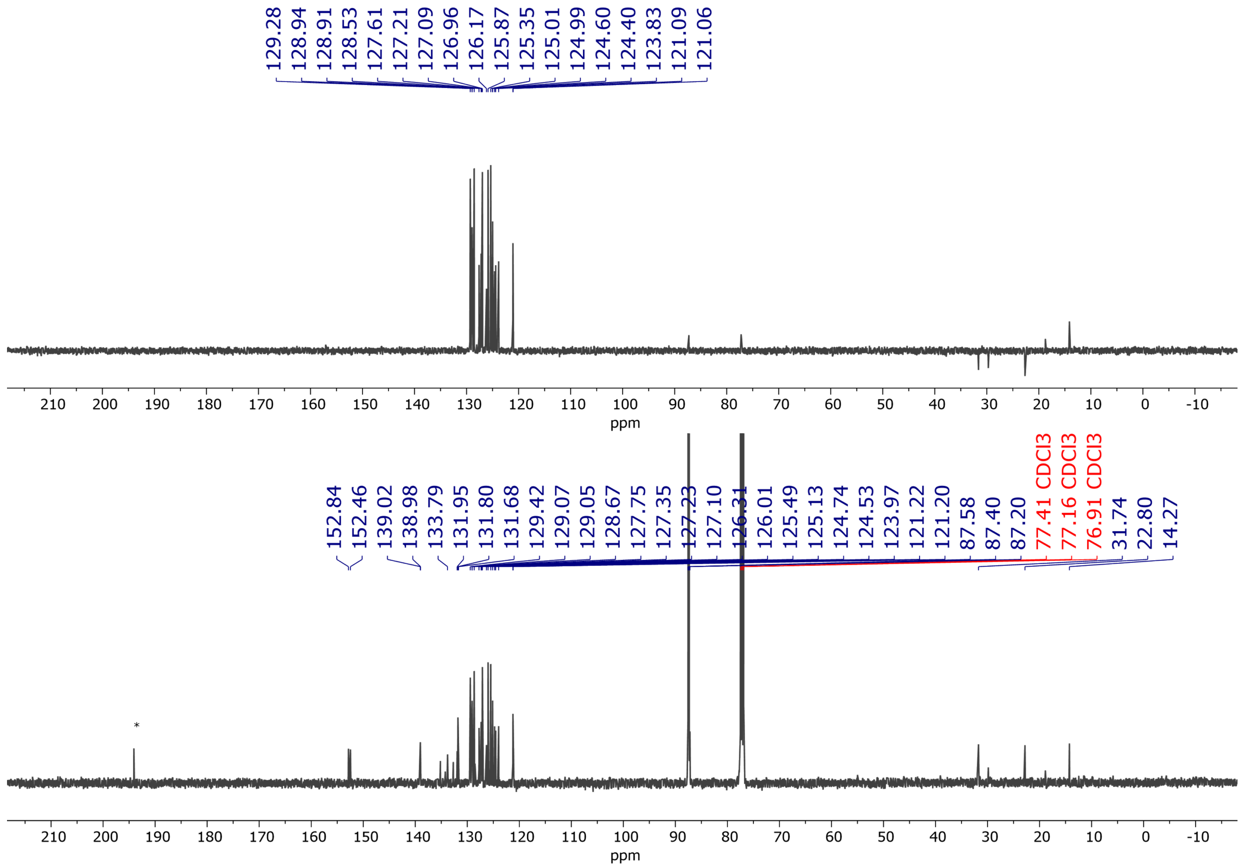

Figure S11: DEPT135 (top) and ^{13}C-NMR (bottom) (126 MHz, $CDCl_3$) of alcohol **5b**. * Signal which corresponds to unreacted ^{13}C-fluorenone.

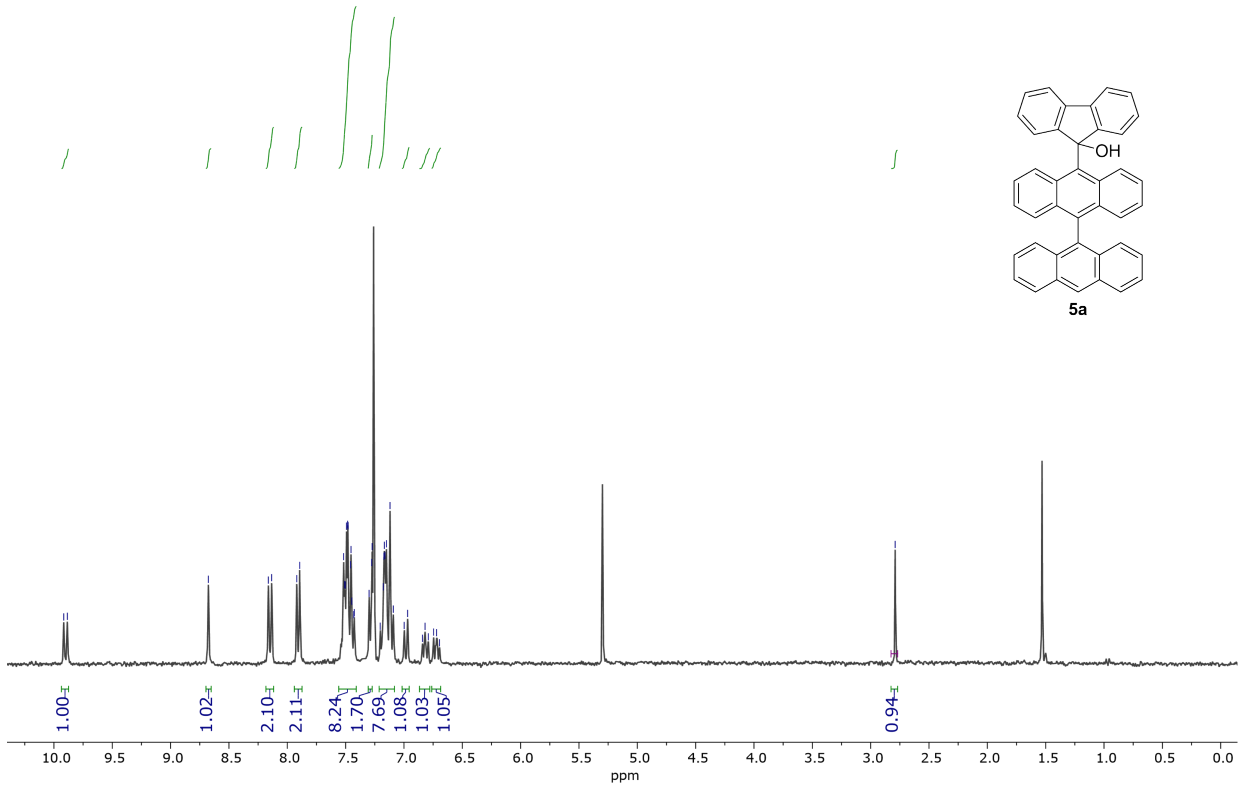

Figure S12: ^{1}H-NMR (300 MHz, $CDCl_3$) of alcohol **5a**.

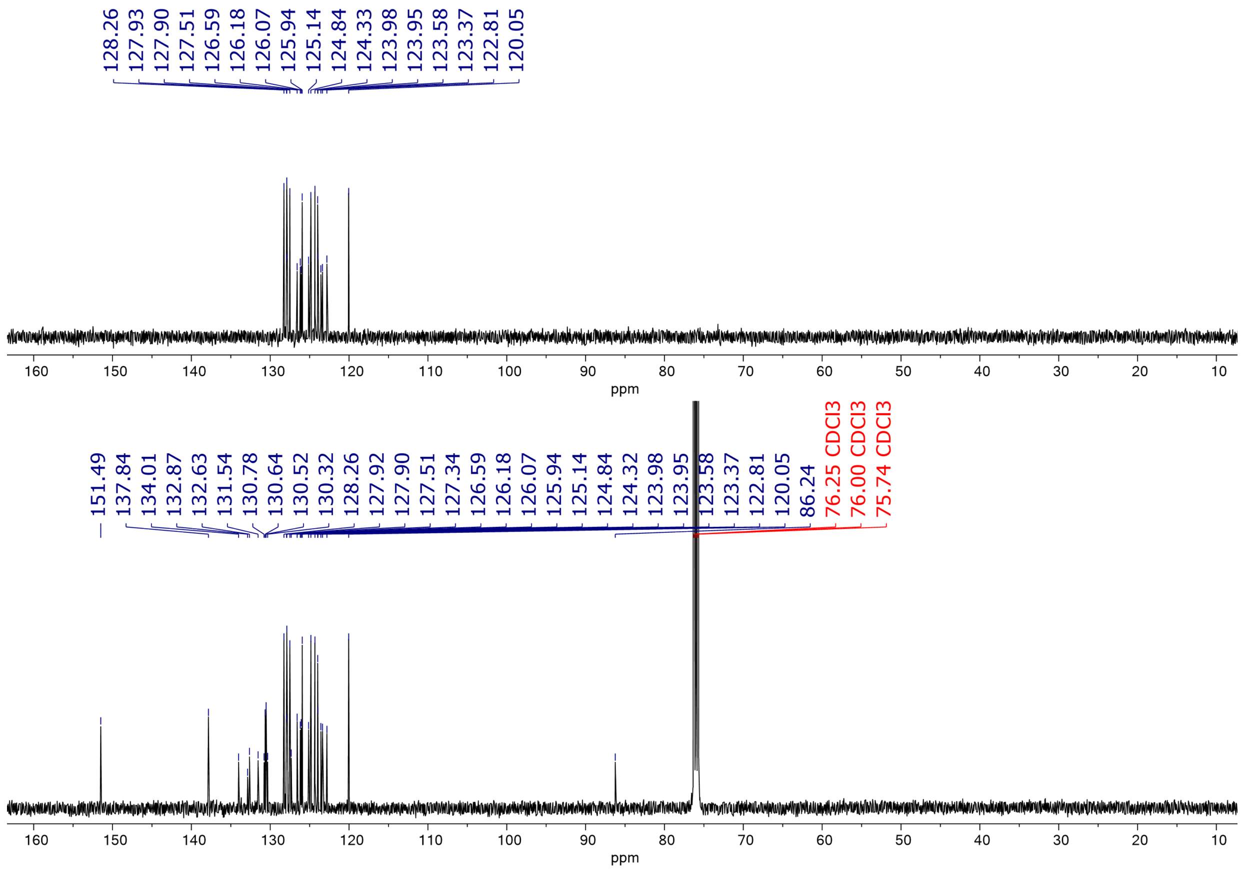

Figure S13: DEPT135 (top) and ^{13}C-NMR (bottom) (126 MHz, $CDCl_3$) of alcohol **5a**.

2 Regarding the halving of the hyperfine couplings in FAAF *vs.* AAF

The purpose of this section is to justify the observed halving of the proton/carbon hyperfine couplings in **FAAF** relative to **AAF** by employing a simple 'toy model' for an $S = 1/2$ monomer (such as **AAF**) and an $S = 1$ dimer (such as **FAAF**). The results presented in the main text for the **FAAF** system were analysed by comparison with complementary data obtained for the **AAF** system. This latter molecule provides a useful benchmark for the diradical system because its spin quantum number is unquestionably 1/2, *i.e.* a doublet ground state. For the purposes of this discussion, **AAF** will be regarded as a covalently dimerised version of **FAAF**, *as drawn*. In other words, the spin density delocalises only minimally onto the terminal anthryl fragment of **AAF**, such that one may safely write: **AAF**≈ **AF**.

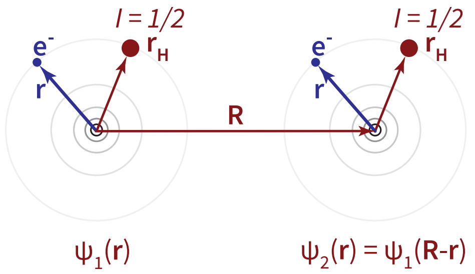

Figure S14: A simple model system used to interpret the hyperfine coupling to an arbitrary proton in an $S = 1$ dimer constructed from monomers with a single unpaired electron each ($S = 1/2$). The wavefunction of the dimer is a superposition of two monomer wavefunctions with a relative displacement **R**.

The spatial part of the wavefunction of the unpaired electron in **AAF** will be denoted as $\psi(\mathbf{r})$ where **r** is the position of the unpaired electron. The Fermi-Contact contribution to the hyperfine coupling between the electron and a proton (spin $I = 1/2$) situated at $\mathbf{r}_H$ will be proportional to the matrix element of the 3-dimensional Dirac delta function $\delta^3(\mathbf{r} - \mathbf{r}_H)$. By neglecting all proportionality constants, one may write:

$$a = \langle \psi(\mathbf{r}) | \delta^3(\mathbf{r} - \mathbf{r}_H) | \psi(\mathbf{r}) \rangle = |\psi(\mathbf{r}_H)|^2 \tag{1}$$

and within the spin Hamiltonian formalism, the hyperfine interaction term will simply be proportional to $a\hat{\mathbf{s}} \cdot \hat{\mathbf{I}}$. As mentioned, the **FAAF** molecule will be modelled as a dimerised version of the **AAF** molecule, *i.e.*

$$\mathbf{FAAF} \approx (\mathbf{FAA})_1 + (\mathbf{AAF})_2 \tag{2}$$

where 1 and 2 label the two identical fragments of the molecule.
Therefore, the natural choice of one-electron basis functions is:

$$\begin{cases} \psi_1(\mathbf{r}) = \psi(\mathbf{r}) \\ \psi_2(\mathbf{r}) = \psi(\mathbf{r} - \mathbf{R}) \end{cases} \tag{3}$$

where **R** is the relative displacement between the two fragments, and each basis function is assumed to be real (figure S14). These basis functions are not orthogonal:

$$\langle \psi_1(\mathbf{r}) | \psi_2(\mathbf{r}) \rangle = \zeta \sim \exp(-\alpha R) \,. \tag{4}$$

The overlap integral introduced above, ζ, is assumed to scale exponentially with the distance between the fragments. Consider now the hyperfine coupling to two equivalent protons: (1) the first proton, $\mathrm{H}^{(1)}$, is located at position $\mathbf{r}_H$ relative to fragment 1 and position $\mathbf{r}_H - \mathbf{R}$ relative to fragment 2, (2) the second proton, $\mathrm{H}^{(2)}$, is located at position $\mathbf{r} = \mathbf{r}_H + \mathbf{R}$ relative to fragment 1. The values of the two basis functions at the positions of these two equivalent protons will be approximated, up to an irrelevant phase factor, as follows:

$$\begin{cases} \psi_1(\mathbf{r}_H) = \sqrt{a} \\ \psi_2(\mathbf{r}_H) \sim \zeta \sqrt{a} \\ \psi_1(\mathbf{r}_H + \mathbf{R}) \sim \zeta \sqrt{a} \\ \psi_2(\mathbf{r}_H + \mathbf{R}) = \sqrt{a} \end{cases} \tag{5}$$

The interpretation of the second and third equations above is as follows: given that ψ_1 has some non-zero value ($\sqrt{a}$), the value of ψ_2 at the same point will be exponentially smaller. For the purposes of developing a simple model, the exponential scaling factor was arbitrarily chosen to be the overlap integral.

By solving the secular equations using the basis functions defined in equation 3, the one-electron wavefunctions for **FAAF** will take the following forms:

$$\psi_{\pm}(\mathbf{r}) = \frac{1}{\sqrt{2(1 \pm \zeta)}} \left[\psi_1(\mathbf{r}) \pm \psi_2(\mathbf{r})\right] \tag{6}$$

The spatial part of the two-electron wavefunction for the triplet states, $\psi_T(\mathbf{r}_1, \mathbf{r}_2)$, may therefore be approximated as:

$$\psi_T(\mathbf{r}_1, \mathbf{r}_2) = \frac{1}{\sqrt{2}} \left[\psi_+(\mathbf{r}_1)\psi_-(\mathbf{r}_2) - \psi_-(\mathbf{r}_1)\psi_+(\mathbf{r}_2)\right] \tag{7}$$

such that the total spatial-spin wavefunctions are $|\psi_T\rangle \otimes |T_{0,\pm}\rangle$.
In light of this, using equations 5, the hyperfine coupling between electron 1 and $H^{(1)}$ (located at $\mathbf{r}_H$) may be estimated to first order in perturbation theory as:

$$a' = \langle\psi_T(\mathbf{r}_1, \mathbf{r}_2)|\delta^3(\mathbf{r}_1 - \mathbf{r}_H)|\psi_T(\mathbf{r}_1, \mathbf{r}_2)\rangle = \tag{8}$$

$$= \iint d^3\mathbf{r}_1\, d^3\mathbf{r}_2\, |\psi_T(\mathbf{r}_1, \mathbf{r}_2)|^2\, \delta^3(\mathbf{r}_1 - \mathbf{r}_H) = \tag{9}$$

$$= \int d^3\mathbf{r}\, |\psi_T(\mathbf{r}_H, \mathbf{r})|^2 \sim \frac{a}{2} \tag{10}$$

Clearly, the same result is obtained for the hyperfine coupling between electron 2 and $H^{(1)}$. By symmetry, the coupling between either electron and the other proton, $H^{(2)}$, is also equal to a'. Hence, the spin Hamiltonian for the hyperfine interaction to nucleus $H^{(1)}$ may be written for **FAAF** as follows:

$$\hat{H}_{\mathrm{hfc}} \propto a'\hat{\mathbf{s}}_1 \cdot \hat{\mathbf{I}} + a'\hat{\mathbf{s}}_2 \cdot \hat{\mathbf{I}} = a'\hat{\mathbf{S}} \cdot \hat{\mathbf{I}} \tag{11}$$

and equivalently for $H^{(2)}$. It is important to *stress* that equation 10 giving the hyperfine parameter in the exchange coupled system is *only* valid in the limit of $\zeta \ll 1$.
Effectively, the hyperfine coupling to a particular nucleus in **FAAF** is asymptotically half the coupling to the same nucleus in **AAF**. This observation might naïvely seem paradoxical: there are two equivalent electron spins each generating a hyperfine field at the nucleus, each electron spends only half of its time on each side of the **FAAF** molecule, and so, the total field experienced by the nucleus should be $a/2 + a/2 = a$.(!) Yet, the model above might be misconstrued to predict that the hyperfine field experienced by the nucleus is halved in **FAAF** relative to **AAF**. This is not actually the case, only the hyperfine coupling parameter is halved from a in **AAF** to $a/2$ in **FAAF**.
The hyperfine *field* experienced by the nucleus is proportional to the the hyperfine *coupling* multiplied by the electron spin projection quantum number, M_S: (1) in **AAF** a hyperfine *coupling a* induces a hyperfine *field* equal to $aM_S = \pm a/2$, (2) in **FAAF**, a hyperfine coupling $a/2$ induces a hyperfine field equal to $aM_S/2 = 0, \pm a/2$. Therefore, the $|T_\pm\rangle$ electronic states of **FAAF** induce the same hyperfine field at the nucleus as the $|\alpha\rangle/|\beta\rangle$ states in **AAF**. Naturally, the nucleus does not experience a hyperfine field when the total electronic spin state is $|S\rangle$ or $|T_0\rangle$.

3 Distinguishing doublets and triplets by ESR

This section provides a brief description of how different ESR techniques may be used to probe the electronic spin state of diradicals such as **FAAF**. Naturally, such measurements of the spin quantum number require a reference point. For **FAAF**, this reference point arises from two important sources: (1) the ^1H and ^{13}C nuclei present in the system *via* their hyperfine couplings, (2) by comparison of **FAAF** with **AAF**, the latter having a clear and well-defined spin quantum number of 1/2.

3.1 Continuous-wave ESR

Room temperature liquid solutions of organic radicals akin to the ones of interest here are usually in the rapid tumbling regime. Therefore, the observed cwESR spectrum will only depend on the isotropic g-values and on the isotropic hyperfine couplings (Fermi contact interaction). The dipolar coupling of diradicals, quantified by **D**, vanishes in the rapid tumbling limit. The exchange coupling term, J, is also irrelevant– for ESR– if the two electrons are in very similar chemical environments, as is the case for the **FAAF** system. The effect of J is simply to lift the degeneracy of the singlet and triplet states. Due to the well-known selection rules ($\Delta S = 0$ and $\Delta M_S = \pm 1$), ESR transitions will only be observed within the triplet manifold of the diradical. On the other hand, if J is vanishingly small, the diradical spectrum will be akin to a monoradical spectrum.
In light of the above, the only hope for a cwESR based distinction between species with effective spin 1 or 1/2 lies in the properties of the hyperfine splitting patterns. Importantly however, this distinction is not always possible.

For simplicity, consider two effective electronic spins with similar g-values, one with $S = 1$ and the other with $S = 1/2$, each coupled to a *single* proton with hyperfine couplings a and a', respectively. The two ESR spectra would be a pair of equal intensity peaks split by a for the doublet and a' for the triplet. Therefore, any distinction between the two species can only be made on the basis of *a priori* knowledge about the relative magnitudes of a and a'. However, suppose that the triplet has two equivalent protons with hyperfine coupling a' (or more generally: for every proton in the doublet, there are two corresponding protons in the triplet – akin to **AAF** *vs.* **FAAF**). In this case, the triplet spectrum will be distinct and identifiable: three peaks would be observed with splittings of a' and a $1:2:1$ intensity pattern.
The ideas above were exploited in the interpretation of the cwESR signatures of **FAAF** and **AAF** using numerical spectral simulations. However, since these two systems have relatively large numbers of protons with both resolved and unresolved hyperfine couplings, the interpretation of the cwESR data relied on additional experiments able to probe the hyperfine couplings with higher resolution (pulse ESR and ENDOR).

3.2 Pulse ESR and electron-nuclear double resonance

Continuous-wave ESR alone is not sufficient to ascertain the spin state of **FAAF**. For this purpose, a range of pulse ESR and ENDOR experiments were carried out on **AAF** and **FAAF** in frozen solutions at cryogenic temperatures. All the pulse ESR and ENDOR techniques relevant to this work are described fully in the standard ESR literature.[5–7] However, these techniques are utilised here for a more specialised purpose which is neither commonly addressed nor immediately transparent from the general ESR literature, *i.e.* for establishing the 'tell-tale' signs which discriminate between doublets and triplets. It is therefore vital to give a generalised account of particular pulse ESR and ENDOR effects which render such a distinction possible.
Doublets and triplets may be distinguished on the basis of the following properties, all of which are observable by pulse ESR/ENDOR:

1. the frozen solution ESR spectrum of the triplet will show features attributable to the dipolar interaction,
2. the nutation frequency of triplet states is larger by a factor of $\sqrt{2}$ compared to doublet states,
3. triplet state ^{1}H-ENDOR spectra typically have a distinct peak at the ^{1}H Larmor frequency and, under certain experimental conditions, pronounced asymmetry relative to this peak,
4. the asymmetry of the triplet ^{1}H-ENDOR spectrum may be linked to the electronic spin states participating in the ENDOR pulse sequence,
5. doublet and triplet states show different hyperfine correlation patterns in two-dimensional ENDOR experiments.

All five of the above points stem from the following simplified spin Hamiltonians:

$$\hat{H}_{\mathrm{D}} = \mu_B g B_0 \hat{s}_z - \gamma B_0 \hat{I}_z + a \hat{s}_z \hat{I}_z \tag{12}$$

for doublets, and:

$$\hat{H}_{\mathrm{T}} = \mu_B g B_0 \hat{S}_z - \gamma B_0 \hat{I}_z + D \hat{S}_z^2 + a' \hat{S}_z \hat{I}_z \tag{13}$$

for triplets. The lower-case and capitalised electron spin operators, $\hat{s}_z$ and $\hat{S}_z$, correspond to doublets and triplets, respectively. Each system has a single hyperfine coupling to a spin−1/2 nucleus with gyromagnetic ratio γ. In the interest of generality, the triplet hyperfine coupling is taken to be distinct from the doublet coupling, $a' \neq a$. Furthermore, in the high-field limit, only the secular part (*e.g.* $\hat{S}_z\hat{I}_z$) of the hyperfine coupling is relevant. The triplet also exhibits a secular dipolar interaction quantified by the D-value. These two Hamiltonians are only used for establishing general differences between doublets and triplets. None of the arguments in the paper are influenced by the anisotropies of the hyperfine interaction or by the replacement of the general dipolar coupling tensor, **D**, with the secular D-value. As per the discussion in the previous subsection, the two electrons of the triplet diradical are taken to be equivalent, and thus, J may be omitted from the analysis and the singlet manifold becomes irrelevant.
The energy level diagram derived from $\hat{H}_D$ is shown in figure S15. The ESR spectrum (in frequency domain) of the doublet corresponds to electron spin flips which give rise to two peaks centered at the electron Zeeman frequency, $\mu_B g B_0$, and split by the hyperfine coupling a. Regarding ENDOR, we will only discuss the Mims pulse sequence which is most appropriate for systems with small hyperfine couplings, such as the **FAAF** and **AAF** radicals.[6] The experiment comprises a stimulated echo on the electron spin combined with a 180° radiofrequency pulse appied during the second microwave inter-pulse delay. By monitoring the intensity of the stimulated echo as a function of radiofrequency, an ENDOR spectrum is obtained corresponding to nuclear spin flips induced by the radio pulse with frequency ω_{rf}. These spin flips give rise to two ENDOR peaks at $\omega_{\mathrm{rf}} = \gamma B_0 \pm \frac{a}{2}$, as shown in figure S15.

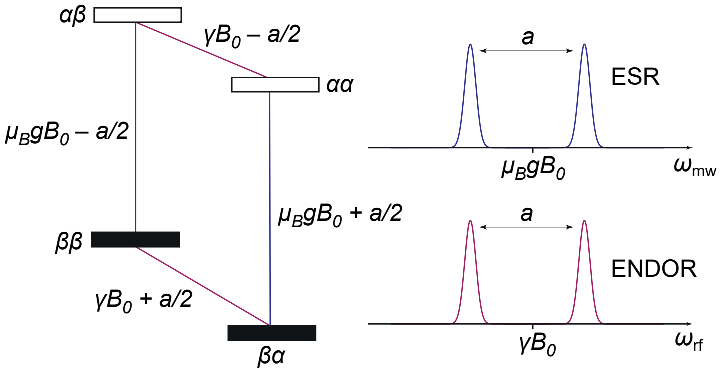

Figure S15: (Left) Energy level diagram for doublets derived from the Hamiltonian in equation 12. The labelling of the quantum states should be interpreted as follows: the first letter indicates the electronic spin projection, α or β, whereas the second letter indicates the nuclear spin projection. The black boxes represent an excess of thermal equilibrium population of the indicated quantum states relative to the states marked with white boxes. The electron spin transitions are marked with blue lines whereas the nuclear spin transitions are marked with red. (Right) Idealised ESR and ENDOR spectra (in frequency domain) resulting from the energy level diagram.

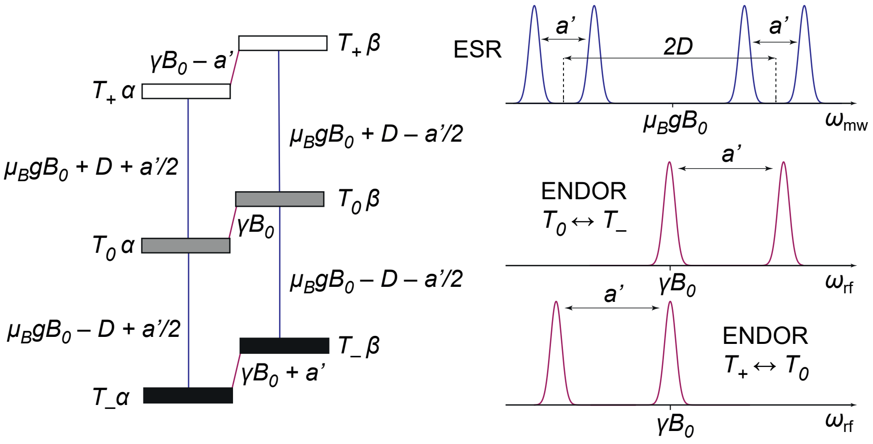

Figure S16: (Left) Energy level diagram for triplets derived from the Hamiltonian in equation 13. By contrast with figure S15, the electronic spin state is denoted by $T_{0,\pm}$ corresponding to the three projection quantum numbers of the triplet state. The shading of the boxes represents the thermal populations of the states; whiter boxes have lower populations. (Right) The top diagram is the idealised ESR spectrum in frequency domain which contains splittings due to both the hyperfine coupling a' and the dipolar coupling D. The bottom two diagrams are the ENDOR spectra corresponding to microwave pulses which select either the $T_+ \rightarrow T_0$ or the $T_0 \rightarrow T_-$ electron spin transitions. By contrast with the doublet case (figure S15), the ENDOR spectra are asymmetric relative to the Larmor peak at γB_0 (if the pulse bandwidths are less than $2|D|$). For triplets, if the sign of D is known, the identity of the electronic spin transition may be inferred from the position in the ESR spectrum and hence, the sign of the hyperfine coupling may be determined from the positions of the hyperfine peaks relative to the Larmor peaks. In the case shown here, the low-field (low-frequency) side corresponds to the $T_- \rightarrow T_0$ transition and positive hyperfine couplings appear on the right side of the Larmor peak.

The triplet state energy level diagram, derived from $\hat{H}_T$, is shown in figure S16. In this case, there are two possible electron spin transitions observable in the ESR spectrum: the $T_+ \rightarrow T_0$ and $T_0 \rightarrow T_-$ transition. Each of these transitions is split further by the hyperfine coupling, a', into two peaks. Hence, the ESR signature will show two pairs of peaks centered at $\mu_B g B_0 \pm D$ and split by a' as shown by the idealised ESR spectrum in figure S16. As may be seen in the data in the main text, in realistic frozen solution spectra, the anisotropy of the dipolar interaction will cause the appearance of a Pake pattern with turning points related to the magnitude of D. Although rarely the case, if a' is large enough, a hyperfine splitting may still be observed superimposed on the Pake pattern. The remaining discussion is still valid despite the complications caused by the Pake pattern.
The ENDOR spectrum of the triplet state is more intricate because it depends on the bandwidths of the microwave pulses relative to the D-value. If the bandwidth of the pulses is larger than $2D$, both electron spin transitions, $T_+ \rightarrow T_0$ and $T_0 \rightarrow T_-$, participate in the experiment. Therefore, all possible nuclear spin flips are observed and

the spectrum will contain a central Larmor peak at γB_0, and two peripheral peaks at $\nu_{\text{rf}} = \gamma B_0 \pm a'$. The Larmor peak is caused by nuclei which are coupled to the electronic T_0 state. The presence of a sharp and distinct Larmor peak is an identifying feature of the triplet state ENDOR spectrum; it is absent in the doublet state.

On the other hand, if the bandwidth of the microwave pulses is smaller than $2D$, then it is possible to address the two electron spin transitions separately by changing the carrier frequency of the pulses, or more commonly, the field position in the ESR spectrum. When the $T_- \rightarrow T_0$ transition is used for the polarisation transfer step in the ENDOR sequence, the resulting spectrum will contain only two peaks at $\nu_{\text{rf}} = \gamma B_0$ and $\nu_{\text{rf}} = \gamma B_0 + a'$. The $T_0 \rightarrow T_+$ transition leads to a 'mirror image' ENDOR spectrum with peaks at $\nu_{\text{rf}} = \gamma B_0$ and $\nu_{\text{rf}} = \gamma B_0 - a'$. This mirror image symmetry between the ENDOR spectra is another identifying feature of the triplet state; such an effect is not possible for doublets.

The above differences between the ENDOR spectra of doublets *vs.* triplets become even more glaring when the spectra are resolved into a second dimension. This may be achieved using hyperfine-correlated ENDOR (or simply, 2D-ENDOR).[5–7] The abscissa of a 2D-ENDOR spectrum is the same as the ω_{rf} axis in 1D-ENDOR. The ordinate in 2D-ENDOR is referred to as the hyperfine dimension. In other words, each peak in the usual 1D-ENDOR spectrum is correlated with the actual hyperfine coupling of the nucleus. In the doublet system, the ENDOR peaks centered at $\gamma B_0 \pm \frac{a}{2}$ in the radiofrequency dimension will be correlated to a hyperfine coupling of a. For molecules with more than one spin$-1/2$ nucleus, this means that hyperfine correlation ridges will be observed along the line $2|\omega_{\text{rf}} - \gamma B_0|$, *i.e.* the ridges will be along lines with gradient 2. By contrast, the hyperfine correlation ridges of the triplet state will be along the $|\omega_{\text{rf}} - \gamma B_0|$ line, *i.e.* along the lines with gradient 1. The factor of two ratio between the gradients of the hyperfine correlation patterns for doublets *vs.* triplets offers a method for measuring the electronic spin quantum number. Essentially, the value of S can be determined by using the nuclear spin quantum number of the protons as an internal reference.

The electronic spin quantum number can also be determined experimentally by measuring the nutation frequency (Rabi frequency) of an electronic transition.[5] Briefly, in the presence of a microwave pulse resonant with a particular electronic transition, say $|S, M_S\rangle \leftrightarrow |S, M_S+1\rangle$, the population difference between the states oscillates at a frequency proportional to $\sqrt{S(S+1) - M_S(M_S+1)}$. For doublets, this factor is 1, and for triplets, it is $\sqrt{2}$ *i.e.* all else being equal, the triplet nutation frequency is larger by a factor of $\sqrt{2}$ when referenced against a doublet spin transition. By contrast with the hyperfine correlation (2D-ENDOR) method discussed above, the determination of the electronic spin state *via* nutation experiments requires a reference spin system with a known value of S, or alternatively, the amplitude of the microwave magnetic field must be known accurately. In this paper, both 2D-ENDOR and nutation experiments will be used to investigate the spin state of the **FAAF** and **AAF** systems.

4 Experimental ESR details

4.1 Sample preparation

Purified powder samples of **FAAF** and **AAF** (with and without ^{13}C isotopic labelling) were synthesized according to the discussion in section 1. ESR measurements were carried out on liquid/frozen solution samples obtained by dissolving the powders to a concentration of approximately 100 μM. Unless otherwise stated, dry dichloromethane was used as the solvent for cwESR. For the pulse measurements, deuterated toluene was used. The solutions were kept under an argon atmosphere for the room temperature measurements. For the *in situ* reactions, solutions of the alcohol/diol precursor species of **FAAF** and **AAF** were added to $SnCl_2$ powder. For the *in situ* cwESR, the reduction was carried out in the ESR tube and monitored in real time. For the pulse measurements, aliquots of the reaction mixture were taken at regular intervals, frozen in liquid nitrogen, and measured as per the methods indicated below.

4.2 Countinous-wave ESR

All cwESR measurements were carried out at room temperature on a Bruker EMX Micro spectrometer operating at a frequency of ca. 9.37 GHz (X-band). The microwave power was adjusted to ensure the signal is not saturated; a power of 2 mW (correspooding to 20 dB) was appropriate for all the samples. The spectra were acquired with field modulation at a frequency of 100 kHz and an amplitude of 0.05 mT. The total number of points was varied on a case by case basis, however, in all cases 10 points per modulation amplitude were used (all other parameters were adjusted accordingly following standard cwESR procedures). The time-dependent measurements of the *in situ* reactions were measured in the same manner as above with the time points chosen as mentioned in the main text of the article.

4.3 FID detected field swept spectra

All pulse measurements were done at 80 K on a Bruker ElexSys E580 Q-band spectrometer equipped with a EN5107D2 resonator. The temperature was controlled using liquid N_2 and an Oxford Instruments cryostat. Un-

less otherwise stated, free induction decay (FID) field-swept spectra were acquired using an 800 ns low power microwave pulse. The FID signal was subsequently integrated to yield the final spectra.

4.4 Electron-nuclear double resonance

The 1D-ENDOR measurements were performed using the Mims sequence with 54 ns microwave pulses and a 24 μs radiofrequency (rf) pulse. This microwave pulse length corresponds to a bandwidth of approximately 22.4 MHz which is smaller than the estimated dipolar coupling constant, thus allowing selective excitation of the individual triplet transitions of **FAAF**. The stimulated echo delay was 120 ns for the 1D measurements. For the 2D-ENDOR measurements, this delay was incremented in steps of 4 ns over 512 points with an initial value of 120 ns. The length of the rf pulse was decided from the results of an rf nutation experiment.

4.5 Data processing and simulations

The cwESR spectra were baseline corrected and the magnetic field values were scaled to a common frequency of 9.4 GHz for all the reported data sets. Since the spin systems investigated here are truly in the high-field limit, the scaling of the field axis induces negligible distortions and facilitates a more meaningful discussion of the hyperfine splitting patterns. The exact g-values of these radicals are not of interest in the present work. For most spectra, the intensity was normalised to a unit maximum absolute value. Similarly, all FID spectra were baseline corrected and adjusted to a common frequency of 34 GHz. For 1D-ENDOR, the spectra were baseline corrected, inverted, normalised, and the ν_{rf} axis was replaced by $\nu_{\mathrm{rf}}-\nu_{\mathrm{H}}$, where ν_{H} is the proton Larmor frequency at the corresponding magnetic field. The final step ensures that spectra acquired at different field positions are directly comparable. For 2D-ENDOR, the data was processed in the same manner along the rf dimension. The hyperfine dimension was obtained from the time dimension by cubic baseline subtraction, apodization with a Hamming window, zero-filling to the second nearest power of 2 bigger than the number of points, and Fourier transformation using *fft* in MATLAB. All data analysis and spectral simulations presented in this paper were done using the EasySpin toolbox in MATLAB.[8]

5 Further ESR results

In this section, we explore the experimental evidence for the statement in the main text according to which the presence of a doublet/triplet admixture in liquid solutions of **FAAF** is likely caused by a large number of factors: temperature, solubility, interactions with oxygen, and interactions with light.

5.1 The effects of thermal cycling

One of the possible origins of the doublet component present in solutions of **FAAF** is the formation of intermolecular aggregates with net spin-1/2. For instance, when two **FAAF** molecules stack off-center, two of the fluorenyl fragments may pair their spins, and the other two would remain far apart and effectively non-interacting. Additionally, the delocalisation of the spin density across more than one molecule would lead to a broader distribution of hyperfine couplings and hence, to broader cwESR spectra. As is the case for many aromatic compounds, **FAAF** and **AAF** may indeed form aggregates by π-stacking.[9,10] A simple test of this hypothesis would be to measure the cwESR spectra of liquid solutions before and after heating the samples. The results of this experiment are shown in Figure S17.

All systems display a clear increase in super-hyperfine resolution after heating. This higher resolution disappears after storing the samples in a freezer (for approximately 30 minutes) as shown in panels (a) and (c) . Simulations of the **FAAF** spectra (panel (d)) reveal a modest increase in the triplet component after heating. However, the large number (i.e. 30) of parameters required in the fitting, makes an analysis of the uniqueness of the results difficult.

Whilst aggregation processes may indeed be consistent with the data shown in Figure S17, a different hypothesis is actually significantly more likely. As will be shown below, the same increase in resolution may be induced for all samples by degassing. It is therefore possible that heating the solutions causes a decrease in oxygen concentration, which is then responsible for the spectra observed in Figure S17.

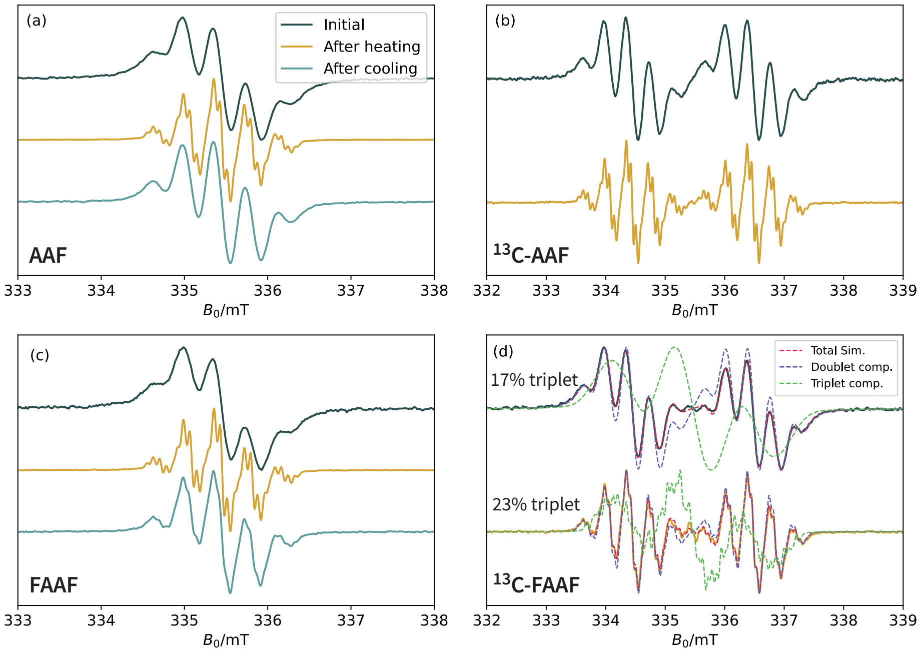

Figure S17: X-band cwESR (DCM) spectra of the purified **AAF** and **FAAF** radicals performed before and after heating the same solution (by inserting the ESR tube in 90°C water for ca. 5 minutes). At the point of measurement, the solutions are at room temperature. (a), (b) The results for the unlabelled and labelled **AAF** species. After heating the resolution of the ESR spectrum increases. After cooling the same sample, the resolution decreases. (c), (d) Equivalent measurements and simulations on the **FAAF** diradical. Simulations of the ^{13}C labelled spectra reveal a modest, yet clear, increase in the triplet component after heating. The unlabelled spectra of **FAAF** are too similar to **AAF** to allow any meaningful simulations.

5.2 Interactions with molecular oxygen

Another potential explanation for the presence of a doublet in liquid solutions of **FAAF** is from a peroxidation reaction. In the presence of molecular oxygen, it is possible that two peroxides, **FAAF**-OO$^\bullet$ and $^\bullet$OO-**FAAF**-OO$^\bullet$, may be formed. Both of these peroxides would have a net spin-1/2 because the delocalisation of the spin density across the π-system would be inhibited and hence, the overlap of molecular orbitals (and the exchange interaction) would decrease. Furthermore, the increased torsional freedom of the peroxide groups would result in a wider distribution of hyperfine couplings and hence, a lower resolution in cwESR. Furthermore, if the peroxides are labile, their presence would not be detectable by mass spectrometry and the dynamic equilibrium would lead to exchange broadened cwESR spectra (as well as the wider hyperfine spectral distribution).

Another mechanism by which molecular oxygen (a triplet state) can interact with the radicals is via the magnetic dipolar interaction. The rotational motion of the oxygen molecules causes a broad spectrum of fluctuating electromagnetic fields which may contribute to the spin relaxation of the radicals. This would also result in a decrease in resolution of the corresponding cwESR spectra.

To test the above hypotheses, four cwESR spectra were measured on each of the four species (**AAF** and **FAAF** with and without ^{13}C) under the following conditions: (F) fresh, ambient DCM solutions prepared from purified powders, (D) after degassing the solution by four freeze-pump-thaw cycles in an ESR tube equipped with a J. Young tap, (O_2) after opening the tap and bubbling pure oxygen gas through the solution for less than 5 seconds, (D') after degassing the oxygenated solution again. The oxygen bubbling was done through a 30 cm long syringe needle attached to a balloon filled with oxygen at a pressure slightly above the onset of the Laplace limit. The process must be done very quickly to avoid evaporation of the volatile DCM solvent. The results are shown in Figure S18.

The conclusion from the data in Figure S18 is that oxygen plays a crucial role in determining the resolution of the cwESR spectrum. The oxygenated samples exhibit a complete loss of ^{1}H hyperfine resolution. The ^{13}C couplings persist, albeit they are still broadened. The spectral simulations of the **FAAF** samples before and after degassing show a modest increase in the triplet contribution (again, the parameter space has dimension 30).

Equivalent FID spectra at 80 K were attempted (data not shown). Unfortunately, we were unable to obtain reproducible results. We were unable to conclude whether the triplet composition increased/decreased from the 80 K spectra. Nonetheless, the oxygenated solutions did not show such a dramatic loss of resolution in the 80 K spectra. This suggests that the main mechanism for broadening is perhaps via an increase in relaxation rates rather than via reversible peroxidation. The rotational motion of the oxygen molecules is slower at 80 K and hence, it contributes less to the relaxation rates at lower temperatures (see below for relaxation measurements).

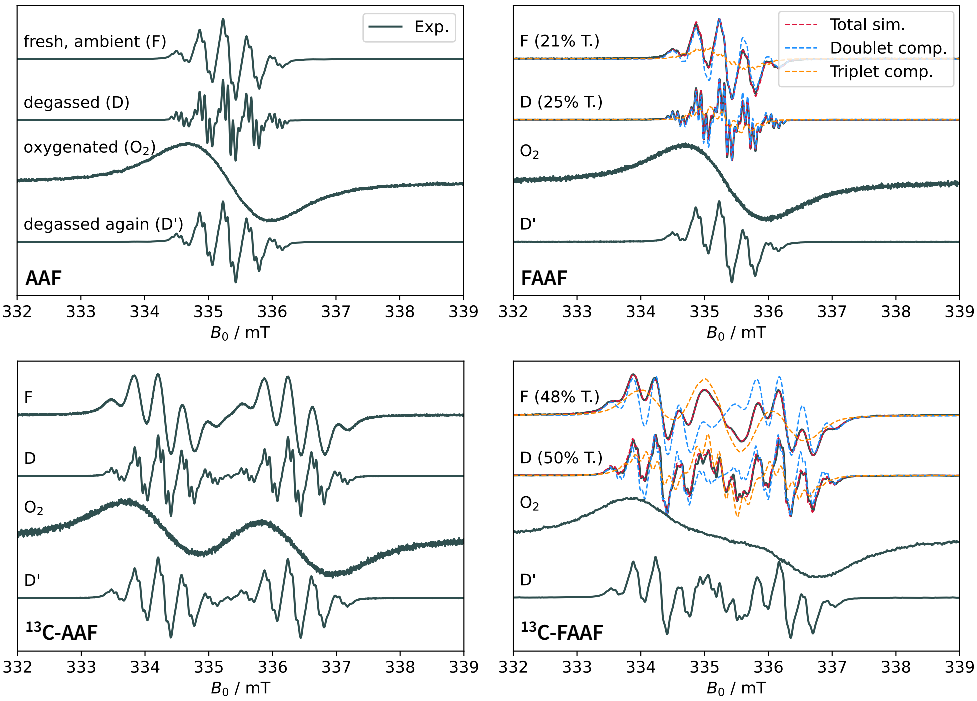

Figure S18: (Top) The cwESR spectra of the unlabelled **AAF** and **FAAF** species measured in DCM at room temperature before and after degassing, re-oxygenating, and a final round of degassing. (Bottom) The equivalent spectra for the ^{13}C-labelled **AAF** and **FAAF** species.

5.3 The effects of light

. The data presented in Figure S19 show that liquid solutions of **AAF** and **FAAF** bleach rapidly in the presence of light. For example, in sunlight, solutions will turn from pink to transparent in a matter of minutes and the radicals signals vanish. Therefore, interactions with light may also be responsible for the presence of a doublet component in solutions of **FAAF**. A partial light-induced quenching of the diradical, to a different (unknown), fluorenyl-based monoradical cannot be ruled out under ambient laboratory lighting conditions. Such a bleaching process would also contribute to the variations in the triplet/doublet concentrations observed between different runs of the experiment.

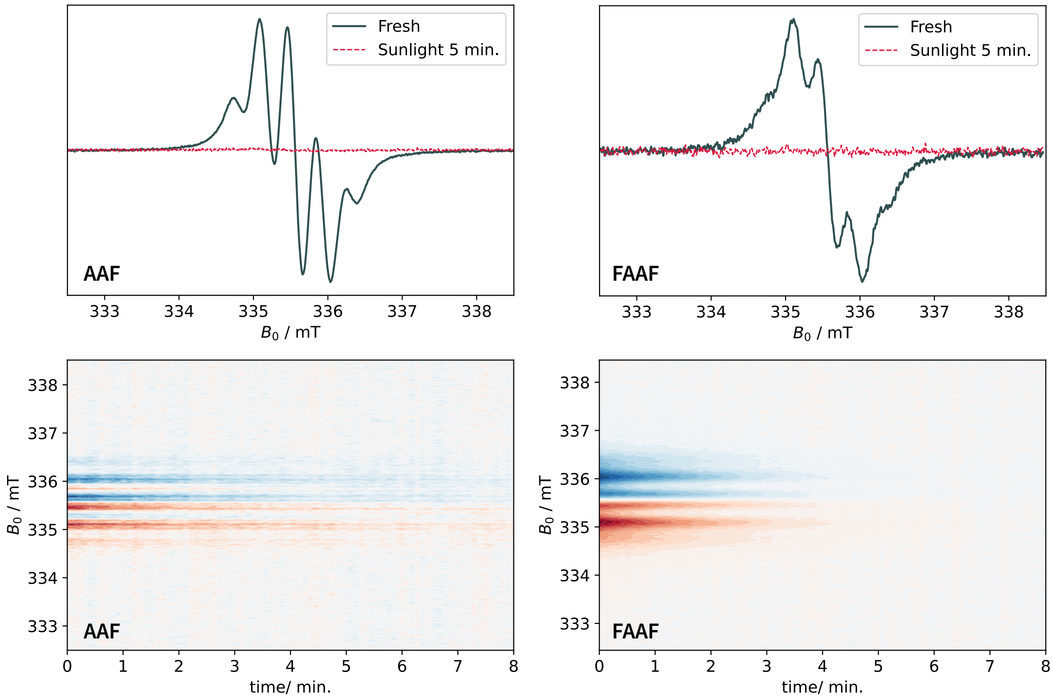

Figure S19: (Top) cwESR spectra of **AAF** and **FAAF** obtained before and after exposing the ESR tube to direct sunlight for 5 minutes (DCM, ambient conditions). (Bottom) cwESR spectra of **AAF** and **FAAF** recorded over the course of irradiation for 8 minutes with a 340 nm LED (50 mW LED power, DCM solvent, ambient conditions). The colormap is RdBu from the matplotlib library, well-known for positive peaks being red and negative peaks being blue. The irradiation was performed by attaching the LED directly to the optical window of the cryostat.

5.4 *In-situ* radical formation

As mentioned in the main text, the doublet component of **FAAF** solutions tends to be dominant in most samples prepared from purified powders. In the main text, a variety of ESR-based evidence has been presented to show that liquid solutions of **FAAF** contain a mixture of spin states. Additionally, we propose a versatile experimental procedure, 2D-ENDOR, which can be used more widely in future studies of similar organic multi-radicals to determine their total spin quantum numbers. Now, as far as **FAAF** is concerned, the question of the origin of the doublet component remains. As mentioned in the above sections, a number of non-trivial factors could be responsible for the doublet component. However, it is important to highlight that this mixture of spin states, and the presence of the doublet component in particular, is not resolved clearly (or at all) in the *non*-ESR techniques used throughout the synthesis and characterisation of the compounds. Even more puzzling is the fact that the doublet component is not an impurity because it both dominates in solutions prepared from purified **FAAF** powders and it has all the hyperfine couplings expected in a fluorenyl-based monoradical. In light of this, another question surfaces: is it possible to prepare a solution of **FAAF** in which the triplet component dominates?
Answering the above question involves monitoring the formation of the **FAAF** diradical from the reaction of the diol precursor (**3a** in figure 2 of the main text) with $SnCl_2$ in dichloromethane. The results of this procedure are illustrated in figure S20. The $SnCl_2$ reducing agent is insoluble in dichloromethane and thus, the reaction with the diol proceeds slowly (over the course of minutes/hours) at the solid-liquid interface. Once the triplet diradical is formed, it diffuses from this interface into the bulk solution phase. As the diradical concentration increases in the solution phase, the interactions with molecular oxygen or with other diradical molecules become more frequent and eventually lead to the formation of the doublet component. Since all of these events occur on a

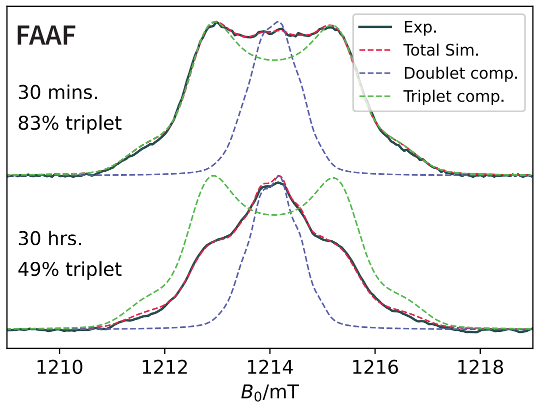

Figure S20: Q-band FID detected field-swept spectra of aliquots taken from the reaction mixture of diol **3a** (figure 2 in the main text) with $SnCl_2$ at two different times. The spectra are recorded at 80 K. The spectral simulations employ the same parameters as shown in table 2 of the main text, apart from the doublet/triplet weighting. After 30 minutes, the triplet component dominates at 83% whereas the weighting of the doublet component increases slowly to 51% after 30 hours.

time-scale of minutes, it is possible to extract aliquots of the reaction mixture at different times, freeze them in liquid N_2 to halt the dynamics, and perform ESR measurements. When the aliquots are extracted from the top of the solution, the solid $SnCl_2$ remains in the original mixture, and so, the reduction effectively stops. Furthermore, by flash-freezing the aliquot in liquid nitrogen we also inhibit any diffusion limited processes which may lead to the formation of the doublet component. In the early stages of the reaction, when the triplet contribution is 83%, it is possible that the remaining 17% doublet contribution is due to species in which one of the hydroxyl groups has not been reduced (a monoradical-alcohol species). It is therefore not clear from this data alone whether the doublet species is only due to interactions with oxygen or partial reduction. Overall, the results shown in figure S20 are consistent with the processes described above: (1) nascent aliquots (30 minutes) show a dominant triplet contribution, (2) later aliquots (30 hours) show a clear increase of the doublet component.

In figure S21, we show the equivalent experiments performed using cwESR detection. The experiments were performed as follows: (1) a small amount of $SnCl_2$ was placed at the bottom of an ESR tube, (2) the air was carefully evacuated from the tube whilst sealed with a septum, (3) the tube was backfilled with argon, (4) the needle of an air-tight Hamilton syringe filled with 100μL of dilute (200μM) diol/alcohol solution in DCM was pierced through the septum, but the solution was not yet injected, (5) the microwaves were tuned using an ESR tube containing only 100μL of solvent, (6) afterwards the tube with the syringe still attached was inserted in the resonator at the same depth as the tuning tube, (7) the solution was injected and the microwaves were re-tuned very slightly for approximately 5 seconds before starting the 2D scans. The data was collected over a period of 16 hours at a rate of 25 seconds per sweep.

The cwESR spectrum recorded in the nascent stages of the **AAF** radical formation displays the same higher resolution as was seen in subsections (5.2) and (5.3). As the reaction proceeds, the super-hyperfine structure is lost and the spectrum starts to resemble more closely the one obtained for ambient solutions. The simulations shown in Figure S21 reveal that the magnitude of the hyperfine couplings is constant in time; only the inherent linewidth of the peaks is increasing. This suggests that, as time passes, the radical(s) responsible for the signal are still fluorenyl-based.

For **FAAF** the data in figure S21 does show a larger contribution of the triplet component (compared to ambient solutions) which rises to approximately 45% in the first hour and increases slowly to 65% after 16 hours. Overall, the spectra shown in the right panel are caused by a complex interplay of the following factors: the potential initial formation of a monoradical-alcohol from the diol, delayed formation of the diradical from the monoradical-alcohol, the diffusion of the various species to and from the bottom of the ESR tube where the solid $SnCl_2$ is present, the potential for inter-radical aggregates to form as their concentrations rise, the potential for oxygen to leak slowly into the system and cause faster relaxation and/or reversible peroxidation, and the potential partial quenching of the radicals by light.

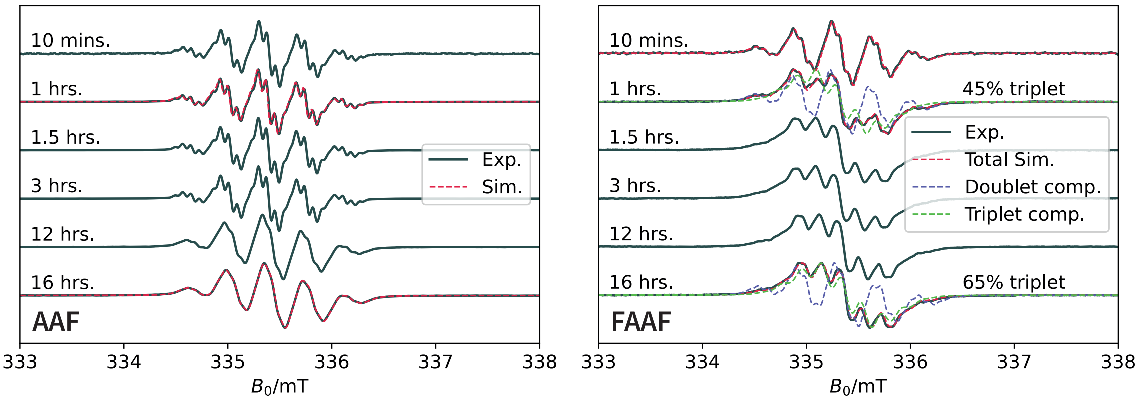

Figure S21: X-band cwESR measurements of the *in situ* reductions of the alochol (left) and diol (right) precursors of **AAF** and **FAAF**. The measurements were performed at room temperature using DCM as the solvent. Solutions containing the alcohol/diol were injected into the ESR tube (already placed in the resonator) containing the $SnCl_2$ reducing agent. Following a swift and sprightly microwave tuning, the time-domain data was collected for 16 hours at a rate of 25 seconds per field sweep. The spectra compiled here were normalised to the maximum absolute value.

5.5 Spin relaxation in FAAF

Spin-spin relaxation. The measurements were performed using the 2-pulse spin echo sequence: $\pi/2 - \tau - \pi - echo$. The pulse lengths were 54 ns and 108 ns for $\pi/2$ and π, respectively. These pulse lengths were chosen such that their bandwidth (22 MHz) allows selective excitation of the doublet and triplet components of the ESR spectrum. The integral of the spin echo was recorded as a function of τ. The field positions are denoted by L, C, and R to denote the left, centre, and right positions, respectively. L = 1212.1 mT, C = 1213.5 mT, and R = 1214.9 mT at a microwave frequency of 34 GHz. L and R correspond to the triplet signals present in the spectrum of **FAAF**. C is the maximum in the spectrum, and corresponds to a predominant doublet contribution.
Spin-lattice relaxation. The measurements were performed using the inversion recovery sequence: $\pi - T - \pi/2 - \tau - \pi - echo$. The pulse lengths were the same as mentioned above. The echo integral was recorded as a function of T and at constant τ = 200 ns. The same field positions as for spin-spin relaxation were chosen.
Data processing. All relaxation data were fitted using the *lsqnonlin* function in MATLAB. For the toluene-D_8 data, the 2-pulse echo decay data was fitted using the following model function:

$$E_1(\tau) = Ae^{-(\tau/T_m)^\alpha} \tag{14}$$

i.e. a stretched exponential decay with initial value A, decay time T_m, and stretch parameter α. For CS_2, it was necessary to consider a sum of two exponentials:

$$E_2(\tau) = A_1 e^{-\tau/T_{m1}} + A_2 e^{-\tau/T_{m2}} \tag{15}$$

The inversion recovery data for both solvents employed the following model function:

$$M(T) = A_1 + A_2 e^{-T/T_1} + A_3 e^{-T/T_1'} \tag{16}$$

i.e. a sum of two exponential decays with characteristic times T_1 and T_1'.

5.5.1 Spin-spin relaxation rates

All the experimental results relevant for this section are summarised in figure S22 and the fitting parameters in table S1. The results obtained in toluene-D_8 illustrate that the triplet component has a faster spin-spin relaxation rate. The similarity of the relaxation times at the L and R field positions is aligned with the fact that these two positions represent the same orientation of the dipolar axis of **FAAF** relative to the magnetic field (*i.e.* 90°). Systems with larger spin quantum numbers interact more strongly with the environmental degrees of freedom. For example, all else being equal, a solvent nucleus will induce a hyperfine field experienced by the electron which is twice as large in a triplet relative to a doublet. At early times, all traces recorded in toluene-D_8 are modulated by the Zeeman frequency of deuterium, suggesting that the primary relaxation mechanism in this solvent is *via* the dipolar interaction with the nuclear spin bath (and not via interactions with molecular oxygen). The data obtained in CS_2 is a superposition of two components with very different relaxation rates. The prompt component has a $T_{m2} = 0.2 - 0.4\,\mu s$ which is two orders of magnitude faster than the slow component T_{m1} =

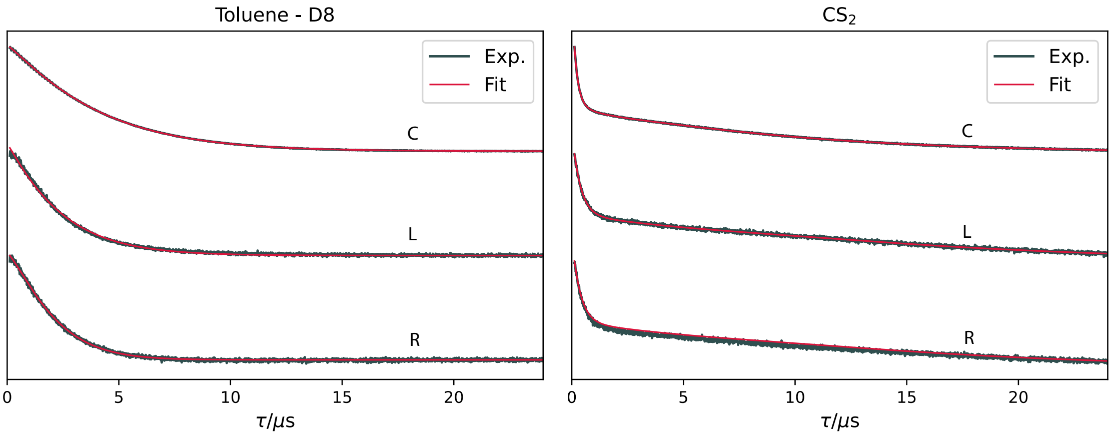

Figure S22: 2-pulse spin echo decay traces for **FAAF** measured in the two solvents at the L, C, and R positions in the ESR spectrum. The fitting parameters are shown in table S1. The data is stacked for clarity; the initial point of all traces is actually 1.

	Toluene - D_8			CS_2	
**Position**	A	T_m / μs	α	A	T_{mi} / μs
**C**	1.0	4.2	1.1	0.4	10
				1.0	0.2
**L**	1.0	2.5	1.1	0.5	15
				0.8	0.3
**R**	1.0	2.2	1.3	0.4	15
				0.9	0.4

Table S1: Spin-spin relaxation parameters determined by fitting $E_1(\tau)$ from equation 14 to the toluene-D_8 data and $E_2(\tau)$ from equation 15 to the CS_2 data.

$10 - 15\,\mu$s. We speculate that the prompt component could be due to the larger triplet contribution at all field positions in this solvent. The slow relaxing component measured in CS_2 is slower than any of the relaxation rates measured in toluene. This is consistent with the fact that in CS_2, the electron spin is in an environment free from nuclear spins (the natural abundance of the spin-3/2 ^{33}S nucleus is 0.76%). However, the solubility of **FAAF** in CS_2 is also lower, and thus, aggregation processes could be contributing to the observed relaxation traces.

5.5.2 Spin-lattice relaxation rates

The inversion recovery experiments (figure S23 and table S2) reveal a very similar story to the observations of the previous section. The triplet component has a faster spin-lattice relaxation rate compared to the doublet component as illustrated particularly clearly by the toluene-D_8 data. The fast component at position C in toluene-D_8 matches nicely to the main triplet relaxation time of ca. 3.5 ms. This is in agreement with the fact that both species contribute to the ESR peak at C. The prompt component at L and R in toluene are also likely the result of aggregation.

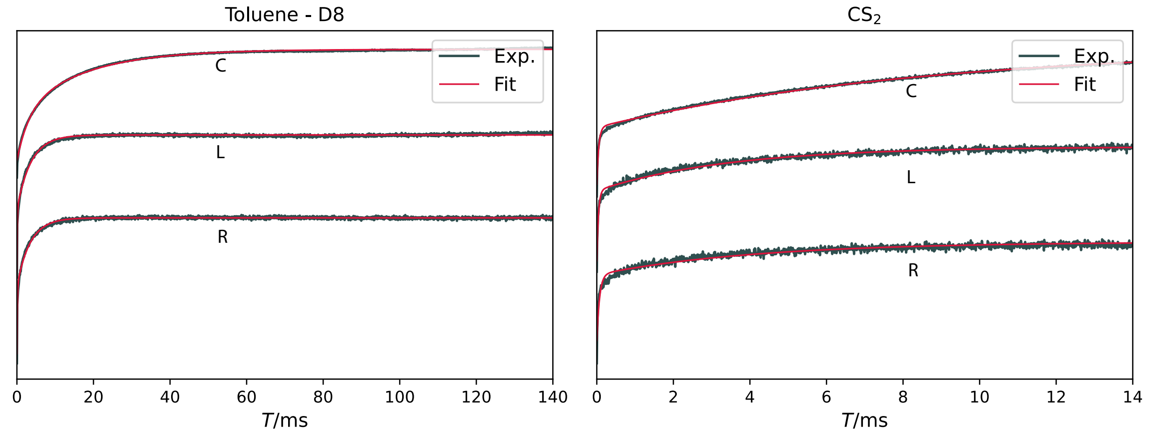

Figure S23: Inversion recovery traces for **FAAF** measured in the two solvents at the L, C, and R positions in the ESR spectrum. The fitting parameters are shown in table S2. The data is stacked for clarity; all traces have been normalised for fitting purposes.

	Toluene - D_8		CS_2	
**Position**	A_i	T_1 / ms<br>T_1' / ms	A_i	T_1 / ms<br>T_1' / ms
**C**	0.97	17.3	1.0	8.6
	-0.77	3.1	-0.87	0.1
	-0.56		-0.61	
**L**	0.95	3.6	0.93	3.4
	-0.89	0.1	-0.47	0.04
	-0.73		-0.75	
**R**	0.96	3.4	0.92	4.2
	-0.81	0.1	-0.35	0.1
	-0.80		-0.70	

Table S2: Spin-lattice relaxation parameters determined by fitting $M(T)$ from equation 16 to all data sets.

5.6 Solvent dependence of FID spectra

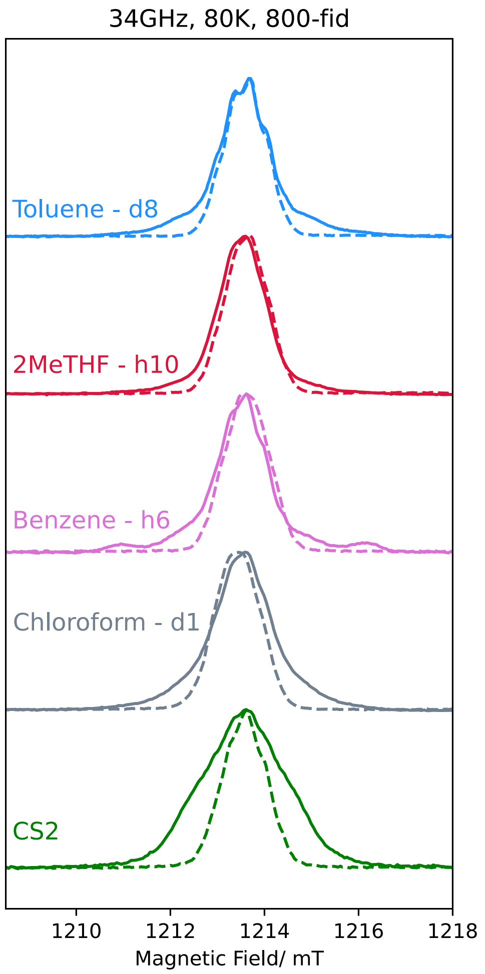

Figure S24: FID detected field swept spectra of **AAF** (dashed lines) and **FAAF** (solid lines) recorded at 80 K and Q-band in 5 different solvents. The overall conclusion is that the relative doublet/triplet contribution is approximately the same in most solvents (from the itensity of the shoulders in the **FAAF** spectra). The only exception is the CS_2 data. However, the larger triplet contribution is not reproducible in this solvent because of the low solubility of the diradical. In chloroform and 2-methyl-tetrahydrofuran, the radical quenches over the course of 10 minutes at room temperature (the pink colour of the solution fades).

5.7 Solvent dependence of Mims ENDOR spectra

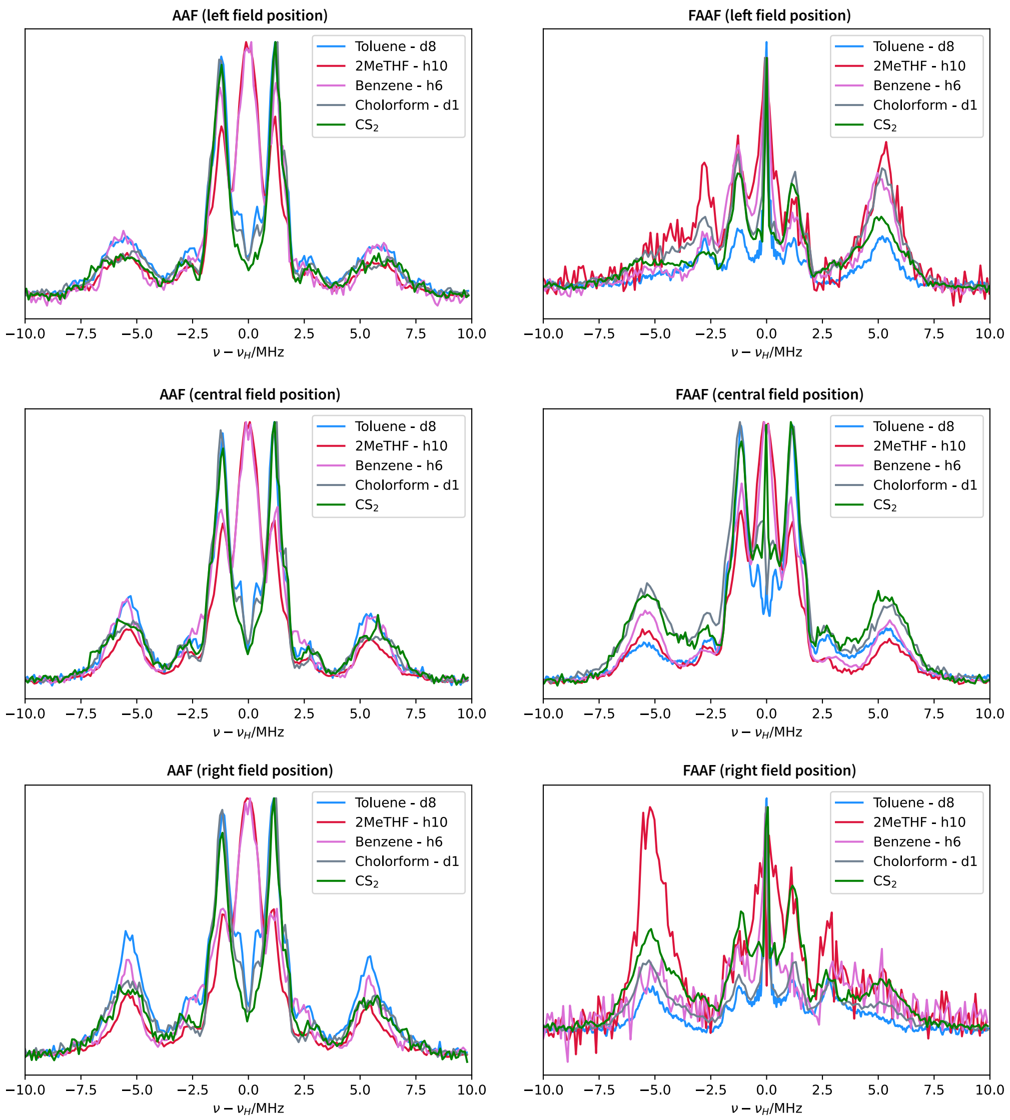

Figure S25: (Left) Mims ENDOR spectra for **AAF** recorded at Q-band at three field positions across the FID spectrum (central ∼ 1213.5 mT, right ∼ 1212.5 mT for **FAAF** and ∼ 1213.0 mT for **AAF**, left ∼ 1214.5 mT for **FAAF** and ∼ 1214.0 mT for **AAF**; all relative to the FID spectra in Figure S24). The ENDOR peak positions remain mostly unaffected by the field position. This is exactly as expected for a spin-1/2 system. (Right) Mims ENDOR spectra for **FAAF** recorded across the FID spectrum. The presence of a sharp Larmor peak and the mirror image of the left/right field positions suggests that the doublet/triplet mixture is present in all solvents.

5.8 Characterisation of the ^{13}C hyperfine coupling in AAF

In the main text, we mentioned that the ^{13}C-labelled systems do not provide a good reference for the electronic spin state at cryogenic temperatures due to the significant anisotropy of the ^{13}C hyperfine coupling. In the following three sections we present the experimental results which led to this conclusion.

5.8.1 Q-band FID spectra

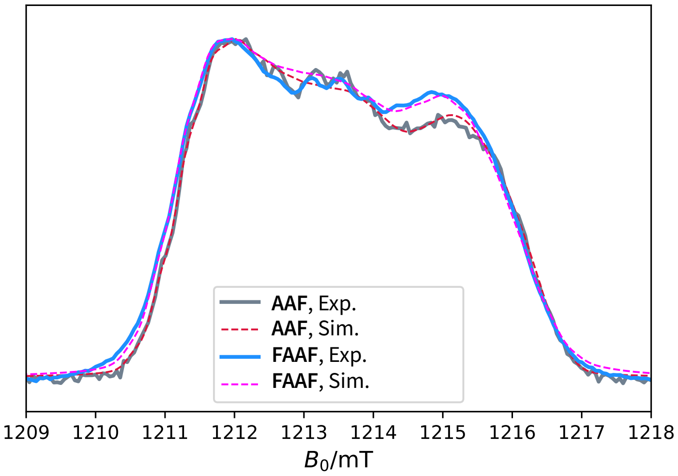

Figure S26: Experimental and simulated FID spectra of the ^{13}C-labelled **AAF** and **FAAF** recorded at 80 K and Q-band microwave frequencies. All simulation parameters were identical to the ones presented in the main text for the unlabelled systems *except* for a ^{13}C-hyperfine coupling with eigenvalues (in MHz) of $A_x = 8$, $A_y = 15$, $A_z = 135$. The isotropic component is $a_{iso} = (A_x + A_y + A_z)/3 \approx 53$ MHz, in good agreement with the value of 54–57 MHz reported in the main text for the cwESR data. The simulation of **FAAF** also includes a doublet/triplet (85/15%) admixture. For the triplet component of **FAAF**, the ^{13}C-hyperfine tensor eigenvalues were obtained from the doublet values divided by 2. However, the anisotropy of the ^{13}C coupling in the doublet masks the fine-structure of the triplet component (note that the D-value is of the order of 70 MHz – from Table 1 in the main text – which is siginificantly smaller than $A_z - A_{x/y} \approx 100$ MHz).

5.8.2 Hyperfine sub-level correlation spectroscopy (HYSCORE)

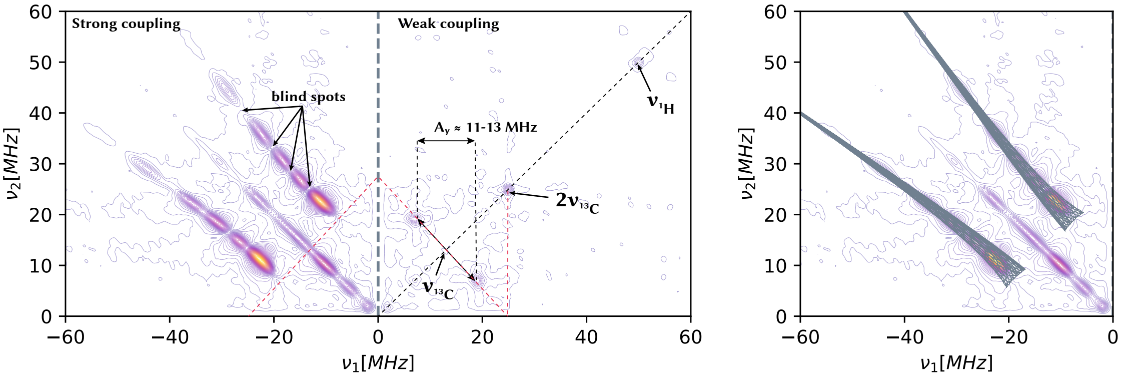

Figure S27: (Left) HYSCORE spectrum of **AAF** recorded at Q-band and 80 K at the central position in the FID spectrum (~1213.5 mT referenced against the spectra in Figure S26). (Right) Correlation pattern predicted using the EasySpin function *nucfreq2d* for the strong coupling quadrant (overlayed in gray on the data from the left panel). A ^{13}C-hyperfine tensor with eigenvalues $A_x = 8$, $A_y = 15$, $A_z = 135$ was used in the calculation.

Experimental details. The samples were prepared as before using non-deuterated solvent (DCM) in order to avoid interference from the deuterium modulations (^{2}H has a similar Larmor frequency to ^{13}C). The HYSCORE spectrum in Figure S27 was obtained using microwave pulse lengths of 10 ns. The power of the mixing pulse was optimised for maximum echo inversion. The stimulated echo delay was 120 ns. The time requirement for

an individual τ-value was ~18 hours and prohibited performing τ-averaging; hence, the data contain blind spots, as indicated in Figure S27. The first and second HYSCORE delays were each incremented in steps of 8 ns over 128 points from an initial value of 48 ns. The central part of the echo was integrated over a 22 ns window. The time domain data was Fourier transformed in both dimensions following a polynomial baseline correction and a zero-filling to 512 points in each dimension.

Key results. The most noteworthy feature of the HYSCORE data in Figure S27 is the presence of intense correlation ridges parallel to the anti-diagonal of the strong coupling quadrant. These two ridges converge to the dashed red line which represents $|\nu_1 - \nu_2| = 2\nu_{^{13}C}$. This situation is consistent with a large hyperfine coupling (compared to the ^{13}C-Larmor frequency) and also with $a_{iso} > T$ where T is the dipolar (anisotropic) component of the hyperfine tensor. These ridges are matched well by the EasySpin predictions shown in the right panel of Figure S27 employing the same hyperfine tensor as used for the simulation of the FID data. In the weak coupling quadrant, we note the appearance of two correlation peaks which we tentatively assign to the smaller in-plane A_y component of the ^{13}C-hyperfine tensor.

HYSCORE experiments performed at other field positions (*e.g.* at the maximum intensity position ~1211.5 mT in Figure S26) did not yield any off-diagonal correlation ridges or peaks. This observation is also consistent with a large and anisotropic ^{13}C-coupling. At these field positions, the mixing pulse (10 ns) is most probably unable to transfer the nuclear coherence between the two electron spin manifolds because it does not have a large enough bandwidth.

5.8.3 Davies ENDOR

As for HYSCORE, the **AAF** sample was measured at Q-band and 80 K in (non-deuterated) DCM. The inversion pulse had a length of 54 ns and it was combined with a Hahn echo detection sequence comprised of 16/32 ns pulses with an echo delay of 300 ns. The powers of the microwave pulses were optimised to ensure the correct flip angles required in Davies ENDOR. The rf pulse length was 20 μs with a power optimised via a nutation experiment. However, care was taken to use a low enough rf power to avoid interference with the 3rd and 5th harmonic excitation of the ^{1}H frequencies (caused by clipping of the rf pulse by the amplifier). This was achieved by trial and error optimisation. The final measurement was taken at the central position in the FID spectrum (1213.5 mT relative to Figure S26.)

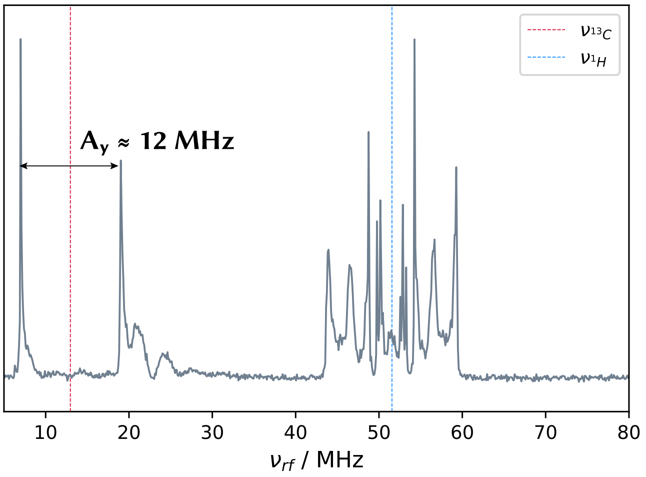

Figure S28: Davies ENDOR spectrum of **AAF** recorded at Q-band and 80 K at the central field position in the ESR spectrum (1213.5 mT). The dashed vertical lines indicate the ^{1}H and ^{13}C Larmor frequencies. In the ^{13}C region, we observe the in-plane component of the hyperfine tensor, similarly to the weak coupling quadrant in HYSCORE. We attempted measurements at different field positions and were, unfortunately, not able to capture the large out-of-plane component of the ^{13}C-hyperfine tensor. This is most likely due to the very broad distribution of ENDOR frequencies caused by such an anisotropic hyperfine coupling. It is, however, plausible that the broad peaks between 20-30 MHz are due, at least partially, to the out-of-plane component of the hyperfine tensor. Numerical simulations did not shed any light on the exact origin of the details present in this Davies ENDOR spectrum.

5.9 Nutation experiments

Experimental procedure. The nutation frequency spectra of the radicals present in frozen (80 K) solutions of **FAAF** in DCM were measured using the pulse sequence: t_p–24 μs–$\pi/2$–300 ns–π–echo at Q-band microwave frequencies. The echo detection pulses both had a length of 54 ns and their individual powers were adjusted to ensure that approximately correct flip angles are achieved (done by increasing the power of the final pulse until the echo is maximised). The length of the nutation pulse was varied from 0 to 1022 ns in steps of 2 ns. The 24 μs delay between the nutation pulse and the detection sequence was chosen to be much longer than T_2 (measured to be ca. 2μs). The results are shown in figure S29.

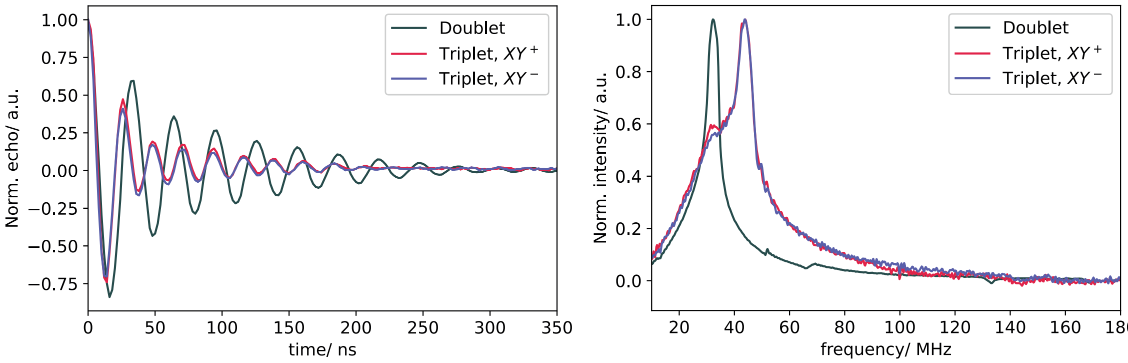

Figure S29: (a) Time domain 3-pulse nutation experiments performed on **FAAF** as per the description given above. The doublet trace (black) was aquired at the field position corresponding to the maximum in the FID spectrum shown in the main text. The triplet trace was acquired at the $XY^{\pm}$ field positions (see main text). (b) Frequency domain nutation spectra obtained by Fourier transformation of the time-domain data. The maximum of the doublet spectrum is at 32.2 MHz whereas the maximum in both triplet spectra is at 43.85 MHz corresponding to a ratio of 1.36 which is within 4% of the theoretical ratio of $\sqrt{2}$ between the triplet/doublet nutation frequencies. It is also clear that the doublet frequency is present in the triplet spectra. This is likely caused by the fact that the bandwidth of the nutation pulse is very large at short times, thus leading to excitation of both components.

5.10 Frozen solution cwESR

The frozen solution cwESR spectra in figure S30 were measured in an attempt to benchmark the results presented in the main text against a more widely available ESR experiment. Unfortunately, the similarity of the unlabelled spectra (left panel) precluded a unique deconvolution of the spectra into the doublet and triplet components. Furthermore, the analysis was complicated by the fact that we were unable to measure unsaturated spectra even when employing the lowest possible microwave power (perhaps because the T_1 times are long, see section 5.5.2). The ^{13}C-labelled spectra are quite different, but the deconvolution was still not possible due to the same issues as mentioned for the unlabelled systems as well as the additional complications introduced by the anisotropy of the ^{13}C hyperfine coupling.

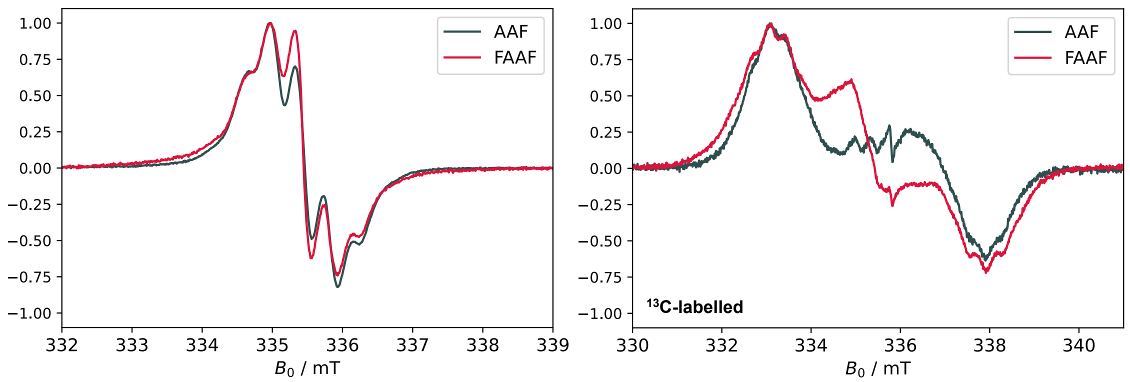

Figure S30: Frozen toluene solution cwESR spectra of **FAAF** and **AAF** measured at X-band and 140 K. The left and right panels refer to the unlabelled and ^{13}C-labelled species, respectively.

6 Density Functional Theory calculations

6.1 Key results

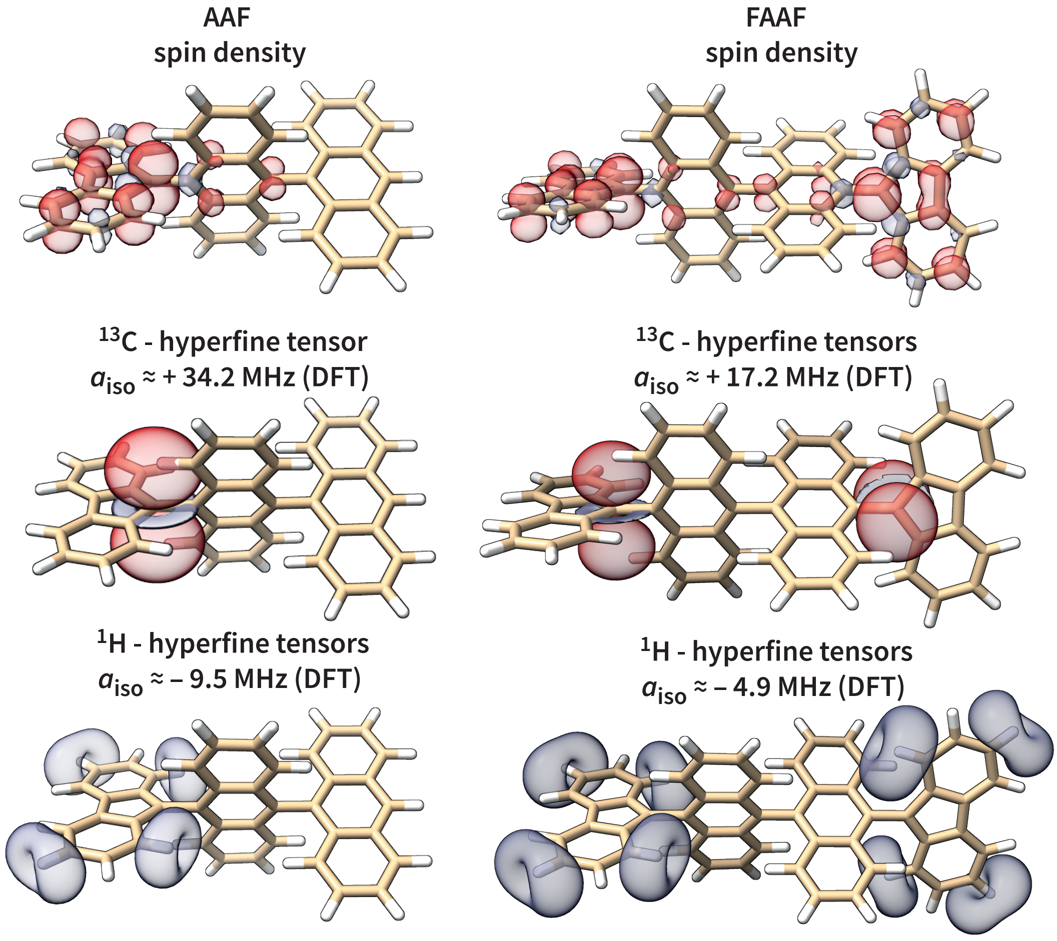

Figure S31: Summary of DFT results for the **AAF** and **FAAF** systems. For **FAAF** a multiplicity of three was used (triplet). The calculations were perfomed using the ORCA software.[11] The molecular geometry was initially optimised using the BP86 functional in conjunction with the def2-SVP basis set. Numerical frequency calculations were performed to ensure convergence to the minimum energy conformation. The spin densities and hyperfine couplings were then evaluated using the EPR-II basis set, which is optimised for EPR parameter predictions. A selection of ORCA outputs containing further calculation details are provided in the next subsection. The top two diagrams show the spin densities with positive values indicated by the red surfaces and negative values by the blue surfaces. The spin densities appear localised mostly on the fluorenyl fragment(s). The bottom four diagrams are the spherical harmonic representations of the hyperfine tensors corresponding to the labelled ^{13}C nuclei and the ^{1}H nuclei with the largest couplings. The ^{13}C coupling is predicted to be strongly axially anisotropic as can be inferred from the similarity of the shape of the hyperfine tensor with the Y_{20} spherical harmonic. The C_∞ axis of the tensor is perpendicular to the plane of the fluorenyl fragment, consistent with the local p_z symmetry of the wavefunction near the ^{13}C nuclei. By contrast, the proton hyperfine tensors are relatively more isotropic, consistent with the local s-character of the wavefunction near the ^{1}H nuclei. The average Fermi contact contribution to the hyperfine couplings (a_{iso}) is also displayed on top of each diagram. The diagrams were generated using the UCSF ChimeraX sofware combined with a home-written Python script for the spherical harmonic decomposition of the hyperfine tensors.[12]

6.2 ORCA input files and all hyperfine tensors

6.2.1 AAF

```
================================================================================
                                       INPUT FILE
================================================================================
|  1> !UKS BP86 EPR-II AUTOAUX
|  2> %PAL NPROCS 48 END
|  3> * xyz 0 2
|  4>   C   -2.82823398259640      2.14885203034831     -0.72228151370411
|  5>   C   -2.81553255615178      2.05066467213778      0.74273214739897
|  6>   C   -2.84496013212941      0.80677859321704     -1.24673183424279
|  7>   C   -2.82393179658647     -0.13309960032317     -0.13922447610647
|  8>   C   -2.80158989653256      0.65058162857840      1.08375979227781
|  9>   C   -2.82834626036447      3.02266706571522      1.74964773917419
| 10>   C   -2.82906587933053      2.61234720165702      3.10018033155282
| 11>   C   -2.82380160504052      1.24219656189073      3.43888741691479
| 12>   C   -2.81343962623870      0.25470908508833      2.43983654745671
| 13>   C   -2.81355259682203      3.24580014171517     -1.59137953914534
| 14>   C   -2.81366491950184      3.01795610715431     -2.98441334203314
| 15>   C   -2.82145086773216      1.70501070872493     -3.50240027803054
| 16>   C   -2.83361084839556      0.59373196181525     -2.64329266573833
| 17>   H   -2.79745835194059      4.27530580750722     -1.20027720182294
| 18>   H   -2.80286382287448      3.87529370188949     -3.67518889986453
| 19>   H   -2.81568958489545      1.55090653037835     -4.59251798845320
| 20>   H   -2.83548651887830     -0.42920325220349     -3.04986035714776
| 21>   H   -2.84247254205298      4.09488075151836      1.49815044147414
| 22>   H   -2.83844365051294      3.37033789131189      3.89871410702881
| 23>   H   -2.82983910320435      0.94433826678463      4.49879692636352
| 24>   H   -2.81263527340878     -0.81266645277912      2.70911666364975
| 25>   C   -2.82576695390378     -1.60529940628696     -0.23842610640641
| 26>   C   -3.94960055206658     -2.28930036506470     -0.80012765241486
| 27>   C   -3.94395497106752     -3.74080313174497     -0.90563348998469
| 28>   C   -2.83097812650563     -4.48891319103270     -0.42923748686558
| 29>   C   -1.71497700797233     -3.81388858465875      0.14002597982173
| 30>   C   -1.70420838895376     -2.36124668510543      0.22741406919782
| 31>   C   -0.52589262082336     -1.72400455848806      0.74625158522420
| 32>   C   0.55667397303468     -2.45931615448690      1.19391770264490
| 33>   C   0.53096504452100     -3.88485010430524      1.13955153075817
| 34>   C   -0.57072117799021     -4.53887154479831      0.62095971034394
| 35>   C   -5.09020962526169     -4.39190339998594     -1.47848510937685
| 36>   C   -6.18940920561187     -3.67117395355497     -1.90602158858248
| 37>   C   -6.21057199034896     -2.25095222727102     -1.77069275956336
| 38>   C   -5.12583630978847     -1.58507195535957     -1.22945666442483
| 39>   H   -7.05559395478167     -4.19397982703624     -2.34029859970965
| 40>   H   -7.09773356015010     -1.68294239096540     -2.09096544722528
| 41>   H   -5.15665706464654     -0.49258600191874     -1.11409691366023
| 42>   H   -5.07961177818327     -5.48807688845193     -1.56499264709006
| 43>   H   -0.49180064048635     -0.62597962174598      0.77716325255036
| 44>   H   1.44550247696568     -1.94172723439850      1.58679441230511
| 45>   H   1.39559945048085     -4.46349185511129      1.49979523804323
| 46>   H   -0.58516907595162     -5.63678493256551      0.56169834664930
| 47>   C   -2.83572234504902     -5.98752167037705     -0.52340571685209
| 48>   C   -3.36290882463428     -6.76435001383592      0.54830089897520
| 49>   C   -3.36429210168352     -8.21752790143560      0.45393201688835
| 50>   C   -2.84401573540064     -8.83528382033725     -0.69978078434362
| 51>   C   -2.32060930609588     -8.08294427366028     -1.76907462626878
| 52>   C   -2.31394845109575     -6.62921177636392     -1.68350547127568
| 53>   C   -1.77226008089466     -5.89044952558507     -2.79063188473473
| 54>   C   -1.27159241166711     -6.53581031031178     -3.90683370951744
| 55>   C   -1.28006064868426     -7.96316377994498     -3.98810843385092
| 56>   C   -1.79111991431428     -8.71280742466728     -2.94579308114204
| 57>   C   -3.89867649792983     -8.98459286948395      1.54375951750343
| 58>   C   -4.40762353430473     -8.36622151178475      2.66985452202664
| 59>   C   -4.40850762815283     -6.93970520165610      2.76499507707522
```

```
| 60>   C   -3.90243918862081     -6.16448122799748      1.73736898177212
| 61>   H   -0.86170351656140     -5.94660177747204     -4.74181429351606
| 62>   H   -0.87757373769784     -8.46112197402548     -4.88382270516985
| 63>   H   -1.76087936509417     -4.79194618792477     -2.73661311773081
| 64>   H   -1.80143668873134     -9.81324871297569     -2.99943332546240
| 65>   H   -2.84673720910942     -9.93553827812934     -0.76760323089854
| 66>   H   -4.81678912340757     -6.45569685126561      3.66577130677802
| 67>   H   -3.90757708680446     -5.06767972457992      1.81933027408076
| 68>   H   -3.89402886709243    -10.08332109628519      1.46167472342223
| 69>   H   -4.81417586229189     -8.96861947768993      3.49695168300416
| 70> *
| 71> %EPRNMR
| 72>         NUCLEI    = ALL H {AISO, ADIP}
| 73>         NUCLEI    = ALL C {AISO, ADIP}
| 74>
| 75> END
| 76>

-----------------------------------------
ELECTRIC AND MAGNETIC HYPERFINE STRUCTURE
-----------------------------------------

 -----------------------------------------------------------
 Nucleus  13H : A:ISTP=    1 I=  0.5 P=533.5514 MHz/au**3
                Q:ISTP=    2 I=  1.0 Q=  0.0029 barn
 -----------------------------------------------------------
 Raw HFC matrix (all values in MHz):
 -----------------------------------
              -0.5724              0.0261              0.0144
               0.0261              1.7112             -0.1763
               0.0144             -0.1763              1.8121

 A(FC)         0.9836              0.9836              0.9836
 A(SD)        -1.5565              0.5951              0.9614
            ----------          ----------          ----------
 A(Tot)       -0.5729              1.5787              1.9450    A(iso)=    0.9836
 Orientation:
  X          0.9999043           0.0137333          -0.0016900
  Y         -0.0119814           0.7982458          -0.6022126
  Z         -0.0069214           0.6021752           0.7983340

Notes:  (1) The A matrix conforms to the "SAI" spin Hamiltonian convention.
        (2) Tensor is right-handed.

 -----------------------------------------------------------
 Nucleus  14H : A:ISTP=    1 I=  0.5 P=533.5514 MHz/au**3
                Q:ISTP=    2 I=  1.0 Q=  0.0029 barn
 -----------------------------------------------------------
 Raw HFC matrix (all values in MHz):
 -----------------------------------
             -10.2218              0.0086             -0.0397
               0.0086             -8.7377             -5.7898
              -0.0397             -5.7898            -10.0648

 A(FC)        -9.6747             -9.6747             -9.6747
 A(SD)         6.1014             -0.5471             -5.5543
            ----------          ----------          ----------
 A(Tot)       -3.5733            -10.2218            -15.2291    A(iso)=   -9.6747
 Orientation:
  X          0.0049400           0.9999764          -0.0047827
  Y          0.7462624          -0.0068702          -0.6656165
  Z         -0.6656336          -0.0002810          -0.7462787

Notes:  (1) The A matrix conforms to the "SAI" spin Hamiltonian convention.
        (2) Tensor is right-handed.
```

```
 ----------------------------------------------------------
 Nucleus  15H : A:ISTP=    1 I=  0.5 P=533.5514 MHz/au**3
                Q:ISTP=    2 I=  1.0 Q=  0.0029 barn
 ----------------------------------------------------------
 Raw HFC matrix (all values in MHz):
 ----------------------------------
                0.4379             0.0313            -0.0046
                0.0313             2.4532            -0.6595
               -0.0046            -0.6595             2.4415

 A(FC)          1.7775             1.7775             1.7775
 A(SD)         -1.3401             0.0105             1.3296
            ----------         ----------         ----------
 A(Tot)         0.4374             1.7881             3.1071    A(iso)=    1.7775
 Orientation:
  X          0.9998583          0.0138835         -0.0095223
  Y         -0.0165359          0.7036978         -0.7103069
  Z         -0.0031607          0.7103637          0.7038277

Notes:  (1) The A matrix conforms to the "SAI" spin Hamiltonian convention.
        (2) Tensor is right-handed.

 ----------------------------------------------------------
 Nucleus  16H : A:ISTP=    1 I=  0.5 P=533.5514 MHz/au**3
                Q:ISTP=    2 I=  1.0 Q=  0.0029 barn
 ----------------------------------------------------------
 Raw HFC matrix (all values in MHz):
 ----------------------------------
              -10.7572            -0.0657             0.0676
               -0.0657            -6.2391             3.4567
                0.0676             3.4567           -10.8355

 A(FC)         -9.2772            -9.2772            -9.2772
 A(SD)          4.8910            -1.4758            -3.4153
            ----------         ----------         ----------
 A(Tot)        -4.3862           -10.7530           -12.6925    A(iso)=   -9.2772
 Orientation:
  X          0.0040768          0.9988967         -0.0467839
  Y         -0.8813903         -0.0185104         -0.4720260
  Z         -0.4723712          0.0431593          0.8803424

Notes:  (1) The A matrix conforms to the "SAI" spin Hamiltonian convention.
        (2) Tensor is right-handed.

 ----------------------------------------------------------
 Nucleus  17H : A:ISTP=    1 I=  0.5 P=533.5514 MHz/au**3
                Q:ISTP=    2 I=  1.0 Q=  0.0029 barn
 ----------------------------------------------------------
 Raw HFC matrix (all values in MHz):
 ----------------------------------
               -0.5725            -0.0238             0.0111
               -0.0238             1.6673             0.1583
                0.0111             0.1583             1.8581

 A(FC)          0.9843             0.9843             0.9843
 A(SD)         -1.5571             0.5939             0.9632
            ----------         ----------         ----------
 A(Tot)        -0.5728             1.5782             1.9475    A(iso)=    0.9843
 Orientation:
  X          0.9999257         -0.0121663         -0.0007946
  Y          0.0109833          0.8705663          0.4919285
  Z         -0.0052932         -0.4919007          0.8706352

Notes:  (1) The A matrix conforms to the "SAI" spin Hamiltonian convention.
        (2) Tensor is right-handed.
```

```
 ------------------------------------------------------------
 Nucleus  18H : A:ISTP=    1 I=  0.5 P=533.5514 MHz/au**3
                Q:ISTP=    2 I=  1.0 Q=  0.0029 barn
 ------------------------------------------------------------
 Raw HFC matrix (all values in MHz):
 -----------------------------------
              -10.2217            -0.0037             -0.0290
               -0.0037           -10.2855              5.7597
               -0.0290             5.7597             -8.5170

 A(FC)         -9.6747            -9.6747             -9.6747
 A(SD)          6.1008            -0.5470             -5.5538
            ----------         ----------          ----------
 A(Tot)        -3.5740           -10.2217            -15.2285    A(iso)=   -9.6747
 Orientation:
  X         -0.0036760          0.9999881          -0.0032052
  Y          0.6512435          0.0048263           0.7588535
  Z          0.7588599          0.0007022          -0.6512535

Notes:  (1) The A matrix conforms to the "SAI" spin Hamiltonian convention.
        (2) Tensor is right-handed.

 ------------------------------------------------------------
 Nucleus  19H : A:ISTP=    1 I=  0.5 P=533.5514 MHz/au**3
                Q:ISTP=    2 I=  1.0 Q=  0.0029 barn
 ------------------------------------------------------------
 Raw HFC matrix (all values in MHz):
 -----------------------------------
                0.4362            -0.0267             -0.0080
               -0.0267             2.2770              0.6387
               -0.0080             0.6387              2.6149

 A(FC)          1.7760             1.7760              1.7760
 A(SD)         -1.3402             0.0094              1.3308
            ----------         ----------          ----------
 A(Tot)         0.4358             1.7854              3.1068    A(iso)=    1.7760
 Orientation:
  X          0.9998910         -0.0120740          -0.0084931
  Y          0.0147481          0.7921877           0.6100992
  Z         -0.0006382         -0.6101580           0.7922795

Notes:  (1) The A matrix conforms to the "SAI" spin Hamiltonian convention.
        (2) Tensor is right-handed.

 ------------------------------------------------------------
 Nucleus  20H : A:ISTP=    1 I=  0.5 P=533.5514 MHz/au**3
                Q:ISTP=    2 I=  1.0 Q=  0.0029 barn
 ------------------------------------------------------------
 Raw HFC matrix (all values in MHz):
 -----------------------------------
              -10.7535             0.0600              0.0719
                0.0600            -5.4147             -2.7467
                0.0719            -2.7467            -11.6613

 A(FC)         -9.2765            -9.2765             -9.2765
 A(SD)          4.8978            -1.4731             -3.4247
            ----------         ----------          ----------
 A(Tot)        -4.3786           -10.7496            -12.7012    A(iso)=   -9.2765
 Orientation:
  X         -0.0048315          0.9989573          -0.0453977
  Y         -0.9356702          0.0115025           0.3526883
  Z          0.3528428          0.0441813           0.9346390

Notes:  (1) The A matrix conforms to the "SAI" spin Hamiltonian convention.
        (2) Tensor is right-handed.
```

```
 ----------------------------------------------------------
 Nucleus  35H : A:ISTP=    1 I=  0.5 P=533.5514 MHz/au**3
                Q:ISTP=    2 I=  1.0 Q=  0.0029 barn
 ----------------------------------------------------------
 Raw HFC matrix (all values in MHz):
 -----------------------------------
                0.5006                0.1123                0.1655
                0.1123                0.6579                0.0916
                0.1655                0.0916                0.1949

 A(FC)          0.4511                0.4511                0.4511
 A(SD)         -0.3317                0.0200                0.3117
            ----------            ----------            ----------
 A(Tot)         0.1195                0.4711                0.7628    A(iso)=    0.4511
 Orientation:
  X          -0.3774882             0.7645732             0.5224274
  Y          -0.0782537            -0.5884882             0.8047099
  Z           0.9227020             0.2628866             0.2819781

Notes:  (1) The A matrix conforms to the "SAI" spin Hamiltonian convention.
        (2) Tensor is right-handed.

 ----------------------------------------------------------
 Nucleus  36H : A:ISTP=    1 I=  0.5 P=533.5514 MHz/au**3
                Q:ISTP=    2 I=  1.0 Q=  0.0029 barn
 ----------------------------------------------------------
 Raw HFC matrix (all values in MHz):
 -----------------------------------
                0.1295                0.0750                0.5115
                0.0750               -1.0080               -0.1026
                0.5115               -0.1026               -0.7380

 A(FC)         -0.5388               -0.5388               -0.5388
 A(SD)          0.9057               -0.3271               -0.5786
            ----------            ----------            ----------
 A(Tot)         0.3669               -0.8659               -1.1174    A(iso)=   -0.5388
 Orientation:
  X           0.9079614            -0.3042632             0.2881494
  Y           0.0183188            -0.6581429            -0.7526702
  Z           0.4186533             0.6886740            -0.5919946

Notes:  (1) The A matrix conforms to the "SAI" spin Hamiltonian convention.
        (2) Tensor is right-handed.

 ----------------------------------------------------------
 Nucleus  37H : A:ISTP=    1 I=  0.5 P=533.5514 MHz/au**3
                Q:ISTP=    2 I=  1.0 Q=  0.0029 barn
 ----------------------------------------------------------
 Raw HFC matrix (all values in MHz):
 -----------------------------------
                3.4608                1.4860                3.0608
                1.4860               -1.9998                0.8238
                3.0608                0.8238               -0.6693

 A(FC)          0.2639                0.2639                0.2639
 A(SD)         -2.5517               -2.6585                5.2102
            ----------            ----------            ----------
 A(Tot)        -2.2878               -2.3946                5.4741    A(iso)=    0.2639
 Orientation:
  X           0.5064146             0.0537470            -0.8606135
  Y          -0.2774520            -0.9348232            -0.2216438
  Z          -0.8164341             0.3510226            -0.4584959

Notes:  (1) The A matrix conforms to the "SAI" spin Hamiltonian convention.
        (2) Tensor is right-handed.
```

```
 -----------------------------------------------------------
 Nucleus  38H : A:ISTP=    1 I=  0.5 P=533.5514 MHz/au**3
                Q:ISTP=    2 I=  1.0 Q=  0.0029 barn
 -----------------------------------------------------------
 Raw HFC matrix (all values in MHz):
 -----------------------------------
               -0.7280              0.3578              0.1250
                0.3578              0.2693              0.2191
                0.1250              0.2191             -0.6954

 A(FC)         -0.3847             -0.3847             -0.3847
 A(SD)          0.8230             -0.3423             -0.4806
            ----------          ----------          ----------
 A(Tot)         0.4382             -0.7270             -0.8654    A(iso)=   -0.3847
 Orientation:
  X          -0.3074215           0.3202443          -0.8960667
  Y          -0.9273960          -0.3117530           0.2067529
  Z          -0.2131401           0.8945690           0.3928329

Notes:  (1) The A matrix conforms to the "SAI" spin Hamiltonian convention.
        (2) Tensor is right-handed.

 -----------------------------------------------------------
 Nucleus  39H : A:ISTP=    1 I=  0.5 P=533.5514 MHz/au**3
                Q:ISTP=    2 I=  1.0 Q=  0.0029 barn
 -----------------------------------------------------------
 Raw HFC matrix (all values in MHz):
 -----------------------------------
                3.4596             -1.8992              2.8307
               -1.8992             -1.7432             -0.9799
                2.8307             -0.9799             -0.9151

 A(FC)          0.2671              0.2671              0.2671
 A(SD)         -2.5535             -2.6609              5.2144
            ----------          ----------          ----------
 A(Tot)        -2.2864             -2.3938              5.4815    A(iso)=    0.2671
 Orientation:
  X           0.5079915          -0.0469289          -0.8600827
  Y           0.3996489          -0.8716924           0.2836074
  Z          -0.7630369          -0.4878012          -0.4240573

Notes:  (1) The A matrix conforms to the "SAI" spin Hamiltonian convention.
        (2) Tensor is right-handed.

 -----------------------------------------------------------
 Nucleus  40H : A:ISTP=    1 I=  0.5 P=533.5514 MHz/au**3
                Q:ISTP=    2 I=  1.0 Q=  0.0029 barn
 -----------------------------------------------------------
 Raw HFC matrix (all values in MHz):
 -----------------------------------
                0.1281             -0.1453              0.4973
               -0.1453             -1.0315              0.0623
                0.4973              0.0623             -0.7172

 A(FC)         -0.5402             -0.5402             -0.5402
 A(SD)          0.9063             -0.3272             -0.5792
            ----------          ----------          ----------
 A(Tot)         0.3661             -0.8674             -1.1194    A(iso)=   -0.5402
 Orientation:
  X          -0.9078460           0.3030077          -0.2898310
  Y           0.0759754          -0.5609056          -0.8243862
  Z          -0.4123632          -0.7704357           0.4861948

Notes:  (1) The A matrix conforms to the "SAI" spin Hamiltonian convention.
        (2) Tensor is right-handed.
```

```
 ----------------------------------------------------------
 Nucleus  41H : A:ISTP=    1 I=  0.5 P=533.5514 MHz/au**3
                Q:ISTP=    2 I=  1.0 Q=  0.0029 barn
 ----------------------------------------------------------
 Raw HFC matrix (all values in MHz):
 ----------------------------------
               0.4997              -0.1328              0.1490
              -0.1328               0.6735             -0.0280
               0.1490              -0.0280              0.1779

 A(FC)         0.4504               0.4504              0.4504
 A(SD)        -0.3318               0.0201              0.3117
            ----------           ----------           ----------
 A(Tot)        0.1185               0.4705              0.7621    A(iso)=    0.4504
 Orientation:
  X       -0.3769249           -0.7657829            0.5210606
  Y       -0.0434743           -0.5473058           -0.8358028
  Z        0.9252230           -0.3376876            0.1730013

Notes:  (1) The A matrix conforms to the "SAI" spin Hamiltonian convention.
        (2) Tensor is right-handed.

 ----------------------------------------------------------
 Nucleus  42H : A:ISTP=    1 I=  0.5 P=533.5514 MHz/au**3
                Q:ISTP=    2 I=  1.0 Q=  0.0029 barn
 ----------------------------------------------------------
 Raw HFC matrix (all values in MHz):
 ----------------------------------
              -0.7340              -0.3675              0.0766
              -0.3675               0.3110             -0.0851
               0.0766              -0.0851             -0.7385

 A(FC)        -0.3872              -0.3872             -0.3872
 A(SD)         0.8237              -0.3422             -0.4815
            ----------           ----------           ----------
 A(Tot)        0.4366              -0.7294             -0.8687    A(iso)=   -0.3872
 Orientation:
  X       -0.3036153           -0.3211385           -0.8970439
  Y        0.9486766           -0.1893305           -0.2533113
  Z       -0.0884897           -0.9279138            0.3621403

Notes:  (1) The A matrix conforms to the "SAI" spin Hamiltonian convention.
        (2) Tensor is right-handed.

 ----------------------------------------------------------
 Nucleus  57H : A:ISTP=    1 I=  0.5 P=533.5514 MHz/au**3
                Q:ISTP=    2 I=  1.0 Q=  0.0029 barn
 ----------------------------------------------------------
 Raw HFC matrix (all values in MHz):
 ----------------------------------
              -0.0921              -0.0868             -0.0901
              -0.0868               0.1162              0.1877
              -0.0901               0.1877              0.0745

 A(FC)         0.0329               0.0329              0.0329
 A(SD)        -0.1243              -0.1646              0.2890
            ----------           ----------           ----------
 A(Tot)       -0.0915              -0.1318              0.3218    A(iso)=    0.0329
 Orientation:
  X        0.2144651            0.9330956            0.2886821
  Y        0.7027885            0.0578341           -0.7090441
  Z       -0.6783016            0.3549477           -0.6433655

Notes:  (1) The A matrix conforms to the "SAI" spin Hamiltonian convention.
        (2) Tensor is right-handed.
```

```
 -----------------------------------------------------------
 Nucleus  58H : A:ISTP=    1 I=  0.5 P=533.5514 MHz/au**3
                Q:ISTP=    2 I=  1.0 Q=  0.0029 barn
 -----------------------------------------------------------
 Raw HFC matrix (all values in MHz):
 -----------------------------------
               -0.0667             -0.0420             -0.0295
               -0.0420              0.0902              0.0973
               -0.0295              0.0973             -0.0087

 A(FC)          0.0049              0.0049              0.0049
 A(SD)         -0.0724             -0.0841              0.1565
            ----------          ----------          ----------
 A(Tot)        -0.0675             -0.0791              0.1614    A(iso)=    0.0049
 Orientation:
  X          0.2731427           0.9366654           0.2192055
  Y          0.5568268           0.0318695          -0.8300170
  Z         -0.7844342           0.3487726          -0.5128554

Notes:  (1) The A matrix conforms to the "SAI" spin Hamiltonian convention.
        (2) Tensor is right-handed.

 -----------------------------------------------------------
 Nucleus  59H : A:ISTP=    1 I=  0.5 P=533.5514 MHz/au**3
                Q:ISTP=    2 I=  1.0 Q=  0.0029 barn
 -----------------------------------------------------------
 Raw HFC matrix (all values in MHz):
 -----------------------------------
               -0.4597             -0.2109             -0.3808
               -0.2109              0.1746              0.4893
               -0.3808              0.4893              0.2527

 A(FC)         -0.0108             -0.0108             -0.0108
 A(SD)         -0.2394             -0.6142              0.8536
            ----------          ----------          ----------
 A(Tot)        -0.2502             -0.6250              0.8428    A(iso)=   -0.0108
 Orientation:
  X          0.2505918           0.9168647           0.3107456
  Y          0.7818625          -0.0023942          -0.6234463
  Z         -0.5708719           0.3991909          -0.7174621

Notes:  (1) The A matrix conforms to the "SAI" spin Hamiltonian convention.
        (2) Tensor is right-handed.

 -----------------------------------------------------------
 Nucleus  60H : A:ISTP=    1 I=  0.5 P=533.5514 MHz/au**3
                Q:ISTP=    2 I=  1.0 Q=  0.0029 barn
 -----------------------------------------------------------
 Raw HFC matrix (all values in MHz):
 -----------------------------------
               -0.0460             -0.0251             -0.0096
               -0.0251              0.1639              0.0666
               -0.0096              0.0666             -0.0225

 A(FC)          0.0318              0.0318              0.0318
 A(SD)         -0.0753             -0.0812              0.1565
            ----------          ----------          ----------
 A(Tot)        -0.0435             -0.0495              0.1883    A(iso)=    0.0318
 Orientation:
  X          0.2414346           0.9637153           0.1138512
  Y          0.3232250           0.0307614          -0.9458220
  Z         -0.9150054           0.2651538          -0.3040700

Notes:  (1) The A matrix conforms to the "SAI" spin Hamiltonian convention.
        (2) Tensor is right-handed.
```

```
 -----------------------------------------------------------
 Nucleus  61H : A:ISTP=    1 I=  0.5 P=533.5514 MHz/au**3
                Q:ISTP=    2 I=  1.0 Q=  0.0029 barn
 -----------------------------------------------------------
 Raw HFC matrix (all values in MHz):
 -----------------------------------
              -0.1056              0.0006              0.0009
               0.0006              0.1658              0.0172
               0.0009              0.0172             -0.1037

 A(FC)        -0.0145             -0.0145             -0.0145
 A(SD)        -0.0897             -0.0917              0.1814
            ----------          ----------          ----------
 A(Tot)       -0.1042             -0.1062              0.1669    A(iso)=   -0.0145
 Orientation:
  X         -0.5325779           0.8463774          -0.0024652
  Y          0.0550433           0.0317289          -0.9979797
  Z         -0.8445893          -0.5316376          -0.0634856

Notes:  (1) The A matrix conforms to the "SAI" spin Hamiltonian convention.
        (2) Tensor is right-handed.

 -----------------------------------------------------------
 Nucleus  62H : A:ISTP=    1 I=  0.5 P=533.5514 MHz/au**3
                Q:ISTP=    2 I=  1.0 Q=  0.0029 barn
 -----------------------------------------------------------
 Raw HFC matrix (all values in MHz):
 -----------------------------------
              -0.0923              0.0998             -0.0798
               0.0998              0.1633             -0.1767
              -0.0798             -0.1767              0.0267

 A(FC)         0.0325              0.0325              0.0325
 A(SD)        -0.1249             -0.1660              0.2909
            ----------          ----------          ----------
 A(Tot)       -0.0924             -0.1335              0.3235    A(iso)=    0.0325
 Orientation:
  X         -0.2118849           0.9322783           0.2931928
  Y          0.6102176          -0.1081304           0.7848199
  Z          0.7633736           0.3452029          -0.5459814

Notes:  (1) The A matrix conforms to the "SAI" spin Hamiltonian convention.
        (2) Tensor is right-handed.

 -----------------------------------------------------------
 Nucleus  63H : A:ISTP=    1 I=  0.5 P=533.5514 MHz/au**3
                Q:ISTP=    2 I=  1.0 Q=  0.0029 barn
 -----------------------------------------------------------
 Raw HFC matrix (all values in MHz):
 -----------------------------------
              -0.4586              0.2620             -0.3551
               0.2620              0.3018             -0.4788
              -0.3551             -0.4788              0.1251

 A(FC)        -0.0105             -0.0105             -0.0105
 A(SD)        -0.2355             -0.6176              0.8531
            ----------          ----------          ----------
 A(Tot)       -0.2461             -0.6281              0.8426    A(iso)=   -0.0105
 Orientation:
  X          0.2492546           0.9158239          -0.3148631
  Y         -0.7020701          -0.0530657          -0.7101279
  Z         -0.6670606           0.3980586           0.6297457

Notes:  (1) The A matrix conforms to the "SAI" spin Hamiltonian convention.
        (2) Tensor is right-handed.
```

```
 -----------------------------------------------------------
 Nucleus  64H : A:ISTP=    1 I=  0.5 P=533.5514 MHz/au**3
                Q:ISTP=    2 I=  1.0 Q=  0.0029 barn
 -----------------------------------------------------------
 Raw HFC matrix (all values in MHz):
 -----------------------------------
               -0.0456              0.0273             -0.0065
                0.0273              0.1780             -0.0411
               -0.0065             -0.0411             -0.0360

 A(FC)          0.0321              0.0321              0.0321
 A(SD)         -0.0754             -0.0815              0.1568
            ----------          ----------          ----------
 A(Tot)        -0.0432             -0.0493              0.1890    A(iso)=    0.0321
 Orientation:
  X          -0.2388693           0.9637504           0.1188556
  Y           0.2056815          -0.0694064           0.9761546
  Z           0.9490187           0.2576198          -0.1816466

Notes:  (1) The A matrix conforms to the "SAI" spin Hamiltonian convention.
        (2) Tensor is right-handed.

 -----------------------------------------------------------
 Nucleus  65H : A:ISTP=    1 I=  0.5 P=533.5514 MHz/au**3
                Q:ISTP=    2 I=  1.0 Q=  0.0029 barn
 -----------------------------------------------------------
 Raw HFC matrix (all values in MHz):
 -----------------------------------
               -0.0665              0.0466             -0.0245
                0.0466              0.1132             -0.0818
               -0.0245             -0.0818             -0.0318

 A(FC)          0.0050              0.0050              0.0050
 A(SD)         -0.0726             -0.0845              0.1571
            ----------          ----------          ----------
 A(Tot)        -0.0677             -0.0795              0.1621    A(iso)=    0.0050
 Orientation:
  X          -0.2722148           0.9357954           0.2240227
  Y           0.4537529          -0.0804653           0.8874873
  Z           0.8485325           0.3432381          -0.4027160

Notes:  (1) The A matrix conforms to the "SAI" spin Hamiltonian convention.
        (2) Tensor is right-handed.

 -----------------------------------------------------------
 Nucleus   0C : A:ISTP=   13 I=  0.5 P=134.1903 MHz/au**3
                Q:ISTP=   13 I=  0.5 Q=  0.0000 barn
 -----------------------------------------------------------
 Raw HFC matrix (all values in MHz):
 -----------------------------------
               19.1365             -0.6040             -0.3463
               -0.6040             -3.8993             -0.3688
               -0.3463             -0.3688             -4.0434

 A(FC)          3.7312              3.7312              3.7312
 A(SD)         -7.3294             -8.0965             15.4259
            ----------          ----------          ----------
 A(Tot)        -3.5982             -4.3653             19.1572    A(iso)=    3.7312
 Orientation:
  X          -0.0105604          -0.0277954           0.9995578
  Y          -0.7660145          -0.6422992          -0.0259539
  Z           0.6427366          -0.7659499          -0.0145087

Notes:  (1) The A matrix conforms to the "SAI" spin Hamiltonian convention.
        (2) Tensor is right-handed.
```

```
 -----------------------------------------------------------
 Nucleus   1C : A:ISTP=   13 I=  0.5 P=134.1903 MHz/au**3
               Q:ISTP=   13 I=  0.5 Q=  0.0000 barn
 -----------------------------------------------------------
 Raw HFC matrix (all values in MHz):
 -----------------------------------
               19.1288              0.6045              -0.2659
                0.6045             -4.0000               0.3751
               -0.2659              0.3751              -3.9419

 A(FC)          3.7290              3.7290               3.7290
 A(SD)         -7.3257             -8.0927              15.4184
            ----------          ----------          ----------
 A(Tot)        -3.5967             -4.3638              19.1474    A(iso)=    3.7290
 Orientation:
  X          0.0092676           0.0266309          -0.9996024
  Y         -0.6735335          -0.7387020          -0.0259247
  Z         -0.7390986           0.6735059           0.0110908

Notes:  (1) The A matrix conforms to the "SAI" spin Hamiltonian convention.
        (2) Tensor is right-handed.

 -----------------------------------------------------------
 Nucleus   2C : A:ISTP=   13 I=  0.5 P=134.1903 MHz/au**3
               Q:ISTP=   13 I=  0.5 Q=  0.0000 barn
 -----------------------------------------------------------
 Raw HFC matrix (all values in MHz):
 -----------------------------------
              -29.5469             -0.3846               0.3082
               -0.3846            -19.8062              -1.0043
                0.3082             -1.0043             -18.0028

 A(FC)        -22.4520            -22.4520             -22.4520
 A(SD)          4.9132              2.2032              -7.1164
            ----------          ----------          ----------
 A(Tot)       -17.5388            -20.2488             -29.5684    A(iso)=  -22.4520
 Orientation:
  X          0.0365239          -0.0240819          -0.9990426
  Y         -0.4098824           0.9113895          -0.0369538
  Z          0.9114068           0.4108397           0.0234168

Notes:  (1) The A matrix conforms to the "SAI" spin Hamiltonian convention.
        (2) Tensor is right-handed.

 -----------------------------------------------------------
 Nucleus   3C : A:ISTP=   13 I=  0.5 P=134.1903 MHz/au**3
               Q:ISTP=   13 I=  0.5 Q=  0.0000 barn
 -----------------------------------------------------------
 Raw HFC matrix (all values in MHz):
 -----------------------------------
              112.2512              0.0956              -3.2545
                0.0956             -5.0617              -0.0206
               -3.2545             -0.0206              -4.7100

 A(FC)         34.1598             34.1598              34.1598
 A(SD)        -38.9591            -39.2229              78.1819
            ----------          ----------          ----------
 A(Tot)        -4.7993             -5.0631             112.3417    A(iso)=   34.1598
 Orientation:
  X          0.0277845          -0.0010817           0.9996134
  Y         -0.0682894          -0.9976652           0.0008185
  Z          0.9972786          -0.0682857          -0.0277935

Notes:  (1) The A matrix conforms to the "SAI" spin Hamiltonian convention.
        (2) Tensor is right-handed.
```

```
 -----------------------------------------------------------
 Nucleus   4C : A:ISTP=   13 I=  0.5 P=134.1903 MHz/au**3
               Q:ISTP=   13 I=  0.5 Q=  0.0000 barn
 -----------------------------------------------------------
 Raw HFC matrix (all values in MHz):
 -----------------------------------
              -29.5471              0.3606              0.3595
                0.3606            -20.0513              0.7318
                0.3595              0.7318            -17.7751

 A(FC)        -22.4578            -22.4578            -22.4578
 A(SD)          4.9143              2.1979             -7.1123
            ----------          ----------          ----------
 A(Tot)       -17.5435            -20.2599            -29.5701    A(iso)=  -22.4578
 Orientation:
  X          0.0372458           0.0261288          -0.9989645
  Y          0.2848575           0.9579058           0.0356756
  Z          0.9578460          -0.2858913           0.0282350

Notes:  (1) The A matrix conforms to the "SAI" spin Hamiltonian convention.
        (2) Tensor is right-handed.

 -----------------------------------------------------------
 Nucleus   5C : A:ISTP=   13 I=  0.5 P=134.1903 MHz/au**3
               Q:ISTP=   13 I=  0.5 Q=  0.0000 barn
 -----------------------------------------------------------
 Raw HFC matrix (all values in MHz):
 -----------------------------------
              -10.5320              0.0003              0.0105
                0.0003             -6.7039              0.2984
                0.0105              0.2984             -5.8372

 A(FC)         -7.6910             -7.6910             -7.6910
 A(SD)          1.9466              0.8944             -2.8410
            ----------          ----------          ----------
 A(Tot)        -5.7444             -6.7967            -10.5320    A(iso)=   -7.6910
 Orientation:
  X          0.0021164          -0.0007576          -0.9999975
  Y          0.2969506           0.9548928          -0.0000949
  Z          0.9548905          -0.2969497           0.0022459

Notes:  (1) The A matrix conforms to the "SAI" spin Hamiltonian convention.
        (2) Tensor is right-handed.

 -----------------------------------------------------------
 Nucleus   6C : A:ISTP=   13 I=  0.5 P=134.1903 MHz/au**3
               Q:ISTP=   13 I=  0.5 Q=  0.0000 barn
 -----------------------------------------------------------
 Raw HFC matrix (all values in MHz):
 -----------------------------------
               28.3147              0.2489              0.1559
                0.2489             -2.0716              0.4118
                0.1559              0.4118             -1.8821

 A(FC)          8.1203              8.1203              8.1203
 A(SD)         -9.6772            -10.5200             20.1972
            ----------          ----------          ----------
 A(Tot)        -1.5569             -2.3997             28.3176    A(iso)=    8.1203
 Orientation:
  X         -0.0092703          -0.0031878          -0.9999519
  Y          0.6219271           0.7830316          -0.0082619
  Z          0.7830203          -0.6219738          -0.0052763

Notes:  (1) The A matrix conforms to the "SAI" spin Hamiltonian convention.
        (2) Tensor is right-handed.
```

```
 -----------------------------------------------------------
 Nucleus   7C : A:ISTP=   13 I=  0.5 P=134.1903 MHz/au**3
                Q:ISTP=   13 I=  0.5 Q=  0.0000 barn
 -----------------------------------------------------------
 Raw HFC matrix (all values in MHz):
 -----------------------------------
              -13.8892            -0.0640            -0.0837
               -0.0640            -6.4723             0.3204
               -0.0837             0.3204            -6.9422

 A(FC)         -9.1012            -9.1012            -9.1012
 A(SD)          2.7925             1.9970            -4.7895
            ----------         ----------         ----------
 A(Tot)        -6.3088            -7.1042           -13.8907    A(iso)=   -9.1012
 Orientation:
  X         -0.0125275         -0.0067345          0.9998988
  Y          0.8916226         -0.4527065          0.0081219
  Z          0.4526060          0.8916341          0.0116759

Notes:  (1) The A matrix conforms to the "SAI" spin Hamiltonian convention.
        (2) Tensor is right-handed.

 -----------------------------------------------------------
 Nucleus   8C : A:ISTP=   13 I=  0.5 P=134.1903 MHz/au**3
                Q:ISTP=   13 I=  0.5 Q=  0.0000 barn
 -----------------------------------------------------------
 Raw HFC matrix (all values in MHz):
 -----------------------------------
               27.2502            -0.1525             0.6121
               -0.1525            -1.6199             0.0583
                0.6121             0.0583            -1.2520

 A(FC)          8.1261             8.1261             8.1261
 A(SD)         -9.3809            -9.7571            19.1380
            ----------         ----------         ----------
 A(Tot)        -1.2548            -1.6311            27.2641    A(iso)=    8.1261
 Orientation:
  X         -0.0202824          0.0087213         -0.9997563
  Y          0.1660192          0.9861086          0.0052341
  Z          0.9859139         -0.1658726         -0.0214486

Notes:  (1) The A matrix conforms to the "SAI" spin Hamiltonian convention.
        (2) Tensor is right-handed.

 -----------------------------------------------------------
 Nucleus   9C : A:ISTP=   13 I=  0.5 P=134.1903 MHz/au**3
                Q:ISTP=   13 I=  0.5 Q=  0.0000 barn
 -----------------------------------------------------------
 Raw HFC matrix (all values in MHz):
 -----------------------------------
              -10.5348             0.0055             0.0109
                0.0055            -6.6124            -0.4017
                0.0109            -0.4017            -5.9340

 A(FC)         -7.6937            -7.6937            -7.6937
 A(SD)          1.9463             0.8948            -2.8411
            ----------         ----------         ----------
 A(Tot)        -5.7474            -6.7989           -10.5348    A(iso)=   -7.6937
 Orientation:
  X          0.0015756          0.0025540         -0.9999955
  Y         -0.4212156          0.9069590          0.0016527
  Z          0.9069592          0.4212111          0.0025048

Notes:  (1) The A matrix conforms to the "SAI" spin Hamiltonian convention.
        (2) Tensor is right-handed.
```

```
 ------------------------------------------------------------
 Nucleus  10C : A:ISTP=   13 I=  0.5 P=134.1903 MHz/au**3
                Q:ISTP=   13 I=  0.5 Q=  0.0000 barn
 ------------------------------------------------------------
 Raw HFC matrix (all values in MHz):
 -----------------------------------
               28.3183              -0.3278               0.1099
               -0.3278              -1.9566              -0.4226
                0.1099              -0.4226              -1.9919

 A(FC)          8.1233               8.1233               8.1233
 A(SD)         -9.6779             -10.5212              20.1990
            ----------           ----------           ----------
 A(Tot)        -1.5546              -2.3979              28.3223    A(iso)=    8.1233
 Orientation:
  X          0.0104564            0.0048222            0.9999337
  Y          0.7204054            0.6934679           -0.0108776
  Z         -0.6934744            0.7204714            0.0037772

Notes:  (1) The A matrix conforms to the "SAI" spin Hamiltonian convention.
        (2) Tensor is right-handed.

 ------------------------------------------------------------
 Nucleus  11C : A:ISTP=   13 I=  0.5 P=134.1903 MHz/au**3
                Q:ISTP=   13 I=  0.5 Q=  0.0000 barn
 ------------------------------------------------------------
 Raw HFC matrix (all values in MHz):
 -----------------------------------
              -13.8904               0.0865              -0.0740
                0.0865              -6.3966              -0.2472
               -0.0740              -0.2472              -7.0174

 A(FC)         -9.1014              -9.1014              -9.1014
 A(SD)          2.7927               1.9979              -4.7906
            ----------           ----------           ----------
 A(Tot)        -6.3087              -7.1035             -13.8921    A(iso)=   -9.1014
 Orientation:
  X          0.0139968           -0.0060711            0.9998836
  Y          0.9436667            0.3307079           -0.0112018
  Z         -0.3306014            0.9437136            0.0103579

Notes:  (1) The A matrix conforms to the "SAI" spin Hamiltonian convention.
        (2) Tensor is right-handed.

 ------------------------------------------------------------
 Nucleus  12C : A:ISTP=   13 I=  0.5 P=134.1903 MHz/au**3
                Q:ISTP=   13 I=  0.5 Q=  0.0000 barn
 ------------------------------------------------------------
 Raw HFC matrix (all values in MHz):
 -----------------------------------
               27.2395               0.0356               0.6305
                0.0356              -1.6016              -0.1058
                0.6305              -0.1058              -1.2753

 A(FC)          8.1209               8.1209               8.1209
 A(SD)         -9.3772              -9.7554              19.1326
            ----------           ----------           ----------
 A(Tot)        -1.2564              -1.6345              27.2535    A(iso)=    8.1209
 Orientation:
  X         -0.0207696           -0.0076165           -0.9997553
  Y         -0.2949427            0.9555143           -0.0011521
  Z          0.9552892            0.2948466           -0.0220921

Notes:  (1) The A matrix conforms to the "SAI" spin Hamiltonian convention.
        (2) Tensor is right-handed.
```

```
 -----------------------------------------------------------
 Nucleus  21C : A:ISTP=   13 I=  0.5 P=134.1903 MHz/au**3
                Q:ISTP=   13 I=  0.5 Q=  0.0000 barn
 -----------------------------------------------------------
 Raw HFC matrix (all values in MHz):
 -----------------------------------
              -18.2962             -0.1442              2.1928
               -0.1442            -16.4089              0.4305
                2.1928              0.4305            -22.8195

 A(FC)        -19.1749            -19.1749            -19.1749
 A(SD)          2.7948              1.7664             -4.5612
             ----------          ----------          ----------
 A(Tot)       -16.3801            -17.4085            -23.7360    A(iso)=  -19.1749
 Orientation:
  X          -0.0020247           0.9272021          -0.3745561
  Y          -0.9977244          -0.0271157          -0.0617308
  Z          -0.0673933           0.3735787           0.9251471

Notes:  (1) The A matrix conforms to the "SAI" spin Hamiltonian convention.
        (2) Tensor is right-handed.

 -----------------------------------------------------------
 Nucleus  22C : A:ISTP=   13 I=  0.5 P=134.1903 MHz/au**3
                Q:ISTP=   13 I=  0.5 Q=  0.0000 barn
 -----------------------------------------------------------
 Raw HFC matrix (all values in MHz):
 -----------------------------------
               16.7455              0.8112              0.7779
                0.8112             17.9745              2.2709
                0.7779              2.2709             23.7823

 A(FC)         19.5008             19.5008             19.5008
 A(SD)         -3.1794             -2.0109              5.1904
             ----------          ----------          ----------
 A(Tot)        16.3214             17.4899             24.6912    A(iso)=   19.5008
 Orientation:
  X           0.8566578           0.5004182           0.1253760
  Y          -0.5115937           0.7927794           0.3313197
  Z           0.0664029          -0.3479692           0.9351514

Notes:  (1) The A matrix conforms to the "SAI" spin Hamiltonian convention.
        (2) Tensor is right-handed.

 -----------------------------------------------------------
 Nucleus  23C : A:ISTP=   13 I=  0.5 P=134.1903 MHz/au**3
                Q:ISTP=   13 I=  0.5 Q=  0.0000 barn
 -----------------------------------------------------------
 Raw HFC matrix (all values in MHz):
 -----------------------------------
               -1.4083              0.0045              0.8726
                0.0045             -0.3995              0.2572
                0.8726              0.2572             -2.6189

 A(FC)         -1.4756             -1.4756             -1.4756
 A(SD)          1.1203              0.4987             -1.6190
             ----------          ----------          ----------
 A(Tot)        -0.3553             -0.9769             -3.0945    A(iso)=   -1.4756
 Orientation:
  X          -0.1412020           0.8777829          -0.4577763
  Y          -0.9760763          -0.2006687          -0.0837082
  Z          -0.1653390           0.4350049           0.8851179

Notes:  (1) The A matrix conforms to the "SAI" spin Hamiltonian convention.
        (2) Tensor is right-handed.
```

```
 -----------------------------------------------------------
 Nucleus  24C : A:ISTP=   13 I=  0.5 P=134.1903 MHz/au**3
                Q:ISTP=   13 I=  0.5 Q=  0.0000 barn
 -----------------------------------------------------------
 Raw HFC matrix (all values in MHz):
 -----------------------------------
                1.9780               0.3238               -5.2002
                0.3238              -0.3518               -0.5688
               -5.2002              -0.5688                8.7958

 A(FC)          3.4740               3.4740                3.4740
 A(SD)         -3.8610              -4.3055                8.1665
             ----------           ----------           ----------
 A(Tot)        -0.3870              -0.8315               11.6405    A(iso)=    3.4740
 Orientation:
  X          -0.0029691            0.8801861           -0.4746194
  Y          -0.9979777           -0.0327449           -0.0544827
  Z          -0.0634963            0.4734978            0.8785033

Notes:  (1) The A matrix conforms to the "SAI" spin Hamiltonian convention.
        (2) Tensor is right-handed.

 -----------------------------------------------------------
 Nucleus  25C : A:ISTP=   13 I=  0.5 P=134.1903 MHz/au**3
                Q:ISTP=   13 I=  0.5 Q=  0.0000 barn
 -----------------------------------------------------------
 Raw HFC matrix (all values in MHz):
 -----------------------------------
               -1.4270              -0.1147                0.8625
               -0.1147              -0.3871                0.0381
                0.8625               0.0381               -2.6685

 A(FC)         -1.4942              -1.4942               -1.4942
 A(SD)          1.1215               0.4974               -1.6189
             ----------           ----------           ----------
 A(Tot)        -0.3727              -0.9968               -3.1131    A(iso)=   -1.4942
 Orientation:
  X           0.1360814            0.8790651           -0.4568659
  Y          -0.9900904            0.1368172           -0.0316540
  Z           0.0346811            0.4566461            0.8889722

Notes:  (1) The A matrix conforms to the "SAI" spin Hamiltonian convention.
        (2) Tensor is right-handed.

 -----------------------------------------------------------
 Nucleus  26C : A:ISTP=   13 I=  0.5 P=134.1903 MHz/au**3
                Q:ISTP=   13 I=  0.5 Q=  0.0000 barn
 -----------------------------------------------------------
 Raw HFC matrix (all values in MHz):
 -----------------------------------
               16.6668              -0.9030                0.6536
               -0.9030              18.6122               -2.9582
                0.6536              -2.9582               22.9913

 A(FC)         19.4234              19.4234               19.4234
 A(SD)         -3.1759              -2.0068                5.1827
             ----------           ----------           ----------
 A(Tot)        16.2475              17.4167               24.6061    A(iso)=   19.4234
 Orientation:
  X           0.8580939           -0.4982256            0.1242821
  Y           0.4956338            0.7403483           -0.4541272
  Z           0.1342457            0.4512822            0.8822259

Notes:  (1) The A matrix conforms to the "SAI" spin Hamiltonian convention.
        (2) Tensor is right-handed.
```

```
 -----------------------------------------------------------
 Nucleus  27C : A:ISTP=   13 I=  0.5 P=134.1903 MHz/au**3
                Q:ISTP=   13 I=  0.5 Q=  0.0000 barn
 -----------------------------------------------------------
 Raw HFC matrix (all values in MHz):
 -----------------------------------
               -0.2372             -0.5824              0.9220
               -0.5824              0.2838              0.1867
                0.9220              0.1867             -1.8889

 A(FC)         -0.6141             -0.6141             -0.6141
 A(SD)          0.4177              1.3339             -1.7516
             ----------          ----------          ----------
 A(Tot)        -0.1964              0.7197             -2.3657    A(iso)=   -0.6141
 Orientation:
  X          0.6530432           0.6245123           0.4283912
  Y          0.6268884          -0.7631489           0.1568907
  Z          0.4249065           0.1660971          -0.8898687

Notes:  (1) The A matrix conforms to the "SAI" spin Hamiltonian convention.
        (2) Tensor is right-handed.

 -----------------------------------------------------------
 Nucleus  28C : A:ISTP=   13 I=  0.5 P=134.1903 MHz/au**3
                Q:ISTP=   13 I=  0.5 Q=  0.0000 barn
 -----------------------------------------------------------
 Raw HFC matrix (all values in MHz):
 -----------------------------------
                0.5910             -0.0613             -0.7559
               -0.0613              0.1303             -0.2649
               -0.7559             -0.2649              2.3386

 A(FC)          1.0199              1.0199              1.0199
 A(SD)         -0.9899             -0.6308              1.6207
             ----------          ----------          ----------
 A(Tot)         0.0300              0.3891              2.6407    A(iso)=    1.0199
 Orientation:
  X         -0.4148521           0.8430896          -0.3421953
  Y         -0.8785770          -0.4689796          -0.0903354
  Z         -0.2366435           0.2631691           0.9352764

Notes:  (1) The A matrix conforms to the "SAI" spin Hamiltonian convention.
        (2) Tensor is right-handed.

 -----------------------------------------------------------
 Nucleus  29C : A:ISTP=   13 I=  0.5 P=134.1903 MHz/au**3
                Q:ISTP=   13 I=  0.5 Q=  0.0000 barn
 -----------------------------------------------------------
 Raw HFC matrix (all values in MHz):
 -----------------------------------
               -0.2563             -0.0996              0.6531
               -0.0996              0.0986             -0.0238
                0.6531             -0.0238             -1.4086

 A(FC)         -0.5221             -0.5221             -0.5221
 A(SD)          0.4857              0.6958             -1.1815
             ----------          ----------          ----------
 A(Tot)        -0.0364              0.1736             -1.7036    A(iso)=   -0.5221
 Orientation:
  X         -0.7283976          -0.5475085           0.4119118
  Y         -0.5969304           0.8022212           0.0107311
  Z         -0.3363198          -0.2380662          -0.9111605

Notes:  (1) The A matrix conforms to the "SAI" spin Hamiltonian convention.
        (2) Tensor is right-handed.
```

```
 -----------------------------------------------------------
 Nucleus  30C : A:ISTP=   13 I=  0.5 P=134.1903 MHz/au**3
               Q:ISTP=   13 I=  0.5 Q=  0.0000 barn
 -----------------------------------------------------------
 Raw HFC matrix (all values in MHz):
 -----------------------------------
                1.0900              -0.0776              -0.4982
               -0.0776               1.1138              -0.3909
               -0.4982              -0.3909               2.8094

 A(FC)          1.6710               1.6710               1.6710
 A(SD)         -0.8493              -0.4908               1.3401
             ----------           ----------           ----------
 A(Tot)         0.8217               1.1802               3.0111    A(iso)=    1.6710
 Orientation:
  X           0.7381614            0.6306580            0.2395585
  Y           0.6024892           -0.7760322            0.1864960
  Z           0.3035203            0.0066673           -0.9528016

Notes:  (1) The A matrix conforms to the "SAI" spin Hamiltonian convention.
        (2) Tensor is right-handed.

 -----------------------------------------------------------
 Nucleus  31C : A:ISTP=   13 I=  0.5 P=134.1903 MHz/au**3
               Q:ISTP=   13 I=  0.5 Q=  0.0000 barn
 -----------------------------------------------------------
 Raw HFC matrix (all values in MHz):
 -----------------------------------
                1.0825               0.1427              -0.4846
                0.1427               1.0309               0.1560
               -0.4846               0.1560               2.8654

 A(FC)          1.6596               1.6596               1.6596
 A(SD)         -0.8473              -0.4886               1.3359
             ----------           ----------           ----------
 A(Tot)         0.8123               1.1710               2.9955    A(iso)=    1.6596
 Orientation:
  X          -0.7362302            0.6323790            0.2409604
  Y           0.6391623            0.7667722           -0.0594291
  Z          -0.2223435            0.1102593           -0.9687137

Notes:  (1) The A matrix conforms to the "SAI" spin Hamiltonian convention.
        (2) Tensor is right-handed.

 -----------------------------------------------------------
 Nucleus  32C : A:ISTP=   13 I=  0.5 P=134.1903 MHz/au**3
               Q:ISTP=   13 I=  0.5 Q=  0.0000 barn
 -----------------------------------------------------------
 Raw HFC matrix (all values in MHz):
 -----------------------------------
               -0.2533               0.0135               0.6622
                0.0135               0.0690               0.2187
                0.6622               0.2187              -1.3727

 A(FC)         -0.5190              -0.5190              -0.5190
 A(SD)          0.4858               0.6963              -1.1821
             ----------           ----------           ----------
 A(Tot)        -0.0332               0.1773              -1.7011    A(iso)=   -0.5190
 Orientation:
  X           0.7254896            0.5508210           -0.4126270
  Y          -0.6395683            0.7610244           -0.1086016
  Z           0.2541992            0.3426924            0.9044029

Notes:  (1) The A matrix conforms to the "SAI" spin Hamiltonian convention.
        (2) Tensor is right-handed.
```

```
 -----------------------------------------------------------
 Nucleus  33C : A:ISTP=   13 I=  0.5 P=134.1903 MHz/au**3
                Q:ISTP=   13 I=  0.5 Q=  0.0000 barn
 -----------------------------------------------------------
 Raw HFC matrix (all values in MHz):
 -----------------------------------
                0.5904               0.1596              -0.7390
                0.1596               0.0981              -0.0331
               -0.7390              -0.0331               2.3647

 A(FC)          1.0177               1.0177               1.0177
 A(SD)         -0.9881              -0.6292               1.6173
            ----------           ----------           ----------
 A(Tot)         0.0296               0.3885               2.6351    A(iso)=    1.0177
 Orientation:
  X          0.4122109            0.8444415           -0.3420537
  Y         -0.9034597            0.4273407           -0.0337714
  Z          0.1176555            0.3229527            0.9390733

Notes:  (1) The A matrix conforms to the "SAI" spin Hamiltonian convention.
        (2) Tensor is right-handed.

 -----------------------------------------------------------
 Nucleus  34C : A:ISTP=   13 I=  0.5 P=134.1903 MHz/au**3
                Q:ISTP=   13 I=  0.5 Q=  0.0000 barn
 -----------------------------------------------------------
 Raw HFC matrix (all values in MHz):
 -----------------------------------
               -0.2499               0.4547               0.9919
                0.4547               0.2756               0.1049
                0.9919               0.1049              -1.9147

 A(FC)         -0.6297              -0.6297              -0.6297
 A(SD)          0.4183               1.3329              -1.7512
            ----------           ----------           ----------
 A(Tot)        -0.2114               0.7032              -2.3809    A(iso)=   -0.6297
 Orientation:
  X         -0.6508310            0.6268821            0.4282964
  Y          0.6802703            0.7319929           -0.0376662
  Z         -0.3371221            0.2668430           -0.9028530

Notes:  (1) The A matrix conforms to the "SAI" spin Hamiltonian convention.
        (2) Tensor is right-handed.

 -----------------------------------------------------------
 Nucleus  43C : A:ISTP=   13 I=  0.5 P=134.1903 MHz/au**3
                Q:ISTP=   13 I=  0.5 Q=  0.0000 barn
 -----------------------------------------------------------
 Raw HFC matrix (all values in MHz):
 -----------------------------------
               -2.0515               0.0070              -0.0938
                0.0070              -1.6408               0.0178
               -0.0938               0.0178              -1.9092

 A(FC)         -1.8672              -1.8672              -1.8672
 A(SD)          0.2275               0.0038              -0.2313
            ----------           ----------           ----------
 A(Tot)        -1.6396              -1.8634              -2.0985    A(iso)=   -1.8672
 Orientation:
  X          0.0021216           -0.4470990            0.8944820
  Y          0.9978681           -0.0573993           -0.0310573
  Z          0.0652283            0.8926409            0.4460240

Notes:  (1) The A matrix conforms to the "SAI" spin Hamiltonian convention.
        (2) Tensor is right-handed.
```

```
 -----------------------------------------------------------
 Nucleus  44C : A:ISTP=   13 I=  0.5 P=134.1903 MHz/au**3
                Q:ISTP=   13 I=  0.5 Q=  0.0000 barn
 -----------------------------------------------------------
 Raw HFC matrix (all values in MHz):
 -----------------------------------
                 1.8531               0.1012              -0.0668
                 0.1012               2.1666              -0.2027
                -0.0668              -0.2027               1.9624

 A(FC)           1.9940               1.9940               1.9940
 A(SD)          -0.1730              -0.1557               0.3288
            ----------           ----------           ----------
 A(Tot)          1.8210               1.8383               2.3228    A(iso)=    1.9940
 Orientation:
  X          0.9456063           -0.2082455           -0.2499249
  Y         -0.0944079            0.5595293           -0.8234161
  Z          0.3113130            0.8022224            0.5094344

Notes:  (1) The A matrix conforms to the "SAI" spin Hamiltonian convention.
        (2) Tensor is right-handed.

 -----------------------------------------------------------
 Nucleus  45C : A:ISTP=   13 I=  0.5 P=134.1903 MHz/au**3
                Q:ISTP=   13 I=  0.5 Q=  0.0000 barn
 -----------------------------------------------------------
 Raw HFC matrix (all values in MHz):
 -----------------------------------
                 0.1054               0.0161              -0.0091
                 0.0161               0.2812              -0.0193
                -0.0091              -0.0193               0.1195

 A(FC)           0.1687               0.1687               0.1687
 A(SD)          -0.0680              -0.0484               0.1164
            ----------           ----------           ----------
 A(Tot)          0.1007               0.1202               0.2851    A(iso)=    0.1687
 Orientation:
  X          0.9145052           -0.3933685           -0.0945591
  Y         -0.0384330            0.1481998           -0.9882104
  Z          0.4027445            0.9073577            0.1204111

Notes:  (1) The A matrix conforms to the "SAI" spin Hamiltonian convention.
        (2) Tensor is right-handed.

 -----------------------------------------------------------
 Nucleus  46C : A:ISTP=   13 I=  0.5 P=134.1903 MHz/au**3
                Q:ISTP=   13 I=  0.5 Q=  0.0000 barn
 -----------------------------------------------------------
 Raw HFC matrix (all values in MHz):
 -----------------------------------
                -0.0229              -0.0004               0.0103
                -0.0004               0.0541               0.0060
                 0.0103               0.0060              -0.0391

 A(FC)          -0.0026              -0.0026              -0.0026
 A(SD)          -0.0153              -0.0418               0.0571
            ----------           ----------           ----------
 A(Tot)         -0.0179              -0.0444               0.0545    A(iso)=   -0.0026
 Orientation:
  X         -0.9012111           -0.4333706            0.0029249
  Y          0.0302828           -0.0562388            0.9979580
  Z         -0.4323212            0.8994593            0.0638067

Notes:  (1) The A matrix conforms to the "SAI" spin Hamiltonian convention.
        (2) Tensor is right-handed.
```

```
 -----------------------------------------------------------
 Nucleus  47C : A:ISTP=   13 I=  0.5 P=134.1903 MHz/au**3
                Q:ISTP=   13 I=  0.5 Q=  0.0000 barn
 -----------------------------------------------------------
 Raw HFC matrix (all values in MHz):
 -----------------------------------
                 0.1030              -0.0141              -0.0109
                -0.0141               0.2710               0.0388
                -0.0109               0.0388               0.1245

 A(FC)           0.1662               0.1662               0.1662
 A(SD)          -0.0678              -0.0482               0.1160
            ----------           ----------           ----------
 A(Tot)          0.0984               0.1180               0.2821    A(iso)=    0.1662
 Orientation:
  X          0.9144635            0.3943895           -0.0906280
  Y         -0.0164251            0.2599461            0.9654834
  Z          0.4043350           -0.8814108            0.2441891

Notes:  (1) The A matrix conforms to the "SAI" spin Hamiltonian convention.
        (2) Tensor is right-handed.

 -----------------------------------------------------------
 Nucleus  48C : A:ISTP=   13 I=  0.5 P=134.1903 MHz/au**3
                Q:ISTP=   13 I=  0.5 Q=  0.0000 barn
 -----------------------------------------------------------
 Raw HFC matrix (all values in MHz):
 -----------------------------------
                 1.8479              -0.0901              -0.0771
                -0.0901               2.1098               0.2226
                -0.0771               0.2226               2.0122

 A(FC)           1.9900               1.9900               1.9900
 A(SD)          -0.1727              -0.1561               0.3288
            ----------           ----------           ----------
 A(Tot)          1.8173               1.8339               2.3187    A(iso)=    1.9900
 Orientation:
  X          0.9474506            0.2068586           -0.2440222
  Y          0.0514528            0.6543334            0.7544537
  Z          0.3157371           -0.7273633            0.6093051

Notes:  (1) The A matrix conforms to the "SAI" spin Hamiltonian convention.
        (2) Tensor is right-handed.

 -----------------------------------------------------------
 Nucleus  49C : A:ISTP=   13 I=  0.5 P=134.1903 MHz/au**3
                Q:ISTP=   13 I=  0.5 Q=  0.0000 barn
 -----------------------------------------------------------
 Raw HFC matrix (all values in MHz):
 -----------------------------------
                 0.0727              -0.0379              -0.0464
                -0.0379               0.3014               0.0985
                -0.0464               0.0985               0.1620

 A(FC)           0.1787               0.1787               0.1787
 A(SD)          -0.1258              -0.0584               0.1842
            ----------           ----------           ----------
 A(Tot)          0.0529               0.1203               0.3629    A(iso)=    0.1787
 Orientation:
  X          0.9125082            0.3636249           -0.1873651
  Y         -0.0229431            0.5028148            0.8640896
  Z          0.4084144           -0.7841902            0.4671653

Notes:  (1) The A matrix conforms to the "SAI" spin Hamiltonian convention.
        (2) Tensor is right-handed.
```

```
 -----------------------------------------------------------
 Nucleus  50C : A:ISTP=   13 I=  0.5 P=134.1903 MHz/au**3
                Q:ISTP=   13 I=  0.5 Q=  0.0000 barn
 -----------------------------------------------------------
 Raw HFC matrix (all values in MHz):
 -----------------------------------
              -0.0121            -0.0235            -0.0175
              -0.0235             0.0539             0.0536
              -0.0175             0.0536             0.0232

 A(FC)         0.0217             0.0217             0.0217
 A(SD)        -0.0388            -0.0413             0.0801
            ----------         ----------         ----------
 A(Tot)       -0.0171            -0.0196             0.1018    A(iso)=    0.0217
 Orientation:
  X         -0.0760102          0.9654806          0.2491378
  Y          0.5841403          0.2456117         -0.7735988
  Z         -0.8080858          0.0867300         -0.5826451

Notes:  (1) The A matrix conforms to the "SAI" spin Hamiltonian convention.
        (2) Tensor is right-handed.

 -----------------------------------------------------------
 Nucleus  51C : A:ISTP=   13 I=  0.5 P=134.1903 MHz/au**3
                Q:ISTP=   13 I=  0.5 Q=  0.0000 barn
 -----------------------------------------------------------
 Raw HFC matrix (all values in MHz):
 -----------------------------------
               0.0209            -0.0141            -0.0147
              -0.0141             0.0959             0.0342
              -0.0147             0.0342             0.0489

 A(FC)         0.0552             0.0552             0.0552
 A(SD)        -0.0406            -0.0219             0.0625
            ----------         ----------         ----------
 A(Tot)        0.0146             0.0333             0.1178    A(iso)=    0.0552
 Orientation:
  X          0.9145975          0.3532908         -0.1967156
  Y         -0.0116602          0.5093194          0.8604986
  Z          0.4041973         -0.7847161          0.4699417

Notes:  (1) The A matrix conforms to the "SAI" spin Hamiltonian convention.
        (2) Tensor is right-handed.

 -----------------------------------------------------------
 Nucleus  52C : A:ISTP=   13 I=  0.5 P=134.1903 MHz/au**3
                Q:ISTP=   13 I=  0.5 Q=  0.0000 barn
 -----------------------------------------------------------
 Raw HFC matrix (all values in MHz):
 -----------------------------------
               0.0655            -0.0137            -0.0062
              -0.0137             0.1321             0.0348
              -0.0062             0.0348             0.0821

 A(FC)         0.0932             0.0932             0.0932
 A(SD)        -0.0306            -0.0287             0.0593
            ----------         ----------         ----------
 A(Tot)        0.0626             0.0645             0.1525    A(iso)=    0.0932
 Orientation:
  X          0.9038268         -0.3925424         -0.1703163
  Y          0.3203823          0.3569679          0.8774561
  Z         -0.2836413         -0.8476346          0.4484007

Notes:  (1) The A matrix conforms to the "SAI" spin Hamiltonian convention.
        (2) Tensor is right-handed.
```

```
 -----------------------------------------------------------
 Nucleus  53C : A:ISTP=   13 I=  0.5 P=134.1903 MHz/au**3
                Q:ISTP=   13 I=  0.5 Q=  0.0000 barn
 -----------------------------------------------------------
 Raw HFC matrix (all values in MHz):
 -----------------------------------
                0.0659              0.0147              -0.0046
                0.0147              0.1404              -0.0274
               -0.0046             -0.0274               0.0747

 A(FC)          0.0937              0.0937               0.0937
 A(SD)         -0.0307             -0.0286               0.0594
            ----------          ----------          ----------
 A(Tot)         0.0629              0.0651               0.1530    A(iso)=    0.0937
 Orientation:
  X          0.9185135           0.3549968          -0.1740982
  Y         -0.2751724           0.2577488          -0.9261996
  Z         -0.2839243           0.8986339           0.3344310

Notes:  (1) The A matrix conforms to the "SAI" spin Hamiltonian convention.
        (2) Tensor is right-handed.

 -----------------------------------------------------------
 Nucleus  54C : A:ISTP=   13 I=  0.5 P=134.1903 MHz/au**3
                Q:ISTP=   13 I=  0.5 Q=  0.0000 barn
 -----------------------------------------------------------
 Raw HFC matrix (all values in MHz):
 -----------------------------------
                0.0210              0.0163              -0.0130
                0.0163              0.1037              -0.0272
               -0.0130             -0.0272               0.0410

 A(FC)          0.0552              0.0552               0.0552
 A(SD)         -0.0408             -0.0220               0.0627
            ----------          ----------          ----------
 A(Tot)         0.0145              0.0333               0.1180    A(iso)=    0.0552
 Orientation:
  X          0.9135482          -0.3536313          -0.2009343
  Y         -0.0430470           0.4071843          -0.9123310
  Z          0.4044461           0.8421080           0.3567597

Notes:  (1) The A matrix conforms to the "SAI" spin Hamiltonian convention.
        (2) Tensor is right-handed.

 -----------------------------------------------------------
 Nucleus  55C : A:ISTP=   13 I=  0.5 P=134.1903 MHz/au**3
                Q:ISTP=   13 I=  0.5 Q=  0.0000 barn
 -----------------------------------------------------------
 Raw HFC matrix (all values in MHz):
 -----------------------------------
               -0.0114              0.0261              -0.0147
                0.0261              0.0677              -0.0480
               -0.0147             -0.0480               0.0107

 A(FC)          0.0223              0.0223               0.0223
 A(SD)         -0.0389             -0.0416               0.0805
            ----------          ----------          ----------
 A(Tot)        -0.0166             -0.0193               0.1028    A(iso)=    0.0223
 Orientation:
  X          0.0494454           0.9660927           0.2534167
  Y          0.4825074          -0.2452601           0.8408532
  Z          0.8744952           0.0806991          -0.4782738

Notes:  (1) The A matrix conforms to the "SAI" spin Hamiltonian convention.
        (2) Tensor is right-handed.
```

```
 ------------------------------------------------------------
 Nucleus  56C : A:ISTP=   13 I=  0.5 P=134.1903 MHz/au**3
               Q:ISTP=   13 I=  0.5 Q=  0.0000 barn
 ------------------------------------------------------------
 Raw HFC matrix (all values in MHz):
 -----------------------------------
                0.0736                0.0452               -0.0418
                0.0452                0.3244               -0.0778
               -0.0418               -0.0778                0.1398

 A(FC)          0.1793                0.1793                0.1793
 A(SD)         -0.1261               -0.0587                0.1848
            ----------            ----------            ----------
 A(Tot)         0.0531                0.1206                0.3641    A(iso)=    0.1793
 Orientation:
  X          0.9119291            -0.3620089            -0.1932221
  Y         -0.0345447             0.4014762            -0.9152177
  Z          0.4088911             0.8412885             0.3536124

Notes:  (1) The A matrix conforms to the "SAI" spin Hamiltonian convention.
        (2) Tensor is right-handed.
```

6.2.2 FAAF

```
=======================================================================
                                       INPUT FILE
=======================================================================
|   1> !UKS BP86 EPR-II AUTOAUX
|   2> %PAL NPROCS 48 END
|   3> * xyz 0 3
|   4>   C   13.05217068545441     -5.38275929698126      5.07633930861002
|   5>   C   13.07510708187007     -4.23011590915238      4.16695633950574
|   6>   C   14.41940130276953     -5.73436699505536      5.36563504159523
|   7>   C   15.30099361482230     -4.83630408433735      4.64002902414982
|   8>   C   14.45588808521232     -3.91508386728665      3.90043374329446
|   9>   C   12.05344624790166     -3.46498274169684      3.59298214345855
|  10>   C   12.39509011741517     -2.38282016245205      2.75343722703529
|  11>   C   13.74611518539203     -2.06579437444035      2.49609877262615
|  12>   C   14.78299630616278     -2.82252151497713      3.06664658961905
|  13>   H   10.99526312837329     -3.69562985365025      3.79332779486639
|  14>   H   11.59739731616106     -1.77608089699410      2.29752755755106
|  15>   H   13.98959254089307     -1.21410397833139      1.84231686864401
|  16>   H   15.83576358823877     -2.57033014302004      2.86703136311659
|  17>   C   12.00106091985174     -6.12086088026723      5.63233206535387
|  18>   C   12.29976740559637     -7.21226313349386      6.47629009353148
|  19>   C   13.63740121842866     -7.56512952471901      6.75585399334559
|  20>   C   14.70337607872051     -6.83576923425122      6.20360690566596
|  21>   H   10.95285306448974     -5.86232339147087      5.41439054238660
|  22>   H   11.47879419453856     -7.79802867019155      6.91809739943334
|  23>   H   13.84725136022815     -8.42345890627017      7.41262165499401
|  24>   H   15.74556108254195     -7.11607704167162      6.42047993130067
|  25>   H   29.83013527243727     -6.02319905633685      3.61150559415807
|  26>   C   28.77093900764522     -6.20327895304697      3.36910489332347
|  27>   H   29.22068664944762     -7.50918800291117      1.68656296923601
|  28>   C   28.42525418368792     -7.03717243908930      2.28387996692667
|  29>   H   29.87972662352383     -4.40059101609161      5.77638980075094
|  30>   C   28.83317299720050     -4.16183437615296      6.02296849887507
|  31>   C   28.53886826808707     -3.31045823964042      7.10978885881674
|  32>   C   27.75205826095216     -5.60856233844192      4.12204679324502
|  33>   C   27.07311915591463     -7.26835582260040      1.95178824026190
|  34>   H   29.36172287435693     -2.88379640217913      7.70407680435740
|  35>   C   27.77969840297521     -4.69821927408131      5.27382446613240
|  36>   H   26.82650585572094     -7.91816375559329      1.09795674643012
|  37>   C   26.37002506837185     -5.84817618992964      3.79151510960388
|  38>   C   26.03910669236658     -6.67666404853664      2.69608854098073
```

```
| 39>   C   27.20308408139013     -3.00425497444085      7.44722853596782
| 40>   C   26.41450358376697     -4.38142093526273      5.60939079202639
| 41>   C   26.13472349223454     -3.53647127889542      6.70655155459185
| 42>   C   25.52911596383322     -5.09024528766291      4.70177446055238
| 43>   H   26.99649894936387     -2.34222250324501      8.30229659286268
| 44>   H   24.98559310470840     -6.85593325943153      2.43205276288563
| 45>   H   25.09396661252537     -3.29868257587299      6.97499297623377
| 46>   C   17.50312186171871     -5.15943699686102      3.45711557461859
| 47>   C   18.95833059254020     -5.17628642071841      3.47172427296249
| 48>   C   16.77645337941273     -4.85893777558693      4.65186058546002
| 49>   C   17.49207914704205     -4.58423511671356      5.85927146028520
| 50>   C   18.94677261744214     -4.62806945939302      5.87309445853491
| 51>   C   19.66505565716314     -4.91899600190592      4.67980768919688
| 52>   C   16.81503277435332     -4.21699025564013      7.07191813287129
| 53>   C   17.51119932279213     -3.95373112707946      8.23757935383620
| 54>   C   18.93578898358012     -4.03254021457547      8.25896411712104
| 55>   C   19.62982672917112     -4.35380516801571      7.10767681153047
| 56>   C   19.65322376395508     -5.47841669496419      2.25026871816288
| 57>   C   16.83520240891924     -5.49710910427880      2.23086264328306
| 58>   C   17.54239332382229     -5.78759670212668      1.07838431509993
| 59>   C   18.96913965185684     -5.76870409852838      1.08495984694565
| 60>   H   15.73664784875455     -5.52802334632796      2.22197379629717
| 61>   H   17.00217854727273     -6.04152117449441      0.15322899825840
| 62>   H   19.52478762582204     -5.99764526634918      0.16244649233967
| 63>   H   20.75279377743778     -5.48020461613213      2.26161090056850
| 64>   H   15.71871632534322     -4.14032923852117      7.05917369748317
| 65>   H   16.96424261733987     -3.67539611147508      9.15168032622827
| 66>   H   20.72858069972392     -4.39853321080591      7.11801394444504
| 67>   H   19.48243468986843     -3.82519770391847      9.19190807101081
| 68>   C   23.41247271289314     -1.44527394341372      3.78251488074781
| 69>   C   21.98722169709793     -1.39524872790796      3.82892105482814
| 70>   H   23.98391044749801     -0.54238560585229      3.51705664557724
| 71>   H   21.46422411566183     -0.45146727510309      3.60994815197180
| 72>   C   21.26323126244191     -2.53257655031857      4.13325838532172
| 73>   C   24.07894402512006     -2.62469755640321      4.06085497172505
| 74>   C   23.36971452992201     -3.82344869731558      4.41271923073213
| 75>   C   21.91495049872967     -3.78054537476285      4.42286899314730
| 76>   H   20.16416761135868     -2.50030862110911      4.15270186915944
| 77>   H   25.17619053129847     -2.65907324357386      4.00879959880039
| 78>   C   24.05417424492682     -5.04560907172084      4.70151736242000
| 79>   C   21.16605871878014     -4.95981009808370      4.69391497439681
| 80>   C   23.29664148208720     -6.22436134659988      4.98877323680872
| 81>   C   21.84203484333756     -6.18068278858467      4.97184564134614
| 82>   C   23.93199204901157     -7.46310709727823      5.34312062041772
| 83>   C   21.11598777182527     -7.38745400258505      5.25921281316497
| 84>   H   25.02919770378151     -7.49390629664252      5.39920902445353
| 85>   C   23.19532265298880     -8.60058785280263      5.61926570985774
| 86>   C   21.76980076871060     -8.56550212521946      5.56742940663896
| 87>   H   20.01710993354700     -7.35434827736695      5.23535047292629
| 88>   H   23.71088562548952     -9.53581216339537      5.88716429920079
| 89>   H   21.19068880258739     -9.47623214939449      5.78510913151463
| 90> *
| 91> %EPRNMR
| 92>         NUCLEI    = ALL C {AISO, ADIP}
| 93>         NUCLEI   = ALL H {AISO, ADIP}
| 94> END
| 95>


-----------------------------------------
ELECTRIC AND MAGNETIC HYPERFINE STRUCTURE
-----------------------------------------

 -----------------------------------------------------------
 Nucleus   0C : A:ISTP=   13 I=  0.5 P=134.1903 MHz/au**3
                Q:ISTP=   13 I=  0.5 Q=  0.0000 barn
```

```
 ----------------------------------------------------------
 Raw HFC matrix (all values in MHz):
 ----------------------------------
               -1.9447                -0.0249               -0.3396
               -0.0249                 2.5041                5.6502
               -0.3396                 5.6502                5.1015

 A(FC)          1.8869                 1.8869                1.8869
 A(SD)         -3.6669                -4.0533                7.7202
            ----------             ----------            ----------
 A(Tot)        -1.7800                -2.1663                9.6072    A(iso)=    1.8869
 Orientation:
  X          -0.7453423              0.6662374            -0.0243439
  Y          -0.5328236             -0.5733459             0.6223933
  Z           0.4007042              0.4768670             0.7823260

Notes:  (1) The A matrix conforms to the "SAI" spin Hamiltonian convention.
        (2) Tensor is right-handed.

 ----------------------------------------------------------
 Nucleus   1C : A:ISTP=   13 I=  0.5 P=134.1903 MHz/au**3
               Q:ISTP=   13 I=  0.5 Q=  0.0000 barn
 ----------------------------------------------------------
 Raw HFC matrix (all values in MHz):
 ----------------------------------
               -1.9518                 0.0432                0.3641
                0.0432                 2.5188                5.6537
                0.3641                 5.6537                5.1252

 A(FC)          1.8974                 1.8974                1.8974
 A(SD)         -3.6744                -4.0605                7.7350
            ----------             ----------            ----------
 A(Tot)        -1.7770                -2.1631                9.6324    A(iso)=    1.8974
 Orientation:
  X          -0.7246553             -0.6885858             0.0269128
  Y           0.5513461             -0.5559135             0.6220753
  Z          -0.4133910              0.4656284             0.7824948

Notes:  (1) The A matrix conforms to the "SAI" spin Hamiltonian convention.
        (2) Tensor is right-handed.

 ----------------------------------------------------------
 Nucleus   2C : A:ISTP=   13 I=  0.5 P=134.1903 MHz/au**3
               Q:ISTP=   13 I=  0.5 Q=  0.0000 barn
 ----------------------------------------------------------
 Raw HFC matrix (all values in MHz):
 ----------------------------------
               -9.9962                 0.2410               -0.4325
                0.2410               -11.3711               -2.8816
               -0.4325                -2.8816              -12.5225

 A(FC)        -11.2966               -11.2966              -11.2966
 A(SD)          2.4705                 1.1250               -3.5955
            ----------             ----------            ----------
 A(Tot)        -8.8261               -10.1716              -14.8922    A(iso)=  -11.2966
 Orientation:
  X           0.3676940              0.9291966            -0.0373476
  Y           0.7120629             -0.3071485            -0.6313685
  Z          -0.5981367              0.2055565            -0.7745831

Notes:  (1) The A matrix conforms to the "SAI" spin Hamiltonian convention.
        (2) Tensor is right-handed.

 ----------------------------------------------------------
 Nucleus   3C : A:ISTP=   13 I=  0.5 P=134.1903 MHz/au**3
               Q:ISTP=   13 I=  0.5 Q=  0.0000 barn
```

```
 ----------------------------------------------------------
 Raw HFC matrix (all values in MHz):
 ----------------------------------
               -2.4596               0.1055               0.1251
                0.1055              21.2589              28.7918
                0.1251              28.7918              32.7478

 A(FC)         17.1824              17.1824              17.1824
 A(SD)        -19.5383             -19.6425              39.1807
            ----------           ----------           ----------
 A(Tot)        -2.3559              -2.4601              56.3631    A(iso)=   17.1824
 Orientation:
  X          0.0213817            0.9997675           -0.0027811
  Y          0.7729806           -0.0182956           -0.6341659
  Z         -0.6340693            0.0114098           -0.7731920

Notes:  (1) The A matrix conforms to the "SAI" spin Hamiltonian convention.
        (2) Tensor is right-handed.

 ----------------------------------------------------------
 Nucleus   4C : A:ISTP=   13 I=  0.5 P=134.1903 MHz/au**3
                Q:ISTP=   13 I=  0.5 Q=  0.0000 barn
 ----------------------------------------------------------
 Raw HFC matrix (all values in MHz):
 ----------------------------------
              -10.0253              -0.2316               0.3941
               -0.2316             -11.3629              -2.8993
                0.3941              -2.8993             -12.5146

 A(FC)        -11.3009             -11.3009             -11.3009
 A(SD)          2.4725               1.1264              -3.5989
            ----------           ----------           ----------
 A(Tot)        -8.8284             -10.1745             -14.8998    A(iso)=  -11.3009
 Orientation:
  X         -0.3385122            0.9403994            0.0325331
  Y          0.7216721            0.2816537           -0.6323453
  Z         -0.6038202           -0.1905783           -0.7740032

Notes:  (1) The A matrix conforms to the "SAI" spin Hamiltonian convention.
        (2) Tensor is right-handed.

 ----------------------------------------------------------
 Nucleus   5C : A:ISTP=   13 I=  0.5 P=134.1903 MHz/au**3
                Q:ISTP=   13 I=  0.5 Q=  0.0000 barn
 ----------------------------------------------------------
 Raw HFC matrix (all values in MHz):
 ----------------------------------
               -3.3640              -0.1401               0.1006
               -0.1401              -3.8578              -1.1374
                0.1006              -1.1374              -4.4333

 A(FC)         -3.8850              -3.8850              -3.8850
 A(SD)          0.9777               0.4560              -1.4337
            ----------           ----------           ----------
 A(Tot)        -2.9073              -3.4290              -5.3188    A(iso)=   -3.8850
 Orientation:
  X         -0.3530827            0.9355858           -0.0034451
  Y          0.7388532            0.2765747           -0.6144936
  Z         -0.5739587           -0.2195125           -0.7889143

Notes:  (1) The A matrix conforms to the "SAI" spin Hamiltonian convention.
        (2) Tensor is right-handed.

 ----------------------------------------------------------
 Nucleus   6C : A:ISTP=   13 I=  0.5 P=134.1903 MHz/au**3
                Q:ISTP=   13 I=  0.5 Q=  0.0000 barn
```

```
 ------------------------------------------------------------
 Raw HFC matrix (all values in MHz):
 -----------------------------------
               -0.9838               -0.0634                0.2668
               -0.0634                4.6972                7.3403
                0.2668                7.3403                8.6126

 A(FC)          4.1087                4.1087                4.1087
 A(SD)         -4.8586               -5.2865               10.1451
            ----------            ----------            ----------
 A(Tot)        -0.7500               -1.1778               14.2538    A(iso)=    4.1087
 Orientation:
  X          -0.6699050             0.7423600            -0.0113552
  Y           0.5934348             0.5261990            -0.6090564
  Z          -0.4461641            -0.4147485            -0.7930456

Notes:  (1) The A matrix conforms to the "SAI" spin Hamiltonian convention.
        (2) Tensor is right-handed.

 ------------------------------------------------------------
 Nucleus   7C : A:ISTP=   13 I=  0.5 P=134.1903 MHz/au**3
               Q:ISTP=   13 I=  0.5 Q=  0.0000 barn
 ------------------------------------------------------------
 Raw HFC matrix (all values in MHz):
 -----------------------------------
               -3.2521               -0.1471                0.0588
               -0.1471               -4.8073               -1.6846
                0.0588               -1.6846               -5.7553

 A(FC)         -4.6049               -4.6049               -4.6049
 A(SD)          1.4205                1.0064               -2.4269
            ----------            ----------            ----------
 A(Tot)        -3.1844               -3.5985               -7.0318    A(iso)=   -4.6049
 Orientation:
  X           0.9151724             0.4029089             0.0111280
  Y          -0.3272272             0.7265818             0.6041533
  Z           0.2353334            -0.5565459             0.7967904

Notes:  (1) The A matrix conforms to the "SAI" spin Hamiltonian convention.
        (2) Tensor is right-handed.

 ------------------------------------------------------------
 Nucleus   8C : A:ISTP=   13 I=  0.5 P=134.1903 MHz/au**3
               Q:ISTP=   13 I=  0.5 Q=  0.0000 barn
 ------------------------------------------------------------
 Raw HFC matrix (all values in MHz):
 -----------------------------------
               -0.7620               -0.0495                0.0081
               -0.0495                4.4872                6.8681
                0.0081                6.8681                8.6462

 A(FC)          4.1238                4.1238                4.1238
 A(SD)         -4.7210               -4.8980                9.6190
            ----------            ----------            ----------
 A(Tot)        -0.5972               -0.7742               13.7428    A(iso)=    4.1238
 Orientation:
  X          -0.2613940             0.9652309             0.0015850
  Y           0.7748818             0.2108242            -0.5959121
  Z          -0.5755270            -0.1545397            -0.8030481

Notes:  (1) The A matrix conforms to the "SAI" spin Hamiltonian convention.
        (2) Tensor is right-handed.

 ------------------------------------------------------------
 Nucleus  13C : A:ISTP=   13 I=  0.5 P=134.1903 MHz/au**3
               Q:ISTP=   13 I=  0.5 Q=  0.0000 barn
```

```
 -----------------------------------------------------------
 Raw HFC matrix (all values in MHz):
 -----------------------------------
               -3.3485             0.1449             -0.1132
                0.1449            -3.8556             -1.1259
               -0.1132            -1.1259             -4.4272

 A(FC)         -3.8771            -3.8771             -3.8771
 A(SD)          0.9733             0.4526             -1.4259
            ----------         ----------          ----------
 A(Tot)        -2.9038            -3.4245             -5.3030    A(iso)=   -3.8771
 Orientation:
  X          0.3820706          0.9241331         -0.0002141
  Y          0.7293959         -0.3017014         -0.6139689
  Z         -0.5674536          0.2344233         -0.7893301

Notes:  (1) The A matrix conforms to the "SAI" spin Hamiltonian convention.
        (2) Tensor is right-handed.

 -----------------------------------------------------------
 Nucleus  14C : A:ISTP=   13 I=  0.5 P=134.1903 MHz/au**3
                Q:ISTP=   13 I=  0.5 Q=  0.0000 barn
 -----------------------------------------------------------
 Raw HFC matrix (all values in MHz):
 -----------------------------------
               -0.9715             0.0898             -0.2335
                0.0898             4.6690              7.3332
               -0.2335             7.3332              8.6051

 A(FC)          4.1009             4.1009              4.1009
 A(SD)         -4.8515            -5.2785             10.1300
            ----------         ----------          ----------
 A(Tot)        -0.7506            -1.1776             14.2309    A(iso)=    4.1009
 Orientation:
  X          0.6928837          0.7209981          0.0085942
  Y          0.5757917         -0.5460864         -0.6084847
  Z         -0.4340231          0.4265577         -0.7935190

Notes:  (1) The A matrix conforms to the "SAI" spin Hamiltonian convention.
        (2) Tensor is right-handed.

 -----------------------------------------------------------
 Nucleus  15C : A:ISTP=   13 I=  0.5 P=134.1903 MHz/au**3
                Q:ISTP=   13 I=  0.5 Q=  0.0000 barn
 -----------------------------------------------------------
 Raw HFC matrix (all values in MHz):
 -----------------------------------
               -3.2390             0.1337             -0.0612
                0.1337            -4.8050             -1.6758
               -0.0612            -1.6758             -5.7513

 A(FC)         -4.5985            -4.5985             -4.5985
 A(SD)          1.4176             1.0037             -2.4213
            ----------         ----------          ----------
 A(Tot)        -3.1809            -3.5947             -7.0198    A(iso)=   -4.5985
 Orientation:
  X          0.9274018         -0.3739717         -0.0084319
  Y          0.3028626          0.7374492          0.6036910
  Z         -0.2195453         -0.5624178          0.7971738

Notes:  (1) The A matrix conforms to the "SAI" spin Hamiltonian convention.
        (2) Tensor is right-handed.

 -----------------------------------------------------------
 Nucleus  16C : A:ISTP=   13 I=  0.5 P=134.1903 MHz/au**3
                Q:ISTP=   13 I=  0.5 Q=  0.0000 barn
```

```
 ----------------------------------------------------------
 Raw HFC matrix (all values in MHz):
 ----------------------------------
              -0.7600               0.0758               0.0196
               0.0758               4.4637               6.8582
               0.0196               6.8582               8.6497

 A(FC)         4.1178               4.1178               4.1178
 A(SD)        -4.7166              -4.8931               9.6096
            ----------           ----------           ----------
 A(Tot)       -0.5988              -0.7753              13.7274    A(iso)=    4.1178
 Orientation:
  X         0.2918573            0.9564526           -0.0042025
  Y         0.7679772           -0.2369593           -0.5950305
  Z        -0.5701143            0.1704366           -0.8036921

Notes:  (1) The A matrix conforms to the "SAI" spin Hamiltonian convention.
        (2) Tensor is right-handed.

 ----------------------------------------------------------
 Nucleus  22C : A:ISTP=   13 I=  0.5 P=134.1903 MHz/au**3
                Q:ISTP=   13 I=  0.5 Q=  0.0000 barn
 ----------------------------------------------------------
 Raw HFC matrix (all values in MHz):
 ----------------------------------
              -3.3618              -0.1356              -0.1108
              -0.1356              -4.4175               1.1356
              -0.1108               1.1356              -3.8574

 A(FC)        -3.8789              -3.8789              -3.8789
 A(SD)         0.9749               0.4540              -1.4289
            ----------           ----------           ----------
 A(Tot)       -2.9040              -3.4249              -5.3078    A(iso)=   -3.8789
 Orientation:
  X        -0.3500476            0.9365211            0.0198708
  Y         0.5820400            0.2008327            0.7879694
  Z         0.7339593            0.2873923           -0.6153937

Notes:  (1) The A matrix conforms to the "SAI" spin Hamiltonian convention.
        (2) Tensor is right-handed.

 ----------------------------------------------------------
 Nucleus  24C : A:ISTP=   13 I=  0.5 P=134.1903 MHz/au**3
                Q:ISTP=   13 I=  0.5 Q=  0.0000 barn
 ----------------------------------------------------------
 Raw HFC matrix (all values in MHz):
 ----------------------------------
              -0.9848               0.0037              -0.2707
               0.0037               8.5948              -7.3327
              -0.2707              -7.3327               4.6982

 A(FC)         4.1027               4.1027               4.1027
 A(SD)        -4.8528              -5.2799              10.1327
            ----------           ----------           ----------
 A(Tot)       -0.7501              -1.1772              14.2354    A(iso)=    4.1027
 Orientation:
  X         0.6678560           -0.7442087            0.0110339
  Y        -0.4596261           -0.4007194            0.7925703
  Z        -0.5854162           -0.5343943           -0.6096807

Notes:  (1) The A matrix conforms to the "SAI" spin Hamiltonian convention.
        (2) Tensor is right-handed.

 ----------------------------------------------------------
 Nucleus  26C : A:ISTP=   13 I=  0.5 P=134.1903 MHz/au**3
                Q:ISTP=   13 I=  0.5 Q=  0.0000 barn
```

```
 ----------------------------------------------------------
 Raw HFC matrix (all values in MHz):
 ----------------------------------
               -3.3504              0.0782              0.1738
                0.0782             -4.4344              1.1279
                0.1738              1.1279             -3.8590

 A(FC)         -3.8813             -3.8813             -3.8813
 A(SD)          0.9758              0.4547             -1.4305
            ----------          ----------          ----------
 A(Tot)        -2.9054             -3.4266             -5.3117    A(iso)=   -3.8813
 Orientation:
  X          0.3849393           0.9226538           0.0230568
  Y          0.5605483          -0.2535662           0.7883462
  Z          0.7332171          -0.2905410          -0.6147996

Notes:  (1) The A matrix conforms to the "SAI" spin Hamiltonian convention.
        (2) Tensor is right-handed.

 ----------------------------------------------------------
 Nucleus  27C : A:ISTP=   13 I=  0.5 P=134.1903 MHz/au**3
               Q:ISTP=   13 I=  0.5 Q=  0.0000 barn
 ----------------------------------------------------------
 Raw HFC matrix (all values in MHz):
 ----------------------------------
               -0.9567              0.5025             -0.1173
                0.5025              8.5729             -7.3402
               -0.1173             -7.3402              4.6971

 A(FC)          4.1045              4.1045              4.1045
 A(SD)         -4.8546             -5.2821             10.1368
            ----------          ----------          ----------
 A(Tot)        -0.7502             -1.1777             14.2412    A(iso)=    4.1045
 Orientation:
  X         -0.6946744          -0.7186605           0.0308948
  Y         -0.4214857           0.4414709           0.7921195
  Z         -0.5829042           0.5372434          -0.6095837

Notes:  (1) The A matrix conforms to the "SAI" spin Hamiltonian convention.
        (2) Tensor is right-handed.

 ----------------------------------------------------------
 Nucleus  28C : A:ISTP=   13 I=  0.5 P=134.1903 MHz/au**3
               Q:ISTP=   13 I=  0.5 Q=  0.0000 barn
 ----------------------------------------------------------
 Raw HFC matrix (all values in MHz):
 ----------------------------------
               -1.9635             -0.1731             -0.1086
               -0.1731              5.1082             -5.6513
               -0.1086             -5.6513              2.5247

 A(FC)          1.8898              1.8898              1.8898
 A(SD)         -3.6690             -4.0551              7.7241
            ----------          ----------          ----------
 A(Tot)        -1.7792             -2.1653              9.6139    A(iso)=    1.8898
 Orientation:
  X          0.7222884           0.6915673           0.0058438
  Y         -0.4277534           0.4533630          -0.7819777
  Z         -0.5434395           0.5623137           0.6232790

Notes:  (1) The A matrix conforms to the "SAI" spin Hamiltonian convention.
        (2) Tensor is right-handed.

 ----------------------------------------------------------
 Nucleus  29C : A:ISTP=   13 I=  0.5 P=134.1903 MHz/au**3
               Q:ISTP=   13 I=  0.5 Q=  0.0000 barn
```

```
 -----------------------------------------------------------
 Raw HFC matrix (all values in MHz):
 -----------------------------------
              -3.2495              -0.1269              -0.0961
              -0.1269              -5.7409               1.6842
              -0.0961               1.6842              -4.8083

 A(FC)        -4.5995              -4.5995              -4.5995
 A(SD)         1.4185               1.0046              -2.4231
            ----------           ----------           ----------
 A(Tot)       -3.1810              -3.5949              -7.0227    A(iso)=   -4.5995
 Orientation:
  X          0.9141625            0.4051883           -0.0113699
  Y         -0.2534565            0.5494940           -0.7961257
  Z         -0.3163331            0.7306700            0.6050245

Notes:  (1) The A matrix conforms to the "SAI" spin Hamiltonian convention.
        (2) Tensor is right-handed.

 -----------------------------------------------------------
 Nucleus  31C : A:ISTP=   13 I=  0.5 P=134.1903 MHz/au**3
                Q:ISTP=   13 I=  0.5 Q=  0.0000 barn
 -----------------------------------------------------------
 Raw HFC matrix (all values in MHz):
 -----------------------------------
              -1.9226               0.5301              -0.1778
               0.5301               5.0817              -5.6518
              -0.1778              -5.6518               2.5226

 A(FC)         1.8939               1.8939               1.8939
 A(SD)        -3.6703              -4.0566               7.7269
            ----------           ----------           ----------
 A(Tot)       -1.7764              -2.1628               9.6208    A(iso)=    1.8939
 Orientation:
  X          0.7473426           -0.6628820           -0.0454591
  Y          0.3872073            0.4900985           -0.7809443
  Z          0.5399534            0.5660308            0.6229442

Notes:  (1) The A matrix conforms to the "SAI" spin Hamiltonian convention.
        (2) Tensor is right-handed.

 -----------------------------------------------------------
 Nucleus  33C : A:ISTP=   13 I=  0.5 P=134.1903 MHz/au**3
                Q:ISTP=   13 I=  0.5 Q=  0.0000 barn
 -----------------------------------------------------------
 Raw HFC matrix (all values in MHz):
 -----------------------------------
             -10.0339              -0.4807              -0.1547
              -0.4807             -12.4789               2.8976
              -0.1547               2.8976             -11.3727

 A(FC)       -11.2952             -11.2952             -11.2952
 A(SD)         2.4706               1.1253              -3.5959
            ----------           ----------           ----------
 A(Tot)       -8.8246             -10.1699             -14.8910    A(iso)=  -11.2952
 Orientation:
  X         -0.3349371            0.9405619            0.0562181
  Y          0.6120740            0.1718240            0.7719080
  Z          0.7163676            0.2929503           -0.6332437

Notes:  (1) The A matrix conforms to the "SAI" spin Hamiltonian convention.
        (2) Tensor is right-handed.

 -----------------------------------------------------------
 Nucleus  34C : A:ISTP=   13 I=  0.5 P=134.1903 MHz/au**3
                Q:ISTP=   13 I=  0.5 Q=  0.0000 barn
```

```
 -----------------------------------------------------------
 Raw HFC matrix (all values in MHz):
 -----------------------------------
               -0.7543              0.2561             -0.2456
                0.2561              8.6125             -6.8630
               -0.2456             -6.8630              4.4970

 A(FC)          4.1184              4.1184              4.1184
 A(SD)         -4.7167             -4.8931              9.6098
            ----------          ----------          ----------
 A(Tot)        -0.5983             -0.7747             13.7282    A(iso)=    4.1184
 Orientation:
  X          -0.2588832           0.9656028           0.0243031
  Y           0.5817341           0.1357814           0.8019656
  Z           0.7710804           0.2217534          -0.5968756

Notes:  (1) The A matrix conforms to the "SAI" spin Hamiltonian convention.
        (2) Tensor is right-handed.

 -----------------------------------------------------------
 Nucleus  35C : A:ISTP=   13 I=  0.5 P=134.1903 MHz/au**3
                Q:ISTP=   13 I=  0.5 Q=  0.0000 barn
 -----------------------------------------------------------
 Raw HFC matrix (all values in MHz):
 -----------------------------------
               -3.2430             -0.0068              0.1844
               -0.0068             -5.7522              1.6761
                0.1844              1.6761             -4.8084

 A(FC)         -4.6012             -4.6012             -4.6012
 A(SD)          1.4190              1.0050             -2.4240
            ----------          ----------          ----------
 A(Tot)        -3.1822             -3.5962             -7.0252    A(iso)=   -4.6012
 Orientation:
  X           0.9279690          -0.3713751          -0.0308879
  Y           0.2018566           0.5705945          -0.7960376
  Z           0.3132530           0.7324633           0.6044585

Notes:  (1) The A matrix conforms to the "SAI" spin Hamiltonian convention.
        (2) Tensor is right-handed.

 -----------------------------------------------------------
 Nucleus  36C : A:ISTP=   13 I=  0.5 P=134.1903 MHz/au**3
                Q:ISTP=   13 I=  0.5 Q=  0.0000 barn
 -----------------------------------------------------------
 Raw HFC matrix (all values in MHz):
 -----------------------------------
               -9.9904              0.3452              0.3165
                0.3452            -12.5378              2.8818
                0.3165              2.8818            -11.3714

 A(FC)        -11.2999            -11.2999            -11.2999
 A(SD)          2.4707              1.1252             -3.5959
            ----------          ----------          ----------
 A(Tot)        -8.8292            -10.1747            -14.8958    A(iso)=  -11.2999
 Orientation:
  X           0.3709499           0.9285515          -0.0137237
  Y           0.5910901          -0.2246869           0.7746795
  Z           0.7162462          -0.2954792          -0.6322052

Notes:  (1) The A matrix conforms to the "SAI" spin Hamiltonian convention.
        (2) Tensor is right-handed.

 -----------------------------------------------------------
 Nucleus  37C : A:ISTP=   13 I=  0.5 P=134.1903 MHz/au**3
                Q:ISTP=   13 I=  0.5 Q=  0.0000 barn
```

```
 -----------------------------------------------------------
 Raw HFC matrix (all values in MHz):
 -----------------------------------
               -0.7539               0.2444              -0.1194
                0.2444               8.6372              -6.8623
               -0.1194              -6.8623               4.4784

 A(FC)          4.1206               4.1206               4.1206
 A(SD)         -4.7179              -4.8947               9.6126
             ----------           ----------           ----------
 A(Tot)        -0.5973              -0.7742              13.7332    A(iso)=    4.1206
 Orientation:
  X           0.2942538            0.9555491            0.0184545
  Y           0.5650223           -0.1895028            0.8030184
  Z           0.7708207           -0.2258640           -0.5956685

Notes:  (1) The A matrix conforms to the "SAI" spin Hamiltonian convention.
        (2) Tensor is right-handed.

 -----------------------------------------------------------
 Nucleus  38C : A:ISTP=   13 I=  0.5 P=134.1903 MHz/au**3
                Q:ISTP=   13 I=  0.5 Q=  0.0000 barn
 -----------------------------------------------------------
 Raw HFC matrix (all values in MHz):
 -----------------------------------
               -2.4293               1.0275              -0.8422
                1.0275              32.6437             -28.7874
               -0.8422             -28.7874              21.3235

 A(FC)         17.1793              17.1793              17.1793
 A(SD)        -19.5342             -19.6386              39.1729
             ----------           ----------           ----------
 A(Tot)        -2.3549              -2.4594              56.3521    A(iso)=   17.1793
 Orientation:
  X           0.0219549            0.9995036            0.0225956
  Y           0.6347095           -0.0313969            0.7721128
  Z           0.7724389           -0.0026100           -0.6350837

Notes:  (1) The A matrix conforms to the "SAI" spin Hamiltonian convention.
        (2) Tensor is right-handed.

 -----------------------------------------------------------
 Nucleus  42C : A:ISTP=   13 I=  0.5 P=134.1903 MHz/au**3
                Q:ISTP=   13 I=  0.5 Q=  0.0000 barn
 -----------------------------------------------------------
 Raw HFC matrix (all values in MHz):
 -----------------------------------
                9.4409               0.7995              -1.1787
                0.7995              10.1820              -1.6581
               -1.1787              -1.6581              10.1268

 A(FC)          9.9165               9.9165               9.9165
 A(SD)         -1.5682              -0.9776               2.5458
             ----------           ----------           ----------
 A(Tot)         8.3483               8.9389              12.4624    A(iso)=    9.9165
 Orientation:
  X           0.4589197            0.7820615            0.4216307
  Y           0.4774493           -0.6172868            0.6252992
  Z           0.7492896           -0.0856548           -0.6566798

Notes:  (1) The A matrix conforms to the "SAI" spin Hamiltonian convention.
        (2) Tensor is right-handed.

 -----------------------------------------------------------
 Nucleus  43C : A:ISTP=   13 I=  0.5 P=134.1903 MHz/au**3
                Q:ISTP=   13 I=  0.5 Q=  0.0000 barn
```

```
 ----------------------------------------------------------
 Raw HFC matrix (all values in MHz):
 ----------------------------------
               0.8613              0.0139              0.0619
               0.0139             -0.6045              0.2140
               0.0619              0.2140              0.4634

 A(FC)         0.2401              0.2401              0.2401
 A(SD)         0.2537             -0.8858              0.6322
            ----------          ----------          ----------
 A(Tot)        0.4938             -0.6457              0.8723    A(iso)=    0.2401
 Orientation:
  X         -0.1699013          -0.0012785          -0.9854603
  Y          0.1863658           0.9819124          -0.0334048
  Z          0.9676783          -0.1893316          -0.1665899

Notes:  (1) The A matrix conforms to the "SAI" spin Hamiltonian convention.
        (2) Tensor is right-handed.

 ----------------------------------------------------------
 Nucleus  44C : A:ISTP=   13 I=  0.5 P=134.1903 MHz/au**3
                Q:ISTP=   13 I=  0.5 Q=  0.0000 barn
 ----------------------------------------------------------
 Raw HFC matrix (all values in MHz):
 ----------------------------------
              -8.2105             -0.0618              0.0176
              -0.0618            -11.6850              0.8286
               0.0176              0.8286             -9.0080

 A(FC)        -9.6345             -9.6345             -9.6345
 A(SD)         1.4251              0.8622             -2.2873
            ----------          ----------          ----------
 A(Tot)       -8.2094             -8.7723            -11.9218    A(iso)=   -9.6345
 Orientation:
  X          0.9998499          -0.0000904          -0.0173253
  Y         -0.0166395           0.2736157          -0.9616952
  Z          0.0048274           0.9618391           0.2735731

Notes:  (1) The A matrix conforms to the "SAI" spin Hamiltonian convention.
        (2) Tensor is right-handed.

 ----------------------------------------------------------
 Nucleus  45C : A:ISTP=   13 I=  0.5 P=134.1903 MHz/au**3
                Q:ISTP=   13 I=  0.5 Q=  0.0000 barn
 ----------------------------------------------------------
 Raw HFC matrix (all values in MHz):
 ----------------------------------
               9.2466             -0.7248              1.0938
              -0.7248             10.1349             -1.7061
               1.0938             -1.7061             10.0766

 A(FC)         9.8194              9.8194              9.8194
 A(SD)        -1.5610             -0.9665              2.5275
            ----------          ----------          ----------
 A(Tot)        8.2584              8.8529             12.3469    A(iso)=    9.8194
 Orientation:
  X         -0.4540384           0.8037933          -0.3844029
  Y          0.4969880           0.5865611           0.6394912
  Z          0.7394945           0.0993099          -0.6657969

Notes:  (1) The A matrix conforms to the "SAI" spin Hamiltonian convention.
        (2) Tensor is right-handed.

 ----------------------------------------------------------
 Nucleus  46C : A:ISTP=   13 I=  0.5 P=134.1903 MHz/au**3
                Q:ISTP=   13 I=  0.5 Q=  0.0000 barn
```

```
 ----------------------------------------------------------
 Raw HFC matrix (all values in MHz):
 ----------------------------------
               0.8312              -0.0860              -0.0444
              -0.0860              -0.6213               0.2171
              -0.0444               0.2171               0.4351

 A(FC)         0.2150               0.2150               0.2150
 A(SD)         0.2531              -0.8830               0.6299
             ----------           ----------           ----------
 A(Tot)        0.4681              -0.6680               0.8449    A(iso)=    0.2150
 Orientation:
  X          0.1614889            0.0505586           -0.9855786
  Y          0.1805901            0.9803095            0.0798783
  Z          0.9702106           -0.1908852            0.1491787

Notes:  (1) The A matrix conforms to the "SAI" spin Hamiltonian convention.
        (2) Tensor is right-handed.

 ----------------------------------------------------------
 Nucleus  47C : A:ISTP=   13 I=  0.5 P=134.1903 MHz/au**3
                Q:ISTP=   13 I=  0.5 Q=  0.0000 barn
 ----------------------------------------------------------
 Raw HFC matrix (all values in MHz):
 ----------------------------------
              -0.9783               0.1509              -0.0242
               0.1509               4.4790              -0.9825
              -0.0242              -0.9825              -1.1520

 A(FC)         0.7829               0.7829               0.7829
 A(SD)        -1.7653              -2.1014               3.8667
             ----------           ----------           ----------
 A(Tot)       -0.9824              -1.3185               4.6496    A(iso)=    0.7829
 Orientation:
  X          0.9996231           -0.0040717           -0.0271492
  Y         -0.0260867            0.1671939           -0.9855789
  Z          0.0085522            0.9859156            0.1670246

Notes:  (1) The A matrix conforms to the "SAI" spin Hamiltonian convention.
        (2) Tensor is right-handed.

 ----------------------------------------------------------
 Nucleus  48C : A:ISTP=   13 I=  0.5 P=134.1903 MHz/au**3
                Q:ISTP=   13 I=  0.5 Q=  0.0000 barn
 ----------------------------------------------------------
 Raw HFC matrix (all values in MHz):
 ----------------------------------
               0.2611               0.1381               0.1864
               0.1381              -1.0418               0.2939
               0.1864               0.2939               0.1019

 A(FC)        -0.2263              -0.2263              -0.2263
 A(SD)         0.2281               0.6646              -0.8927
             ----------           ----------           ----------
 A(Tot)        0.0018               0.4383              -1.1190    A(iso)=   -0.2263
 Orientation:
  X          0.6245415            0.7781062            0.0670717
  Y         -0.1340573            0.1914121           -0.9723117
  Z         -0.7694001            0.5982576            0.2238557

Notes:  (1) The A matrix conforms to the "SAI" spin Hamiltonian convention.
        (2) Tensor is right-handed.

 ----------------------------------------------------------
 Nucleus  49C : A:ISTP=   13 I=  0.5 P=134.1903 MHz/au**3
                Q:ISTP=   13 I=  0.5 Q=  0.0000 barn
```

```
 -----------------------------------------------------------
 Raw HFC matrix (all values in MHz):
 -----------------------------------
               0.1135            0.0114            0.0545
               0.0114            1.2154           -0.3336
               0.0545           -0.3336            0.3057

 A(FC)         0.5449            0.5449            0.5449
 A(SD)        -0.4591           -0.3208            0.7798
            ----------        ----------        ----------
 A(Tot)        0.0858            0.2241            1.3247    A(iso)=    0.5449
 Orientation:
  X         0.8944239         0.4471918         0.0050427
  Y        -0.1349769         0.2806815        -0.9502627
  Z        -0.4263651         0.8492570         0.3114088

Notes:  (1) The A matrix conforms to the "SAI" spin Hamiltonian convention.
        (2) Tensor is right-handed.

 -----------------------------------------------------------
 Nucleus  50C : A:ISTP=   13 I=  0.5 P=134.1903 MHz/au**3
                Q:ISTP=   13 I=  0.5 Q=  0.0000 barn
 -----------------------------------------------------------
 Raw HFC matrix (all values in MHz):
 -----------------------------------
               0.0778           -0.0580            0.0375
              -0.0580           -0.7989            0.2018
               0.0375            0.2018           -0.0092

 A(FC)        -0.2434           -0.2434           -0.2434
 A(SD)         0.2729            0.3356           -0.6086
            ----------        ----------        ----------
 A(Tot)        0.0295            0.0922           -0.8520    A(iso)=   -0.2434
 Orientation:
  X        -0.3957385         0.9156939         0.0699701
  Y         0.2433812         0.0311066         0.9694318
  Z         0.8855262         0.4006708        -0.2351727

Notes:  (1) The A matrix conforms to the "SAI" spin Hamiltonian convention.
        (2) Tensor is right-handed.

 -----------------------------------------------------------
 Nucleus  51C : A:ISTP=   13 I=  0.5 P=134.1903 MHz/au**3
                Q:ISTP=   13 I=  0.5 Q=  0.0000 barn
 -----------------------------------------------------------
 Raw HFC matrix (all values in MHz):
 -----------------------------------
               0.7120           -0.0671            0.0713
              -0.0671            1.3731           -0.3527
               0.0713           -0.3527            0.7436

 A(FC)         0.9429            0.9429            0.9429
 A(SD)        -0.3683           -0.2298            0.5980
            ----------        ----------        ----------
 A(Tot)        0.5746            0.7131            1.5409    A(iso)=    0.9429
 Orientation:
  X        -0.2801747         0.9537864        -0.1085982
  Y         0.3681032         0.2112276         0.9054739
  Z         0.8865677         0.2137155        -0.4102724

Notes:  (1) The A matrix conforms to the "SAI" spin Hamiltonian convention.
        (2) Tensor is right-handed.

 -----------------------------------------------------------
 Nucleus  52C : A:ISTP=   13 I=  0.5 P=134.1903 MHz/au**3
                Q:ISTP=   13 I=  0.5 Q=  0.0000 barn
```

```
 -----------------------------------------------------------
 Raw HFC matrix (all values in MHz):
 -----------------------------------
                0.7159              0.0987              -0.0856
                0.0987              1.3484              -0.3411
               -0.0856             -0.3411               0.7285

 A(FC)          0.9310              0.9310               0.9310
 A(SD)         -0.3644             -0.2234               0.5878
             ----------          ----------          ----------
 A(Tot)         0.5665              0.7076               1.5188    A(iso)=    0.9310
 Orientation:
  X          0.2776374           0.9482623           0.1540000
  Y          0.3544401          -0.2501012           0.9010114
  Z          0.8929108          -0.1955707          -0.4055396

Notes:  (1) The A matrix conforms to the "SAI" spin Hamiltonian convention.
        (2) Tensor is right-handed.

 -----------------------------------------------------------
 Nucleus  53C : A:ISTP=   13 I=  0.5 P=134.1903 MHz/au**3
                Q:ISTP=   13 I=  0.5 Q=  0.0000 barn
 -----------------------------------------------------------
 Raw HFC matrix (all values in MHz):
 -----------------------------------
                0.2204             -0.1894              -0.1725
               -0.1894             -1.0558               0.2928
               -0.1725              0.2928               0.0735

 A(FC)         -0.2540             -0.2540              -0.2540
 A(SD)          0.2283              0.6603              -0.8887
             ----------          ----------          ----------
 A(Tot)        -0.0256              0.4064              -1.1426    A(iso)=   -0.2540
 Orientation:
  X          0.6238883           0.7741322           0.1071576
  Y          0.1053915          -0.2192032           0.9699704
  Z          0.7743746          -0.5938597          -0.2183453

Notes:  (1) The A matrix conforms to the "SAI" spin Hamiltonian convention.
        (2) Tensor is right-handed.

 -----------------------------------------------------------
 Nucleus  54C : A:ISTP=   13 I=  0.5 P=134.1903 MHz/au**3
                Q:ISTP=   13 I=  0.5 Q=  0.0000 barn
 -----------------------------------------------------------
 Raw HFC matrix (all values in MHz):
 -----------------------------------
                0.1168              0.0400              -0.0714
                0.0400              1.2008              -0.3248
               -0.0714             -0.3248               0.2994

 A(FC)          0.5390              0.5390               0.5390
 A(SD)         -0.4535             -0.3161               0.7697
             ----------          ----------          ----------
 A(Tot)         0.0855              0.2229               1.3087    A(iso)=    0.5390
 Orientation:
  X          0.8900766          -0.4530214          -0.0503503
  Y          0.0977545           0.2976130          -0.9496687
  Z          0.4452052           0.8403559           0.3091832

Notes:  (1) The A matrix conforms to the "SAI" spin Hamiltonian convention.
        (2) Tensor is right-handed.

 -----------------------------------------------------------
 Nucleus  55C : A:ISTP=   13 I=  0.5 P=134.1903 MHz/au**3
                Q:ISTP=   13 I=  0.5 Q=  0.0000 barn
```

```
 -----------------------------------------------------------
 Raw HFC matrix (all values in MHz):
 -----------------------------------
               0.0844              0.0152               -0.0271
               0.0152             -0.8021                0.2025
              -0.0271              0.2025               -0.0077

 A(FC)        -0.2418             -0.2418               -0.2418
 A(SD)         0.2731              0.3364               -0.6095
            ----------          ----------            ----------
 A(Tot)        0.0313              0.0946               -0.8513    A(iso)=   -0.2418
 Orientation:
  X          0.3915885           0.9198625            -0.0226131
  Y          0.2240202          -0.0714723             0.9719602
  Z          0.8924536          -0.3856742            -0.2340555

Notes:  (1) The A matrix conforms to the "SAI" spin Hamiltonian convention.
        (2) Tensor is right-handed.

 -----------------------------------------------------------
 Nucleus  64C : A:ISTP=   13 I=  0.5 P=134.1903 MHz/au**3
                Q:ISTP=   13 I=  0.5 Q=  0.0000 barn
 -----------------------------------------------------------
 Raw HFC matrix (all values in MHz):
 -----------------------------------
               0.1103             -0.0574               -0.0060
              -0.0574              0.3063                0.3307
              -0.0060              0.3307                1.2112

 A(FC)         0.5426              0.5426                0.5426
 A(SD)        -0.4573             -0.3197                0.7771
            ----------          ----------            ----------
 A(Tot)        0.0852              0.2229                1.3197    A(iso)=    0.5426
 Orientation:
  X          0.9062454          -0.4223017            -0.0195071
  Y          0.4069037           0.8588269             0.3112005
  Z         -0.1146673          -0.2899616             0.9501440

Notes:  (1) The A matrix conforms to the "SAI" spin Hamiltonian convention.
        (2) Tensor is right-handed.

 -----------------------------------------------------------
 Nucleus  65C : A:ISTP=   13 I=  0.5 P=134.1903 MHz/au**3
                Q:ISTP=   13 I=  0.5 Q=  0.0000 barn
 -----------------------------------------------------------
 Raw HFC matrix (all values in MHz):
 -----------------------------------
               0.0784             -0.0345               -0.0408
              -0.0345             -0.0068               -0.2018
              -0.0408             -0.2018               -0.8045

 A(FC)        -0.2443             -0.2443               -0.2443
 A(SD)         0.2743              0.3365               -0.6108
            ----------          ----------            ----------
 A(Tot)        0.0300              0.0922               -0.8550    A(iso)=   -0.2443
 Orientation:
  X          0.4265006           0.9030474            -0.0510165
  Y          0.8741754          -0.4260309            -0.2330558
  Z         -0.2321951           0.0548011            -0.9711242

Notes:  (1) The A matrix conforms to the "SAI" spin Hamiltonian convention.
        (2) Tensor is right-handed.

 -----------------------------------------------------------
 Nucleus  68C : A:ISTP=   13 I=  0.5 P=134.1903 MHz/au**3
                Q:ISTP=   13 I=  0.5 Q=  0.0000 barn
```

```
 -----------------------------------------------------------
 Raw HFC matrix (all values in MHz):
 -----------------------------------
                0.7046              -0.0779              -0.0729
               -0.0779               0.7360               0.3490
               -0.0729               0.3490               1.3592

 A(FC)          0.9333               0.9333               0.9333
 A(SD)         -0.3669              -0.2271               0.5940
            ----------           ----------           ----------
 A(Tot)         0.5664               0.7061               1.5273    A(iso)=    0.9333
 Orientation:
  X          0.3069719            0.9442536            0.1189679
  Y          0.8811594           -0.2347500           -0.4104273
  Z         -0.3596197            0.2308193           -0.9040996

Notes:  (1) The A matrix conforms to the "SAI" spin Hamiltonian convention.
        (2) Tensor is right-handed.

 -----------------------------------------------------------
 Nucleus  69C : A:ISTP=   13 I=  0.5 P=134.1903 MHz/au**3
                Q:ISTP=   13 I=  0.5 Q=  0.0000 barn
 -----------------------------------------------------------
 Raw HFC matrix (all values in MHz):
 -----------------------------------
                0.2119              -0.1827               0.1559
               -0.1827               0.0837              -0.2995
                0.1559              -0.2995              -1.0663

 A(FC)         -0.2569              -0.2569              -0.2569
 A(SD)          0.2296               0.6619              -0.8915
            ----------           ----------           ----------
 A(Tot)        -0.0273               0.4050              -1.1484    A(iso)=   -0.2569
 Orientation:
  X          0.6503923           -0.7552466           -0.0811933
  Y          0.7502699            0.6220240            0.2240119
  Z         -0.1186800           -0.2066125            0.9711984

Notes:  (1) The A matrix conforms to the "SAI" spin Hamiltonian convention.
        (2) Tensor is right-handed.

 -----------------------------------------------------------
 Nucleus  70C : A:ISTP=   13 I=  0.5 P=134.1903 MHz/au**3
                Q:ISTP=   13 I=  0.5 Q=  0.0000 barn
 -----------------------------------------------------------
 Raw HFC matrix (all values in MHz):
 -----------------------------------
                9.3055              -1.1132              -0.7138
               -1.1132              10.2056               1.7105
               -0.7138               1.7105              10.1983

 A(FC)          9.9031               9.9031               9.9031
 A(SD)         -1.5684              -0.9758               2.5442
            ----------           ----------           ----------
 A(Tot)         8.3348               8.9273              12.4473    A(iso)=    9.9031
 Orientation:
  X         -0.4795529            0.7898553           -0.3823057
  Y         -0.7302421           -0.1176201            0.6729873
  Z          0.4865957            0.6019088            0.6331906

Notes:  (1) The A matrix conforms to the "SAI" spin Hamiltonian convention.
        (2) Tensor is right-handed.

 -----------------------------------------------------------
 Nucleus  71C : A:ISTP=   13 I=  0.5 P=134.1903 MHz/au**3
                Q:ISTP=   13 I=  0.5 Q=  0.0000 barn
```

```
 -----------------------------------------------------------
 Raw HFC matrix (all values in MHz):
 -----------------------------------
               0.8502            0.0421            -0.0510
               0.0421            0.4483            -0.2141
              -0.0510           -0.2141            -0.6163

 A(FC)         0.2274            0.2274             0.2274
 A(SD)         0.2553           -0.8863             0.6310
            ----------        ----------         ----------
 A(Tot)        0.4827           -0.6589             0.8584    A(iso)=    0.2274
 Orientation:
  X         -0.1369709         0.0278961          0.9901822
  Y          0.9734726         0.1887370          0.1293423
  Z         -0.1832759         0.9816314         -0.0530075

Notes:  (1) The A matrix conforms to the "SAI" spin Hamiltonian convention.
        (2) Tensor is right-handed.

 -----------------------------------------------------------
 Nucleus  74C : A:ISTP=   13 I=  0.5 P=134.1903 MHz/au**3
                Q:ISTP=   13 I=  0.5 Q=  0.0000 barn
 -----------------------------------------------------------
 Raw HFC matrix (all values in MHz):
 -----------------------------------
              -8.2099           -0.0246            -0.0236
              -0.0246           -9.0087            -0.8313
              -0.0236           -0.8313           -11.6923

 A(FC)        -9.6370           -9.6370            -9.6370
 A(SD)         1.4278            0.8644            -2.2922
            ----------        ----------         ----------
 A(Tot)       -8.2091           -8.7726           -11.9292    A(iso)=   -9.6370
 Orientation:
  X          0.9995006         0.0305880          0.0079284
  Y         -0.0315898         0.9612641          0.2738127
  Z          0.0007541        -0.2739264          0.9617504

Notes:  (1) The A matrix conforms to the "SAI" spin Hamiltonian convention.
        (2) Tensor is right-handed.

 -----------------------------------------------------------
 Nucleus  75C : A:ISTP=   13 I=  0.5 P=134.1903 MHz/au**3
                Q:ISTP=   13 I=  0.5 Q=  0.0000 barn
 -----------------------------------------------------------
 Raw HFC matrix (all values in MHz):
 -----------------------------------
              -0.9806           -0.0120            -0.0155
              -0.0120           -1.1476             0.9906
              -0.0155            0.9906             4.5046

 A(FC)         0.7922            0.7922             0.7922
 A(SD)        -1.7725           -2.1086             3.8811
            ----------        ----------         ----------
 A(Tot)       -0.9804           -1.3164             4.6732    A(iso)=    0.7922
 Orientation:
  X          0.9996159         0.0275457         -0.0030560
  Y         -0.0266429         0.9854661          0.1677699
  Z          0.0076330        -0.1676240          0.9858215

Notes:  (1) The A matrix conforms to the "SAI" spin Hamiltonian convention.
        (2) Tensor is right-handed.

 -----------------------------------------------------------
 Nucleus  76C : A:ISTP=   13 I=  0.5 P=134.1903 MHz/au**3
                Q:ISTP=   13 I=  0.5 Q=  0.0000 barn
```

```
 ----------------------------------------------------------
 Raw HFC matrix (all values in MHz):
 -----------------------------------
              9.3739              1.1563              0.8128
              1.1563              9.9973              1.6618
              0.8128              1.6618             10.1261

 A(FC)        9.8324              9.8324              9.8324
 A(SD)       -1.5660             -0.9726              2.5385
            ----------          ----------          ----------
 A(Tot)       8.2665              8.8599             12.3710    A(iso)=    9.8324
 Orientation:
  X         -0.4332522          -0.7963434          0.4220542
  Y          0.7578291          -0.0684107          0.6488567
  Z         -0.4878398           0.6009635          0.6331313

Notes:  (1) The A matrix conforms to the "SAI" spin Hamiltonian convention.
        (2) Tensor is right-handed.

 ----------------------------------------------------------
 Nucleus  77C : A:ISTP=   13 I=  0.5 P=134.1903 MHz/au**3
                Q:ISTP=   13 I=  0.5 Q=  0.0000 barn
 ----------------------------------------------------------
 Raw HFC matrix (all values in MHz):
 -----------------------------------
              0.8283             -0.0637              0.0496
             -0.0637              0.4352             -0.2202
              0.0496             -0.2202             -0.6306

 A(FC)        0.2110              0.2110              0.2110
 A(SD)        0.2537             -0.8862              0.6325
            ----------          ----------          ----------
 A(Tot)       0.4646             -0.6752              0.8435    A(iso)=    0.2110
 Orientation:
  X          0.1941148          -0.0241971          0.9806803
  Y          0.9634015           0.1930996         -0.1859302
  Z         -0.1848700           0.9808808          0.0607950

Notes:  (1) The A matrix conforms to the "SAI" spin Hamiltonian convention.
        (2) Tensor is right-handed.

 ----------------------------------------------------------
 Nucleus  78C : A:ISTP=   13 I=  0.5 P=134.1903 MHz/au**3
                Q:ISTP=   13 I=  0.5 Q=  0.0000 barn
 ----------------------------------------------------------
 Raw HFC matrix (all values in MHz):
 -----------------------------------
              0.2621              0.1749             -0.1715
              0.1749              0.0841             -0.2900
             -0.1715             -0.2900             -1.0437

 A(FC)       -0.2325             -0.2325             -0.2325
 A(SD)        0.2288              0.6642             -0.8930
            ----------          ----------          ----------
 A(Tot)      -0.0037              0.4317             -1.1255    A(iso)=   -0.2325
 Orientation:
  X          0.5991971           0.7952513          0.0924022
  Y         -0.7912797           0.5707014          0.2194911
  Z          0.1218165          -0.2046344          0.9712289

Notes:  (1) The A matrix conforms to the "SAI" spin Hamiltonian convention.
        (2) Tensor is right-handed.

 ----------------------------------------------------------
 Nucleus  79C : A:ISTP=   13 I=  0.5 P=134.1903 MHz/au**3
                Q:ISTP=   13 I=  0.5 Q=  0.0000 barn
```

```
 -----------------------------------------------------------
 Raw HFC matrix (all values in MHz):
 -----------------------------------
               0.7222             0.0792             0.0933
               0.0792             0.7374             0.3477
               0.0933             0.3477             1.3697

 A(FC)         0.9431             0.9431             0.9431
 A(SD)        -0.3686            -0.2287             0.5972
             ----------         ----------         ----------
 A(Tot)        0.5745             0.7144             1.5403    A(iso)=    0.9431
 Orientation:
  X           -0.2521036          0.9571883         -0.1422473
  Y            0.8971505          0.1760876         -0.4051101
  Z           -0.3627186         -0.2297470         -0.9031343

Notes:  (1) The A matrix conforms to the "SAI" spin Hamiltonian convention.
        (2) Tensor is right-handed.

 -----------------------------------------------------------
 Nucleus  81C : A:ISTP=   13 I=  0.5 P=134.1903 MHz/au**3
                Q:ISTP=   13 I=  0.5 Q=  0.0000 barn
 -----------------------------------------------------------
 Raw HFC matrix (all values in MHz):
 -----------------------------------
               0.1183             0.0679             0.0221
               0.0679             0.2995             0.3313
               0.0221             0.3313             1.2142

 A(FC)         0.5440             0.5440             0.5440
 A(SD)        -0.4585            -0.3206             0.7790
             ----------         ----------         ----------
 A(Tot)        0.0855             0.2234             1.3230    A(iso)=    0.5440
 Orientation:
  X            0.8786202          0.4762411          0.0349419
  Y           -0.4625768          0.8306785          0.3098320
  Z            0.1185292         -0.2883879          0.9501491

Notes:  (1) The A matrix conforms to the "SAI" spin Hamiltonian convention.
        (2) Tensor is right-handed.

 -----------------------------------------------------------
 Nucleus  82C : A:ISTP=   13 I=  0.5 P=134.1903 MHz/au**3
                Q:ISTP=   13 I=  0.5 Q=  0.0000 barn
 -----------------------------------------------------------
 Raw HFC matrix (all values in MHz):
 -----------------------------------
               0.0819             0.0300             0.0331
               0.0300            -0.0131            -0.2046
               0.0331            -0.2046            -0.8044

 A(FC)        -0.2452            -0.2452            -0.2452
 A(SD)         0.2736             0.3370            -0.6106
             ----------         ----------         ----------
 A(Tot)        0.0284             0.0918            -0.8559    A(iso)=   -0.2452
 Orientation:
  X           -0.3603542          0.9318774          0.0418255
  Y            0.9024614          0.3596227         -0.2371389
  Z           -0.2360258         -0.0477081         -0.9705750

Notes:  (1) The A matrix conforms to the "SAI" spin Hamiltonian convention.
        (2) Tensor is right-handed.

 -----------------------------------------------------------
 Nucleus   9H : A:ISTP=    1 I=  0.5 P=533.5514 MHz/au**3
                Q:ISTP=    2 I=  1.0 Q=  0.0029 barn
```

```
 -----------------------------------------------------------
 Raw HFC matrix (all values in MHz):
 -----------------------------------
               0.8827              -0.0775              0.0419
              -0.0775               0.4729             -0.5882
               0.0419              -0.5882              0.1818

 A(FC)         0.5125               0.5125              0.5125
 A(SD)        -0.7913               0.3052              0.4861
             ----------           ----------          ----------
 A(Tot)       -0.2788               0.8177              0.9985    A(iso)=    0.5125
 Orientation:
  X          0.0128109           0.7994121          -0.6006465
  Y          0.6170216           0.4663714           0.6338629
  Z          0.7868420          -0.3787322          -0.4872799

Notes:  (1) The A matrix conforms to the "SAI" spin Hamiltonian convention.
        (2) Tensor is right-handed.

 -----------------------------------------------------------
 Nucleus  10H : A:ISTP=    1 I=  0.5 P=533.5514 MHz/au**3
                Q:ISTP=    2 I=  1.0 Q=  0.0029 barn
 -----------------------------------------------------------
 Raw HFC matrix (all values in MHz):
 -----------------------------------
              -4.8664              -2.3217              1.7956
              -2.3217              -4.7912             -0.2672
               1.7956              -0.2672             -4.9765

 A(FC)        -4.8780              -4.8780             -4.8780
 A(SD)         3.0819              -0.2877             -2.7942
             ----------           ----------          ----------
 A(Tot)       -1.7961              -5.1657             -7.6723    A(iso)=   -4.8780
 Orientation:
  X          0.6909980           0.0078160          -0.7228145
  Y         -0.5747432           0.6123838          -0.5428225
  Z          0.4383971           0.7905220           0.4276482

Notes:  (1) The A matrix conforms to the "SAI" spin Hamiltonian convention.
        (2) Tensor is right-handed.

 -----------------------------------------------------------
 Nucleus  11H : A:ISTP=    1 I=  0.5 P=533.5514 MHz/au**3
                Q:ISTP=    2 I=  1.0 Q=  0.0029 barn
 -----------------------------------------------------------
 Raw HFC matrix (all values in MHz):
 -----------------------------------
               1.2372              -0.2865              0.2015
              -0.2865               0.8955             -0.5130
               0.2015              -0.5130              0.6234

 A(FC)         0.9187               0.9187              0.9187
 A(SD)        -0.6902              -0.0057              0.6959
             ----------           ----------          ----------
 A(Tot)        0.2285               0.9129              1.6146    A(iso)=    0.9187
 Orientation:
  X          0.0167860           0.7330496          -0.6799680
  Y          0.6140738           0.5291354           0.5856016
  Z          0.7890701          -0.4273804          -0.4412645

Notes:  (1) The A matrix conforms to the "SAI" spin Hamiltonian convention.
        (2) Tensor is right-handed.

 -----------------------------------------------------------
 Nucleus  12H : A:ISTP=    1 I=  0.5 P=533.5514 MHz/au**3
                Q:ISTP=    2 I=  1.0 Q=  0.0029 barn
```

```
 ------------------------------------------------------------
 Raw HFC matrix (all values in MHz):
 -----------------------------------
               -2.8257            1.1963            -0.8974
                1.1963           -5.6890             0.1362
               -0.8974            0.1362            -5.5426

 A(FC)         -4.6858           -4.6858            -4.6858
 A(SD)          2.4869           -0.7779            -1.7090
             ----------        ----------         ----------
 A(Tot)        -2.1989           -5.4637            -6.3948    A(iso)=   -4.6858
 Orientation:
  X         -0.9222481         0.0158289         -0.3862744
  Y         -0.3069615         0.5774185          0.7565465
  Z          0.2350173         0.8162949         -0.5276642

Notes:  (1) The A matrix conforms to the "SAI" spin Hamiltonian convention.
        (2) Tensor is right-handed.

 ------------------------------------------------------------
 Nucleus  17H : A:ISTP=    1 I=  0.5 P=533.5514 MHz/au**3
                Q:ISTP=    2 I=  1.0 Q=  0.0029 barn
 ------------------------------------------------------------
 Raw HFC matrix (all values in MHz):
 -----------------------------------
                0.8861            0.0750            -0.0452
                0.0750            0.4654            -0.5847
               -0.0452           -0.5847             0.1763

 A(FC)          0.5093            0.5093             0.5093
 A(SD)         -0.7909            0.3065             0.4843
             ----------        ----------         ----------
 A(Tot)        -0.2816            0.8158             0.9936    A(iso)=    0.5093
 Orientation:
  X         -0.0091791        -0.7770676         -0.6293503
  Y          0.6169674         0.4908985         -0.6151178
  Z          0.7869352        -0.3939348          0.4749193

Notes:  (1) The A matrix conforms to the "SAI" spin Hamiltonian convention.
        (2) Tensor is right-handed.

 ------------------------------------------------------------
 Nucleus  18H : A:ISTP=    1 I=  0.5 P=533.5514 MHz/au**3
                Q:ISTP=    2 I=  1.0 Q=  0.0029 barn
 ------------------------------------------------------------
 Raw HFC matrix (all values in MHz):
 -----------------------------------
               -4.6766            2.3214            -1.7936
                2.3214           -4.9069            -0.1811
               -1.7936           -0.1811            -5.0296

 A(FC)         -4.8710           -4.8710            -4.8710
 A(SD)          3.0780           -0.2879            -2.7901
             ----------        ----------         ----------
 A(Tot)        -1.7930           -5.1590            -7.6611    A(iso)=   -4.8710
 Orientation:
  X          0.7131589        -0.0047681          0.7009862
  Y          0.5564608         0.6120060         -0.5619609
  Z         -0.4263283         0.7908388          0.4391108

Notes:  (1) The A matrix conforms to the "SAI" spin Hamiltonian convention.
        (2) Tensor is right-handed.

 ------------------------------------------------------------
 Nucleus  19H : A:ISTP=    1 I=  0.5 P=533.5514 MHz/au**3
                Q:ISTP=    2 I=  1.0 Q=  0.0029 barn
```

```
 -----------------------------------------------------------
 Raw HFC matrix (all values in MHz):
 ----------------------------------
               1.2562              0.2851              -0.2046
               0.2851              0.8784              -0.5027
              -0.2046             -0.5027               0.6139

 A(FC)         0.9162              0.9162               0.9162
 A(SD)        -0.6900             -0.0057               0.6957
            ----------          ----------           ----------
 A(Tot)        0.2262              0.9105               1.6118    A(iso)=    0.9162
 Orientation:
  X         -0.0131628          -0.7118476          -0.7022106
  Y          0.6140155           0.5485152          -0.5675526
  Z          0.7891842          -0.4386388           0.4298655

Notes:  (1) The A matrix conforms to the "SAI" spin Hamiltonian convention.
        (2) Tensor is right-handed.

 -----------------------------------------------------------
 Nucleus  20H : A:ISTP=    1 I=  0.5 P=533.5514 MHz/au**3
                Q:ISTP=    2 I=  1.0 Q=  0.0029 barn
 -----------------------------------------------------------
 Raw HFC matrix (all values in MHz):
 ----------------------------------
              -2.9176             -1.2745               0.9399
              -1.2745             -5.6205               0.0905
               0.9399              0.0905              -5.5069

 A(FC)        -4.6817             -4.6817              -4.6817
 A(SD)         2.4864             -0.7781              -1.7083
            ----------          ----------           ----------
 A(Tot)       -2.1953             -5.4598              -6.3900    A(iso)=   -4.6817
 Orientation:
  X          0.9098050          -0.0143509          -0.4147878
  Y         -0.3319310           0.5747955          -0.7479519
  Z          0.2491519           0.8181713           0.5181883

Notes:  (1) The A matrix conforms to the "SAI" spin Hamiltonian convention.
        (2) Tensor is right-handed.

 -----------------------------------------------------------
 Nucleus  21H : A:ISTP=    1 I=  0.5 P=533.5514 MHz/au**3
                Q:ISTP=    2 I=  1.0 Q=  0.0029 barn
 -----------------------------------------------------------
 Raw HFC matrix (all values in MHz):
 ----------------------------------
               0.8810             -0.0621              -0.0607
              -0.0621              0.1836               0.5887
              -0.0607              0.5887               0.4672

 A(FC)         0.5106              0.5106               0.5106
 A(SD)        -0.7909              0.3058               0.4850
            ----------          ----------           ----------
 A(Tot)       -0.2803              0.8164               0.9956    A(iso)=    0.5106
 Orientation:
  X         -0.0097254           0.7991916          -0.6009976
  Y         -0.7859403           0.3654782           0.4987219
  Z          0.6182259           0.4771986           0.6245625

Notes:  (1) The A matrix conforms to the "SAI" spin Hamiltonian convention.
        (2) Tensor is right-handed.

 -----------------------------------------------------------
 Nucleus  23H : A:ISTP=    1 I=  0.5 P=533.5514 MHz/au**3
                Q:ISTP=    2 I=  1.0 Q=  0.0029 barn
```

```
 -----------------------------------------------------------
 Raw HFC matrix (all values in MHz):
 -----------------------------------
              -4.8780                -1.8005                -2.3121
              -1.8005                -4.8995                 0.2923
              -2.3121                 0.2923                -4.8386

 A(FC)        -4.8721                -4.8721                -4.8721
 A(SD)         3.0785                -0.2878                -2.7907
            ----------             ----------             ----------
 A(Tot)       -1.7935                -5.1599                -7.6628    A(iso)=   -4.8721
 Orientation:
  X         -0.6887467             -0.0146482              0.7248540
  Y          0.4525592             -0.7897773              0.4140556
  Z          0.5664081              0.6132188              0.5505857

Notes:  (1) The A matrix conforms to the "SAI" spin Hamiltonian convention.
        (2) Tensor is right-handed.

 -----------------------------------------------------------
 Nucleus  25H : A:ISTP=    1 I=  0.5 P=533.5514 MHz/au**3
                Q:ISTP=    2 I=  1.0 Q=  0.0029 barn
 -----------------------------------------------------------
 Raw HFC matrix (all values in MHz):
 -----------------------------------
               0.8866                 0.0252                 0.0921
               0.0252                 0.1782                 0.5842
               0.0921                 0.5842                 0.4684

 A(FC)         0.5111                 0.5111                 0.5111
 A(SD)        -0.7909                 0.3054                 0.4855
            ----------             ----------             ----------
 A(Tot)       -0.2798                 0.8165                 0.9966    A(iso)=    0.5111
 Orientation:
  X          0.0317592              0.7768178             -0.6289240
  Y          0.7859157             -0.4081728             -0.4644691
  Z         -0.6175176             -0.4795301             -0.6234765

Notes:  (1) The A matrix conforms to the "SAI" spin Hamiltonian convention.
        (2) Tensor is right-handed.

 -----------------------------------------------------------
 Nucleus  30H : A:ISTP=    1 I=  0.5 P=533.5514 MHz/au**3
                Q:ISTP=    2 I=  1.0 Q=  0.0029 barn
 -----------------------------------------------------------
 Raw HFC matrix (all values in MHz):
 -----------------------------------
              -4.6618                 1.7882                 2.3261
               1.7882                -5.1026                 0.1557
               2.3261                 0.1557                -4.8583

 A(FC)        -4.8742                -4.8742                -4.8742
 A(SD)         3.0797                -0.2877                -2.7921
            ----------             ----------             ----------
 A(Tot)       -1.7945                -5.1619                -7.6663    A(iso)=   -4.8742
 Orientation:
  X         -0.7150963             -0.0271477             -0.6984986
  Y         -0.4130824             -0.7896954              0.4535902
  Z         -0.5639151              0.6128981              0.5534941

Notes:  (1) The A matrix conforms to the "SAI" spin Hamiltonian convention.
        (2) Tensor is right-handed.

 -----------------------------------------------------------
 Nucleus  32H : A:ISTP=    1 I=  0.5 P=533.5514 MHz/au**3
                Q:ISTP=    2 I=  1.0 Q=  0.0029 barn
```

```
 -----------------------------------------------------------
 Raw HFC matrix (all values in MHz):
 -----------------------------------
               1.2335              -0.2198              -0.2718
              -0.2198               0.6311               0.5161
              -0.2718               0.5161               0.8859

 A(FC)         0.9168               0.9168               0.9168
 A(SD)        -0.6900              -0.0056               0.6956
            ----------           ----------           ----------
 A(Tot)        0.2269               0.9112               1.6124    A(iso)=    0.9168
 Orientation:
  X          0.0060744            0.7350681            0.6779661
  Y          0.7884771            0.4134669           -0.4553559
  Z         -0.6150341            0.5373267           -0.5770728

Notes:  (1) The A matrix conforms to the "SAI" spin Hamiltonian convention.
        (2) Tensor is right-handed.

 -----------------------------------------------------------
 Nucleus  39H : A:ISTP=    1 I=  0.5 P=533.5514 MHz/au**3
                Q:ISTP=    2 I=  1.0 Q=  0.0029 barn
 -----------------------------------------------------------
 Raw HFC matrix (all values in MHz):
 -----------------------------------
               1.2586               0.1864               0.2993
               0.1864               0.6090               0.4994
               0.2993               0.4994               0.8854

 A(FC)         0.9177               0.9177               0.9177
 A(SD)        -0.6898              -0.0059               0.6958
            ----------           ----------           ----------
 A(Tot)        0.2278               0.9117               1.6134    A(iso)=    0.9177
 Orientation:
  X          0.0360281            0.7093440           -0.7039411
  Y          0.7878720           -0.4534957           -0.4166526
  Z         -0.6147843           -0.5396043           -0.5752108

Notes:  (1) The A matrix conforms to the "SAI" spin Hamiltonian convention.
        (2) Tensor is right-handed.

 -----------------------------------------------------------
 Nucleus  40H : A:ISTP=    1 I=  0.5 P=533.5514 MHz/au**3
                Q:ISTP=    2 I=  1.0 Q=  0.0029 barn
 -----------------------------------------------------------
 Raw HFC matrix (all values in MHz):
 -----------------------------------
              -2.8149               0.8439               1.2260
               0.8439              -5.5735              -0.1503
               1.2260              -0.1503              -5.6565

 A(FC)        -4.6817              -4.6817              -4.6817
 A(SD)         2.4864              -0.7780              -1.7084
            ----------           ----------           ----------
 A(Tot)       -2.1953              -5.4596              -6.3901    A(iso)=   -4.6817
 Orientation:
  X          0.9231809            0.0072785           -0.3842967
  Y          0.2164991            0.8162824            0.5355476
  Z          0.3175926           -0.5776073            0.7520005

Notes:  (1) The A matrix conforms to the "SAI" spin Hamiltonian convention.
        (2) Tensor is right-handed.

 -----------------------------------------------------------
 Nucleus  41H : A:ISTP=    1 I=  0.5 P=533.5514 MHz/au**3
                Q:ISTP=    2 I=  1.0 Q=  0.0029 barn
```

```
 -----------------------------------------------------------
 Raw HFC matrix (all values in MHz):
 -----------------------------------
               -2.9303              -0.9935              -1.2432
               -0.9935              -5.4687              -0.0765
               -1.2432              -0.0765              -5.6496

 A(FC)         -4.6829              -4.6829              -4.6829
 A(SD)          2.4856              -0.7774              -1.7082
             ----------           ----------           ----------
 A(Tot)        -2.1973              -5.4603              -6.3910    A(iso)=   -4.6829
 Orientation:
  X          0.9082340           -0.0374863           -0.4167802
  Y         -0.2683154           -0.8164620           -0.5112696
  Z         -0.3211196            0.5761810           -0.7515967

Notes:  (1) The A matrix conforms to the "SAI" spin Hamiltonian convention.
        (2) Tensor is right-handed.

 -----------------------------------------------------------
 Nucleus  56H : A:ISTP=    1 I=  0.5 P=533.5514 MHz/au**3
                Q:ISTP=    2 I=  1.0 Q=  0.0029 barn
 -----------------------------------------------------------
 Raw HFC matrix (all values in MHz):
 -----------------------------------
               -0.8374              -0.1703              -0.9362
               -0.1703              -1.0378               0.6911
               -0.9362               0.6911               2.3488

 A(FC)          0.1579               0.1579               0.1579
 A(SD)         -1.2466              -1.3331               2.5798
             ----------           ----------           ----------
 A(Tot)        -1.0888              -1.1753               2.7376    A(iso)=    0.1579
 Orientation:
  X         -0.9556511            0.1434708           -0.2571906
  Y         -0.1944638           -0.9632619            0.1852306
  Z         -0.2211667            0.2270301            0.9484422

Notes:  (1) The A matrix conforms to the "SAI" spin Hamiltonian convention.
        (2) Tensor is right-handed.

 -----------------------------------------------------------
 Nucleus  57H : A:ISTP=    1 I=  0.5 P=533.5514 MHz/au**3
                Q:ISTP=    2 I=  1.0 Q=  0.0029 barn
 -----------------------------------------------------------
 Raw HFC matrix (all values in MHz):
 -----------------------------------
               -0.4516              -0.0571               0.0245
               -0.0571              -0.4766               0.1527
                0.0245               0.1527               0.1450

 A(FC)         -0.2611              -0.2611              -0.2611
 A(SD)          0.4417              -0.1527              -0.2890
             ----------           ----------           ----------
 A(Tot)         0.1807              -0.4137              -0.5501    A(iso)=   -0.2611
 Orientation:
  X          0.0174548           -0.8490515           -0.5280216
  Y          0.2247507            0.5179189           -0.8253769
  Z          0.9742600           -0.1042665            0.1998651

Notes:  (1) The A matrix conforms to the "SAI" spin Hamiltonian convention.
        (2) Tensor is right-handed.

 -----------------------------------------------------------
 Nucleus  58H : A:ISTP=    1 I=  0.5 P=533.5514 MHz/au**3
                Q:ISTP=    2 I=  1.0 Q=  0.0029 barn
```

```
 -----------------------------------------------------------
 Raw HFC matrix (all values in MHz):
 -----------------------------------
                0.4117               -0.0028                0.0381
               -0.0028                0.0194                0.0807
                0.0381                0.0807                0.3090

 A(FC)          0.2467                0.2467                0.2467
 A(SD)         -0.2487                0.0696                0.1791
            ----------            ----------            ----------
 A(Tot)        -0.0020                0.3163                0.4258    A(iso)=    0.2467
 Orientation:
  X          0.0299000            -0.3533308             0.9350205
  Y          0.9666318             0.2483208             0.0629260
  Z         -0.2544187             0.9019391             0.3489656

Notes:  (1) The A matrix conforms to the "SAI" spin Hamiltonian convention.
        (2) Tensor is right-handed.

 -----------------------------------------------------------
 Nucleus  59H : A:ISTP=    1 I=  0.5 P=533.5514 MHz/au**3
                Q:ISTP=    2 I=  1.0 Q=  0.0029 barn
 -----------------------------------------------------------
 Raw HFC matrix (all values in MHz):
 -----------------------------------
                0.2788               -0.0182                0.0853
               -0.0182               -0.6970                0.0930
                0.0853                0.0930               -0.1509

 A(FC)         -0.1897               -0.1897               -0.1897
 A(SD)          0.0389                0.4848               -0.5237
            ----------            ----------            ----------
 A(Tot)        -0.1508                0.2952               -0.7134    A(iso)=   -0.1897
 Orientation:
  X         -0.1850292            -0.9821964            -0.0324725
  Y          0.1709764             0.0003652            -0.9852751
  Z          0.9677455            -0.1878567             0.1678649

Notes:  (1) The A matrix conforms to the "SAI" spin Hamiltonian convention.
        (2) Tensor is right-handed.

 -----------------------------------------------------------
 Nucleus  60H : A:ISTP=    1 I=  0.5 P=533.5514 MHz/au**3
                Q:ISTP=    2 I=  1.0 Q=  0.0029 barn
 -----------------------------------------------------------
 Raw HFC matrix (all values in MHz):
 -----------------------------------
               -0.8613                0.1503                0.9141
                0.1503               -1.0496                0.6948
                0.9141                0.6948                2.3706

 A(FC)          0.1532                0.1532                0.1532
 A(SD)         -1.2446               -1.3463                2.5909
            ----------            ----------            ----------
 A(Tot)        -1.0914               -1.1931                2.7442    A(iso)=    0.1532
 Orientation:
  X         -0.9310555            -0.2669430             0.2487508
  Y          0.3160127            -0.9307414             0.1840011
  Z          0.1824049             0.2499237             0.9509293

Notes:  (1) The A matrix conforms to the "SAI" spin Hamiltonian convention.
        (2) Tensor is right-handed.

 -----------------------------------------------------------
 Nucleus  61H : A:ISTP=    1 I=  0.5 P=533.5514 MHz/au**3
                Q:ISTP=    2 I=  1.0 Q=  0.0029 barn
```

```
 ----------------------------------------------------------
 Raw HFC matrix (all values in MHz):
 -----------------------------------
               -0.4512              0.0530             -0.0321
                0.0530             -0.4799              0.1588
               -0.0321              0.1588              0.1439

 A(FC)         -0.2624             -0.2624             -0.2624
 A(SD)          0.4450             -0.1549             -0.2901
            ----------          ----------          ----------
 A(Tot)         0.1826             -0.4173             -0.5525    A(iso)=   -0.2624
 Orientation:
  X         -0.0299128           0.8625961          -0.5050082
  Y          0.2307897           0.4975427           0.8361743
  Z          0.9725438          -0.0915384          -0.2139611

Notes:  (1) The A matrix conforms to the "SAI" spin Hamiltonian convention.
        (2) Tensor is right-handed.

 ----------------------------------------------------------
 Nucleus  62H : A:ISTP=    1 I=  0.5 P=533.5514 MHz/au**3
               Q:ISTP=    2 I=  1.0 Q=  0.0029 barn
 ----------------------------------------------------------
 Raw HFC matrix (all values in MHz):
 -----------------------------------
                0.2689             -0.0309             -0.0734
               -0.0309             -0.6965              0.1024
               -0.0734              0.1024             -0.1594

 A(FC)         -0.1957             -0.1957             -0.1957
 A(SD)          0.0409              0.4791             -0.5200
            ----------          ----------          ----------
 A(Tot)        -0.1548              0.2835             -0.7157    A(iso)=   -0.1957
 Orientation:
  X          0.1804082           0.9834351           0.0175581
  Y          0.1727586          -0.0492555           0.9837319
  Z          0.9683013          -0.1744400          -0.1787830

Notes:  (1) The A matrix conforms to the "SAI" spin Hamiltonian convention.
        (2) Tensor is right-handed.

 ----------------------------------------------------------
 Nucleus  63H : A:ISTP=    1 I=  0.5 P=533.5514 MHz/au**3
               Q:ISTP=    2 I=  1.0 Q=  0.0029 barn
 ----------------------------------------------------------
 Raw HFC matrix (all values in MHz):
 -----------------------------------
                0.4090             -0.0162             -0.0309
               -0.0162              0.0226              0.0850
               -0.0309              0.0850              0.3095

 A(FC)          0.2470              0.2470              0.2470
 A(SD)         -0.2479              0.0728              0.1751
            ----------          ----------          ----------
 A(Tot)        -0.0008              0.3198              0.4221    A(iso)=    0.2470
 Orientation:
  X          0.0184061          -0.3564670          -0.9341266
  Y          0.9647455          -0.2389967           0.1102116
  Z         -0.2625400          -0.9032230           0.3395009

Notes:  (1) The A matrix conforms to the "SAI" spin Hamiltonian convention.
        (2) Tensor is right-handed.

 ----------------------------------------------------------
 Nucleus  66H : A:ISTP=    1 I=  0.5 P=533.5514 MHz/au**3
               Q:ISTP=    2 I=  1.0 Q=  0.0029 barn
```

```
 -----------------------------------------------------------
 Raw HFC matrix (all values in MHz):
 ----------------------------------
               -0.4520            0.0470            0.0508
                0.0470            0.1405           -0.1569
                0.0508           -0.1569           -0.4801

 A(FC)         -0.2639           -0.2639           -0.2639
 A(SD)          0.4436           -0.1537           -0.2899
            ----------        ----------        ----------
 A(Tot)         0.1798           -0.4176           -0.5538    A(iso)=   -0.2639
 Orientation:
  X          -0.0540572         0.8552259        -0.5154284
  Y          -0.9723671         0.0723180         0.2219738
  Z           0.2271125         0.5131849         0.8276842

Notes:  (1) The A matrix conforms to the "SAI" spin Hamiltonian convention.
        (2) Tensor is right-handed.

 -----------------------------------------------------------
 Nucleus  67H : A:ISTP=    1 I=  0.5 P=533.5514 MHz/au**3
                Q:ISTP=    2 I=  1.0 Q=  0.0029 barn
 -----------------------------------------------------------
 Raw HFC matrix (all values in MHz):
 ----------------------------------
                0.4123            0.0319           -0.0074
                0.0319            0.3093           -0.0818
               -0.0074           -0.0818            0.0221

 A(FC)          0.2479            0.2479            0.2479
 A(SD)         -0.2475            0.0716            0.1759
            ----------        ----------        ----------
 A(Tot)         0.0004            0.3194            0.4238    A(iso)=    0.2479
 Orientation:
  X          -0.0023687        -0.3324152        -0.9431302
  Y           0.2562432         0.9114419        -0.3218899
  Z           0.9666094        -0.2424331         0.0830202

Notes:  (1) The A matrix conforms to the "SAI" spin Hamiltonian convention.
        (2) Tensor is right-handed.

 -----------------------------------------------------------
 Nucleus  72H : A:ISTP=    1 I=  0.5 P=533.5514 MHz/au**3
                Q:ISTP=    2 I=  1.0 Q=  0.0029 barn
 -----------------------------------------------------------
 Raw HFC matrix (all values in MHz):
 ----------------------------------
                0.2749            0.0683           -0.0031
                0.0683           -0.1554           -0.0940
               -0.0031           -0.0940           -0.6969

 A(FC)         -0.1925           -0.1925           -0.1925
 A(SD)          0.0421            0.4782           -0.5203
            ----------        ----------        ----------
 A(Tot)        -0.1504            0.2858           -0.7128    A(iso)=   -0.1925
 Orientation:
  X          -0.1576343         0.9874609         0.0085057
  Y           0.9733652         0.1568245        -0.1672313
  Z          -0.1664683        -0.0180822        -0.9858810

Notes:  (1) The A matrix conforms to the "SAI" spin Hamiltonian convention.
        (2) Tensor is right-handed.

 -----------------------------------------------------------
 Nucleus  73H : A:ISTP=    1 I=  0.5 P=533.5514 MHz/au**3
                Q:ISTP=    2 I=  1.0 Q=  0.0029 barn
```

```
 ----------------------------------------------------------
 Raw HFC matrix (all values in MHz):
 -----------------------------------
               -0.9009             -0.8215              0.1404
               -0.8215              2.4121             -0.6942
                0.1404             -0.6942             -1.0390

 A(FC)          0.1574              0.1574              0.1574
 A(SD)         -1.2454             -1.3345              2.5799
             ----------          ----------          ----------
 A(Tot)        -1.0880             -1.1771              2.7373    A(iso)=    0.1574
 Orientation:
  X          0.9540705           0.1997837           0.2232397
  Y          0.1758154           0.2299585          -0.9571876
  Z         -0.2425664           0.9524734           0.1842715

Notes:  (1) The A matrix conforms to the "SAI" spin Hamiltonian convention.
        (2) Tensor is right-handed.

 ----------------------------------------------------------
 Nucleus  80H : A:ISTP=    1 I=  0.5 P=533.5514 MHz/au**3
               Q:ISTP=    2 I=  1.0 Q=  0.0029 barn
 ----------------------------------------------------------
 Raw HFC matrix (all values in MHz):
 -----------------------------------
               -0.7902              1.0231             -0.1812
                1.0231              2.2989             -0.6931
               -0.1812             -0.6931             -1.0437

 A(FC)          0.1550              0.1550              0.1550
 A(SD)         -1.2457             -1.3417              2.5874
             ----------          ----------          ----------
 A(Tot)        -1.0907             -1.1867              2.7424    A(iso)=    0.1550
 Orientation:
  X          0.9342049          -0.2183385          -0.2821162
  Y         -0.2254340           0.2515837          -0.9412147
  Z          0.2764793           0.9428860           0.1858097

Notes:  (1) The A matrix conforms to the "SAI" spin Hamiltonian convention.
        (2) Tensor is right-handed.

 ----------------------------------------------------------
 Nucleus  83H : A:ISTP=    1 I=  0.5 P=533.5514 MHz/au**3
               Q:ISTP=    2 I=  1.0 Q=  0.0029 barn
 ----------------------------------------------------------
 Raw HFC matrix (all values in MHz):
 -----------------------------------
                0.2730             -0.0896              0.0098
               -0.0896             -0.1591             -0.1018
                0.0098             -0.1018             -0.6996

 A(FC)         -0.1952             -0.1952             -0.1952
 A(SD)          0.0359              0.4870             -0.5229
             ----------          ----------          ----------
 A(Tot)        -0.1593              0.2918             -0.7181    A(iso)=   -0.1952
 Orientation:
  X          0.2036823           0.9790153          -0.0065199
  Y          0.9627780          -0.2015039          -0.1801517
  Z         -0.1776851           0.0304165          -0.9836172

Notes:  (1) The A matrix conforms to the "SAI" spin Hamiltonian convention.
        (2) Tensor is right-handed.

 ----------------------------------------------------------
 Nucleus  84H : A:ISTP=    1 I=  0.5 P=533.5514 MHz/au**3
               Q:ISTP=    2 I=  1.0 Q=  0.0029 barn
```

```
 ----------------------------------------------------------
 Raw HFC matrix (all values in MHz):
 ----------------------------------
              -0.4540              -0.0112              -0.0591
              -0.0112               0.1443              -0.1552
              -0.0591              -0.1552              -0.4795

 A(FC)        -0.2631              -0.2631              -0.2631
 A(SD)         0.4438              -0.1543              -0.2895
            ----------           ----------           ----------
 A(Tot)        0.1807              -0.4173              -0.5526    A(iso)=   -0.2631
 Orientation:
  X         0.0042340           -0.8538109            0.5205661
  Y         0.9733848            0.1228023            0.1934982
  Z        -0.2291376            0.5058919            0.8316065

Notes:  (1) The A matrix conforms to the "SAI" spin Hamiltonian convention.
        (2) Tensor is right-handed.

 ----------------------------------------------------------
 Nucleus  85H : A:ISTP=    1 I=  0.5 P=533.5514 MHz/au**3
              Q:ISTP=    2 I=  1.0 Q=  0.0029 barn
 ----------------------------------------------------------
 Raw HFC matrix (all values in MHz):
 ----------------------------------
               0.4106              -0.0378               0.0064
              -0.0378               0.3102              -0.0844
               0.0064              -0.0844               0.0208

 A(FC)         0.2472               0.2472               0.2472
 A(SD)        -0.2493               0.0702               0.1791
            ----------           ----------           ----------
 A(Tot)       -0.0021               0.3174               0.4263    A(iso)=    0.2472
 Orientation:
  X         0.0091325            0.3789822           -0.9253589
  Y         0.2619320            0.8921791            0.3679784
  Z         0.9650431           -0.2457417           -0.0911198

Notes:  (1) The A matrix conforms to the "SAI" spin Hamiltonian convention.
        (2) Tensor is right-handed.
```

References

[1] Z. Zeng, Y. M. Sung, N. Bao, D. Tan, R. Lee, J. L. Zafra, B. S. Lee, M. Ishida, J. Ding, J. T. L. Navarrete, Y. Li, W. Zeng, D. Kim, K.-W. Huang, R. D. Webster, J. Casado, and J. Wu. Stable tetrabenzo-chichibabin's hydrocarbons: tunable ground state and unusual transition between their closed-shell and open-shell resonance forms. *Journal of the American Chemical Society*, 134(35):14513–14525, 2012.

[2] K. Tanaka, S. Kishigami, and F. Toda. A new method for coupling aromatic aldehydes and ketones to produce. alpha.-glycols using zinc-zinc dichloride in aqueous solution and in the solid state. *The Journal of Organic Chemistry*, 55(9):2981–2983, 1990.

[3] H. Lee, M. Jo, G. Yang, H. Jung, S. Kang, and J. Park. Highly efficient dual anthracene core derivatives through optimizing side groups for blue emission. *Dyes and Pigments*, 146:27–36, 2017.

[4] T. Y. Baum, S. Fernández, D. Peña, and H. S. J. Van Der Zant. Magnetic fingerprints in an all-organic radical molecular break junction. *Nano Letters*, 22(20):8086–8092, 2022.

[5] A. Schweiger and G. Jeschke. *Principles of pulse electron paramagnetic resonance*. Oxford University Press, 2001.

[6] C. Gemperle and A. Schweiger. Pulsed electron-nuclear double resonance methodology. *Chemical reviews*, 91(7):1481–1505, 1991.

[7] G. Jeschke and A. Schweiger. Hyperfine-correlated electron nuclear double resonance spectroscopy. *Chemical physics letters*, 246(4-5):431–438, 1995.

[8] S. Stoll and A. Schweiger. Easyspin, a comprehensive software package for spectral simulation and analysis in epr. *Journal of magnetic resonance*, 178(1):42–55, 2006.

[9] X. Yin, J. Z. Low, K. J. Fallon, D. W. Paley, and L. M. Campos. The butterfly effect in bisfluorenylidene-based dihydroacenes: aggregation induced emission and spin switching. *Chemical science*, 10(46):10733–10739, 2019.

[10] S. Dong and Z. Li. Recent progress in open-shell organic conjugated materials and their aggregated states. *J. Mater. Chem. C*, 10:2431–2449, 2022.

[11] F. Neese. The orca program system. *Wiley Interdisciplinary Reviews: Computational Molecular Science*, 2(1): 73–78, 2012.

[12] E. F. Pettersen, T. D. Goddard, C. C. Huang, E. C. Meng, G. S. Couch, T. I. Croll, J. H. Morris, and T. E. Ferrin. Ucsf chimerax: Structure visualization for researchers, educators, and developers. *Protein Science*, 30(1): 70–82, 2021.
